# Phytochemical analysis, biological activities of methanolic extracts and an isolated flavonoid from Tunisian *Limoniastrum monopetalum* (L.) Boiss: an in vitro and in silico investigations

**DOI:** 10.1038/s41598-023-46457-6

**Published:** 2023-11-06

**Authors:** Amel Bouzidi, Ahmed Azizi, Omar Messaoudi, Kirouani Abderrezzak, Giovanni Vidari, Ahmed Noureddine Hellal, Chirag N. Patel

**Affiliations:** 1 BTP Laboratory, Department of Biology, Faculty of Sciences, University of Medea, Médéa, Algeria; 2 Faculty of Technology, University Amar Telidji, Highway Ghardaia, Post Box G37 (M’kam), 03000 Laghouat, Algeria; 3 Laboratory of Applied Microbiology in Food, Biomedical and Environment, Abou Bekr Belkaïd University, 13000 Tlemcen, Algeria; 4 Department of Biology, Faculty of Science, University of Amar Telidji, 03000 Laghouat, Algeria; 5 Department of Medical Analysis, Faculty of Applied Science, Ishk International University, Erbil, 44001 Iraq; 6 Laboratory of Bioressources, Biology Integrative and Valorization, Higher Institute of Biotechnology of Monastir, University of Monastir, Monastir, Tunisia; 7 Department of Botany, Bioinformatics and Climate Change Impacts Management, School of Science, Gujarat University, Ahmedabad, Gujarat 380009 India; 8 Biotechnology Research Center, Technology Innovation Institute, 9639 Abu Dhabi, United Arab Emirates

**Keywords:** Computational biology and bioinformatics, Microbiology, Plant sciences

## Abstract

In recent years, due to the dramatic increase of the bacteria resistance to antibiotics and chemotherapeutic drugs, an increasing importance is given to the discovery of novel bioactive molecules, more potent than those in use. In this contest, methanol extracts of different parts of the medicinal plant *Limoniastrum monopetalum* (L.) Boiss. (Plumbaginaceae), widely occurring in Tunisia, were prepared to evaluate the antimicrobial and antiproliferative activities. The methanol extract of the roots showed the highest antibacterial activity against *E. coli*, *S. aureus* and *E. faecalis*, whereas the stem extract exhibited the highest antiproliferative effects towards a Hela cell line. Analysis of volatile fractions, using gas chromatography–mass spectrometry (GC–MS) and gas chromatography–flame ionization detector (GC–FID) techniques, led to the identification of camphor as the most abundant constituent, which represented from 84.85 to 99.48% of the methanol extracts. Multiple chromatographic separation of the methanol leaf extract afforded the flavonoid maeopsin-6-*O*-glucoside (S1) and a few fractions that were subjected to biological activity assays. One fraction exhibited interesting antibacterial activity against *E. coli* and *E. faecalis* (MIC values of 62.5 and 78.12 µg/mL, respectively), and antiproliferative effects against Hela and A549 cells (IC_50_ = 226 and 242.52 μg/mL, respectively). In addition*, *in silico studies indicated that maesopsin-6-*O*-glucoside, which was moderately active against *Staphylococcus aureus*, strongly interacted with the active site of the accessory gene regulator protein A (AgrA) of *Staphylococcus aureus*.

## Introduction

Many drugs currently in use have become less effective against many infectious and cancerous diseases, due to rising resistance to antibiotics and chemotherapeutic drugs, respectively. Consequently, active efforts are directed towards the discovery of new bioactive molecules, more effective than those in use. Despite the large number of bioactive compounds discovered by combinatorial chemistry or other synthetic chemistry methods, natural products, and their derivatives are still a precious source of care for humans, due to their efficacy and a few side effects^1–3^. The medicinal values of plants depends on the presence of phytochemical bioactive components such as alkaloids, tannins, flavonoids, and phenolic compounds^4^. Polyphenols are the most common group of plant-based bioactive compounds play an interesting role^5^ and are believed to protect human health, against various diseases, including cardiovascular troubles, diabetes, some immunological disorders, and cancers^6,7^.

In this context, plant flavonoids have attracted attention as potentially important dietary cancer chemoprotective agents, and some of them exhibited a good antitumoral effect, against certain cancer type^8–10^. The plant *Limoniastrum monopetalum (L.) Boiss*. is a perennial halophyte, growing at different habitats, along the coast of the Mediterranean coasts, including Tunisia^11^. Leaf and gall infusions of *L. monopetalum* are conventionally expended for the treatment of different infectious diseases that cause pain and bloody diarrhea^12^. Moreover, several studies have shown that *Limoniastrum monopetalum L*. leaves contain a significant content of phenolic compounds exhibiting good antioxidant activity^13–16^. Insufficient knowledge about the secondary metabolites in L. monopetalum spurred us to conduct an in-depth investigation into the chemical composition of various plant parts, including volatile fractions. Additionally, we assessed the plant's biological properties by examining the impact of extracts, chromatographic fractions, and an isolated metabolite on the proliferation of Hela (Human cervical adenocarcinoma) and A549 (Human lung carcinoma) cell lines. We also investigated its antibacterial potential against specific pathogenic microorganisms, with a particular focus on its effectiveness against methicillin-resistant strains of Staphylococcus aureus (MRSA). In fact, this pathogen has recently become a serious menace to human health worldwide, due to the exceptional ability to acquire resistance to a wide range of antibiotics, including vancomycin, which is one of the first line drugs used to treat MRSA infections^17^.

The severity of *S. aureus* infections hinges on the controlled production of virulence factors that are vital for the microbe's survival within the host. These factors encompass cell surface adhesins, extracellular enzymes, and toxins. Among the well-studied regulatory systems governing virulence is the accessory gene regulator (agr), which operates as a quorum-sensing (QS) system. Upon activation of the agr system, the pathogen releases the extracellular peptide signal Agr, allowing the bacterium to sense its local population density^18^. When the bacterial cell density reaches a critical 'quorum,' this information triggers a specific gene expression pattern, resulting in the production of several extracellular toxins and cell adhesins^19^. In light of this, there is growing interest in small compounds that can inhibit bacterial virulence as alternatives or supplements to conventional antibiotics. Such compounds have the potential to attenuate pathogenesis and enhance bacterial susceptibility to host defenses. In this context, maesopsin-6-*O*-glucoside, a flavonoid glucoside isolated from *L. monopetalum,* displayed intriguing in vitro activity against *S. aureus*. Consequently, the latter part of our research delved into investigating the inhibitory potential of this metabolite against the response regulator AgrA protein of Staphylococcus aureus through an in silico study.

In summary, this paper contains: (i) the GC–MS and GC–FID analyses of volatile fractions isolated from the methanol extracts of leaves, stems and roots of *L. monopetalum*; (ii) the fractionation of the leaf extract on a Sephadex LH-20 column; (iii): the results of in vitro assays of the antiproliferative and antibacterial effects of isolated chromatographic fractions and the flavonoid maesopsin-6-*O*-glucoside; (iv) the results of an in silico investigation of the interactions of maesopsin-6-*O*-glucoside with the accessory gene regulator protein A (AgrA) of *Staphylococcus aureus*.

## Materials and methods

All methods were performed in accordance with the relevant guidelines/regulations/legislation.

### Chemical reagents and materials

ACS-grade solvents (absolute methanol, ethanol and acetone) employed in the extraction processes and HPLC-grade solvents utilized in chromatographic separations were procured from Carlo Erba Reagenti (Milan, Italy). 3-(4,5-dimethylthiazol-2-yl)-2,5-diphenyltetrazolium bromide (MTT), DMSO and other reagents, as well as streptomycin and penicillin were obtained from Sigma-Aldrich (GmbH, Steinheim, Germany). Dulbecco’s Modified Eagle Medium (DMEM) was acquired from Gibco /Thermo Fischer Scientific—US. ^1^H and ^13^C NMR spectra were determined in MeOH-d_4_ (Sigma-Aldrich, Steinheim, Germany) on an Advance 200 MHz spectrometer or, alternatively, on an Advance 400 MHz spectrometer (Bruker, Bremen, Germany), with TMS as an internal standard. Bidimensional NMR experiments were recorded with standard pulse sequences. GC–FID analyses were conducted using a PerkinElmer Auto System gas chromatograph. ESI–MS spectra were acquired using a Thermo Scientific LTQ XL Linear Ion Trap mass spectrometer equipped with a heated ESI source.

Solid phase extraction (SPE) was conducted on Discovery DSC 18 (RP-C18) 60 mL tubes, 10 g capacity, purchased from Supelco. Normal-pressure preparative column chromatographic separations were carried out on Merck LiChroprep RP-18 (25–40 μm) C-18 reversed phase or on Merck Kieselgel 60 silica gel (230–400 mesh). Medium-pressure liquid chromatographic (MPLC) separations were conducted on a ISOLERA™ One—Biotage^®^ instrument equipped with commercial columns, a double piston pump and a dual-wavelength UV–Visible detector. Sephadex LH-20 powder was purchased from Sigma-Aldrich (Steinheim, Germany).

### Plant material

*Limoniastrum monopetalum* was collected in November from Monastir (Skanes region, Tunisia). The plant was identified by a specialist in botany at the Faculty of Sciences of Bizerte, Laboratory of Botany and Plant Ecology and stored at the Laboratory of Bioresources, Biology Integrative and Valorization, Higher Institute of Biotechnology of Monastir. Subsequently, the different parts of the plant were separated and dried for three days, in an oven set at 47 °C.

### Preparation of plant extracts

#### Extraction

The *Limoniastrum monopetalum,* methanol was obtained through the maceration method to study the thermolabile components^20^. Accurately dried and finely ground leaves (1 kg), stems (1 kg), and roots (0.5 kg) of *Limoniastrum monopetalum,* were separately macerated^20^ in MeOH under stirring at room temperature for three days. After filtration, the extracts were evaporated to dryness under vacuum to give three residues (A–C) of 45, 25 and 13.8 g, respectively^20^.

#### Chlorophyll removal from residue A

To remove chlorophylls, possibly interfering in subsequent chromatographic separations, a sample (14 g) of residue A was divided in portions of 1 g each, which were dissolved in 60 ml of MeOH/H_2_O (80:20). Afterward, each solution was filtered at reduced pressure through a *C*_*18*_* solid phase extraction* (SPE) cartridge. The filtrates were pooled together and evaporated to give a chlorophyll free residue (A’, 8 g).

### Extraction and analysis of volatile compounds from *L. monopetalum*

#### Isolation of volatiles from extracts of *L. monopetalum* by hydrodistillation

Three samples (4 g each) of residues A–C were separately suspended in distilled water (50 mL), and each suspension was subjected to hydrodistillation for 1 h in a Clevenger-type apparatus. A white precipitate (P1), which spontaneously separated from the condensed aqueous mixture, was separated by filtration, while the aqueous layer was filtered through a pad (1 g) of C-18 reversed phase. The fraction attached to the stationary phase was eluted with MeOH (2 mL). Three volatile fractions V1–V3 were thus obtained from A–C, that were subsequently analyzed by GC–MS and GC–FID techniques.

####  Analysis of volatiles fractions V1–V3 by GC–MS

GC–MS analyses were performed on a HP-5 fused silica non-polar capillary column (30 m × 0.25 mm i.d., film thickness 0.25 µm), under the following operating conditions: injector temperature, 250 °C; carrier gas, He; flow rate, 1 mL/min; oven temperature program, isothermal at 60 °C for 3 min, followed by a temperature ramp of 5 °C/min up to 260 °C, and then held at 260 °C for 15 min. The mass scan range was covered 41–350 amu; sample/solvent ratio, 1:20; injection volume, 1 µl in split mode (10:1); ionization energy, 70 eV. Methanol solutions of each volatile fraction were separately analyzed by GC–MS under identical conditions. To calculate the Linear Retention Index (LRI) of each volatile compound, a standard mixture of *n*-alkane homologues from *n*-heptane (C_8_) to *n*-nonadecane (C_19_), purchased from Sigma-Aldrich, were injected under identical chromatographic conditions, immediately after each fraction analysis^21^.

#### Analysis of volatile fractions V1–V3 by CPG-FID

The GC–FID instrument was equipped with a HP-5 capillary column (length: 25 m, internal diameter: 0.25 mm, thickness of the stationary phase: 0.25 µm); nitrogen was the carrier gas at 1 mL/min; the injector was set at 260 °C and operated in the split mode, with a split ratio of 10; the detector temperature was set at 260 °C; oven temperature program: isothermal kept at 60 °C for 3 min, subsequently the temperature was increased to 150 °C with a gradient rate of 5 °C/min, followed by a gradient rate of 10 °C/min to 260 °C, finally it was kept at 260 °C for an additional 1 min.

#### Identification and quantification of volatiles

Each component of the volatile fractions V1–V3 (Table 1) was identified by comparing the corresponding mass spectrum with the spectra contained in the Adams^22^ and NIST 08^23^ libraries, as well as by comparing the calculated linear retention index (LRI_exp_)^21^, with the literature (LRI_lit_)^22,23^. The identification of most oil components was confirmed by coelution with authentic standards (Sigma-Aldrich, Milan). The relative amount of each component of the volatile fractions (Table 3) was calculated as the percent of the corresponding peak area on the FID-gaschromatogram with respect to the total area of peaks, without using a correcting response-factor. Mean % abundances and standard deviations were determined from the results of three replicates for each fraction. Data were collected with HP3398A GC Chemstation software (Hewlett-Packard, Rev. A.01.01).

**Table 1:** Chemical composition of the volatile fractions V1–V3 from the methanol extracts of *L. monopetalum* leaves, stems and roots.

Compound name^a^	LRI_exp_^b^	LRI_lit_^c^	V1^d^	V2^d^	V3^d^
Fenchone	1087	1086	–	0.11 ± 0.02	0.36 ± 0.12
*cis*-Thujone	1098	1095	–	–	2.88 ± 0.23
Unidentified	1103	–	0.33 ± 0.11	1.32 ± 0.12	7.27 ± 0.55
Unidentified	1117	–	0.03 ± 0.01	0.17 ± 0.05	0.72 ± 0.15
Camphor (**2**)	1143	1141	99.47 ± 1.51	96.45 ± 1.38	84.58 ± 1.22
Karahanaenone	1156	1154	–	0.22 ± 0.06	0.16 ± 0.05
Unidentified	1159	–	–	0.24 ± 0.08	0.34 ± 0.08
Isoborneol	1164	1155	–	0.09 ± 0.02	0.14 ± 0.04
5-Methylene-2,3,4,4-tetramethyl- cyclopent-2-enone	1191	1183	0.01 ± 0.004	1.39 ± 0.15	3.54 ± 0.17
4-Methylene- isophorone	1215	1216	0.15 ± 0.03	–	–
Indole	1291	1290	0.01 ± 0.002	–	–

^a^Compounds are listed in order of elution from a HP-5 column; ^b^linear retention index on a HP-5 column, experimentally determined in accordance with reference 21; ^c^linear retention index taken from the literature^22,23^ for a non-polar column; ^d^% content ± SD of each component (n = 3) in the corresponding volatile fraction V.

### Chromatographic analysis

#### Chromatographic separation of leaf methanol extract

With the aim to purify and identify the bioactive components present in extract A, residue A’ (8 g) was dissolved in a mixture of MeOH/H_2_O, 80:20, (15 mL). Subsequently a sample of the solution (3 mL) was separated on Sephadex LH-20 (100 g) contained in a glass column 60.5 cm long, with a diameter of 3.2 cm. The stationary phase was swollen in MeOH/H_2_O (80:20) for 24 h at 4 °C. Elution was conducted at a flow of 3 mL/min with a mixture of EtOH/H_2_O (80:20, 710 mL), followed by acetone/H_2_O (50:50, 430 mL)^24^. The same process was repeated for the remaining solution of A’.

#### Thin layer chromatography (TLC) analysis

The extracts and the chromatographic fractions were analyzed on TLC glass plates pre-coated with C18 reversed phase (Silica gel 60 RP18 F254S from Merck) and developed with MeOH-H_2_O, 50:50. Spots were visualized under UV light at 254 and 366 nm and by spraying with H_2_SO_4_/vanillin reagent (1 g vanillin dissolved in 60 mL of 96% EtOH and 160 mL of H_2_SO_4_), followed by heating with a hot gun. The fractions with similar composition were pooled together to give eight main fractions (F1–F8). The TLC analysis showed that F1 contained only tannins and was discarded; instead, F2–F8 evaporated to dryness and subjected to in vitro assays.

#### Separation of fraction F6 by MPLC and isolation of maesopsin-6-*O*-glucoside (1)

The fraction F6 (800 mg) was separated by MPLC using a Biotage^®^ Isolera instrument equipped with a Biotage^®^ prepacked C_18_ cartridge (100 g). The flow rate was set at 20 mL/min. The binary mobile phase consisted of H_2_O and MeOH. The gradient elution program started with 10% H_2_O–90% MeOH (15 min), followed by 10–50% H_2_O–90–50% MeOH (55 min), and ended with 50% H_2_O – 50% MeOH (17 min). The dual UV detector was set at 204 and 254 nm. 24 sub fractions (SF1-SF24) were obtained, whose TLC analysis showed different chemical composition. Preparative TLC purification of SF16 (11.7 mg) on a C18 layer, eluted with H_2_O-MeOH, 50:50, afforded pure compound S1 (8 mg), identical with maesopsin-6-*O*-β-d-glucoside (1).

#### Spectroscopic analysis

Pure compound, was analyzed by ^1^H, recorded on an Advance 200 MHz spectrometer or alternatively on Avance 300 MHz spectrometer (Bruker, Bremen, Germany), with TMS as an internal standard in NMR spectroscopy. MS spectra were performed on a Thermo Scientific LTQ XL Linear Ion Trap mass spectrometer, equipped with a heated ESI source. The medium-pressure liquid chromatography (MPLC) Biotage ISOLERA One with Biotage commercial columns.

### Biological activities

#### Antibacterial activities assay

The antibacterial activity of residues A’, B and C, fractions F2-F8 and compound S1 was tested by the microdilution method^24^, against a panel of four oral pathogenic bacteria: *Escherichia coli* (ATCC 25922), *Pseudomonas aeruginosa* (ATCC 25923), *Staphylococcus aureus* (ATCC 27853) and *Enterococcus faecalis* (ATCC 29212). These microorganisms were provided by the laboratory of Parasitology-Mycology and the laboratory of Microbiology, Fattouma BOURGUIBA Hospital of Monastir, Tunisia. For the preparation of suspension, the bacterial test microorganisms were inoculated in Mueller Hinton Broth (MHB). After 24 h incubation at 37 °C, the optical density of suspensions at 600 nm were adjusted between 0.08 and 0.1 (measured at 600 nm), which corresponded to the microbial density between 10^6^ CFU/mL and 10^8^ CFU/mL. The antibacterial activity was assessed by determining the minimal inhibitory concentrations^25,26^ was measured in 96-well microtiter plates, after 24 h of samples incubation at 37 °C. The MIC is defined as the lowest extract concentration inhibiting a visible growth of each microorganism. Each assay was conducted in triplicate and the mean MIC ± SD was calculated.

The antibacterial activity was assessed by determining the minimal inhibitory concentrations (MIC) in 96-well microtiter plates through microtiter dilution^26,27^. In this method, each well of the microtiter plate was inoculated with 150 μL of the tested microorganism suspension. Furthermore, 130 µL of the same microbial suspension was introduced into wells of A1–A10. Subsequently, 20 μL of the stock solution prepared for residues A', B, and C, as well as fractions F2–F8 and the pure compound S1, were added to the wells A1–A9 on the microtiter plate, resulting in a final volume of 300 μL for each well. The pure compound S1 was added to the first well to achieve a final concentration of 500 μg/mL, while the residues A’, B and C and fractions F2–F8, were introduced into the first well at a final concentration of 5000 μg/mL. Subsequently, a serial dilution of 1/2 was performed by transferring 150 µL from row A-H, and the remaining 150 µL were discarded. The microtiter plate was incubated at 37 °C for 24 h, and the MIC was determined as the lowest concentration inhibiting a visible growth of each tested microorganism. Each assay was conducted in triplicate and the mean MIC ± SD was calculated. Vancomycin and imipenem were used as a positive control for antibacterial activity.

#### Antiproliferative activity assay

##### Cell culture

Hela (derived from human cervical cancer) and A549 cells (derived from human lung cancer), obtained from the American Type Culture Collection (ATCC, Rockville, MD, USA), were inoculated in DMEM (Dulbecco’s Modified Eagle) medium, containing 10% fetal bovine serum (FBS), 1% (w/v) glutamine and antibiotics (10,000 U/mL penicillin and 100 µg/mL streptomycin). The incubation was carried out under a 5% CO_2_ atmosphere at 37 °C.

##### MTT assay

Cell viability assay was measured using the MTT test with slight modifications^27^. Each well of a 96-well plate (Greiner bio-one), containing 200 μL of growth medium, was inoculated with 5 × 10^3^ cells. Cells were permitted to adhere for 24 h; subsequently, they were exposed for 48 h to each sample, at concentrations of 125, 250, 500, 800, 1000 µg/mL in growth medium. Subsquently, 10 μL of MTT in phosphate buffered saline (PBS) (5 mg/ml) was added to each well and the plate was incubated for an additional 2 h. The medium was removed and the precipitate of formazan blue, formed in the cells, was dissolved in dimethyl sulphoxide (100 μL). After incubation at 37 °C for 10 min, the absorbance A of the solution at 550 nm was measured by a microplate ELISA reader (Thermo Labsystems).

The control cells were maintained in complete medium. This assay was conducted in triplicate and cell viability was expressed as the relative formazan formation in the treated samples as compared to control cells. The percentages of cell growth were calculated as follow:

$$ {\text{Cell growth }}\left( \% \right) \, = \, \left[ {{\text{A }}\left( {\text{treated cells}} \right)/{\text{A }}\left( {\text{control cells}} \right)} \right] \, \times { 1}00 $$

where A (control cells) is the absorbance at 550 nm of control cells which were not exposed to tested sample. The antiproliferative activity was expressed as IC_50_, which was the concentration (μg/mL) of sample inhibiting cell growth by 50%. Each test was conducted in triplicate and the mean ± SD was calculated.

##### Statistical analysis

The data of the antiproliferative activity were subjected to analysis of the variance (ANOVA) by the software Statistica Version 7. The comparison of the means was done according to Fisher's Least Significant Difference (LSD).

### In silico study

#### Molecular docking analysis

Molecular docking calculations were performed with the program AutoDock Tools 1.5.4, using the Lamarckian Genetic Algorithm. The AutoGrid was used to generate a grid box size of 40 × 40 × 40 Å points with a grid spacing of 1 Å, centered at x, y, z coordinates of 17.215 Å, 15.708 Å, 44.832 Å, around the hotspots residues of the active site of the accessory gene regulator protein A (AgrA) (4g4k) of *Staphylococcus aureus*. The employed docking parameters for each docked compound were derived from 100 independent docking runs that were set to terminate after a maximum of 2.5 × 10^6^ energy evaluations with mutation rate of 0.02 and crossover rate of 0.8. The population size was set to use 250 randomly placed individual. The Lamarckian genetic algorithm was used, and the output was saved in docking parameter file (DPF) format. The predicted binding poses for each compound S1 were processed by the 0-clustering analysis (1.0 Å RMSD tolerance) and the lowest energy conformation from the largest cluster was selected as representative docked complex. Discovery Studio Visualizer and PyMOL were implemented to visualize and scrutinize the interaction between the ligand fragments with aromatase protein^28^.

####  Molecular dynamics (MD) simulations study

Molecular dynamics modelling was used to determine the physical motions of atoms and molecules in a protein–ligand (4g4k-ligand S1) docked complex. MD simulation was performed at 1000 ns time intervals. Desmond (Schrodinger Release 2019-3) was used for a study of molecular dynamics simulations. Facilitating complex relaxation, these complexes have been generated using a protein preparation wizard. The insertion of hydrogens, removal of water, assignment of bond ordering, and filling in missing side chains and loops with optimization of hydrogen-bond assignment (sampling of water orientations and usage of pH 7.0) were all carried out. The simulation cell was created with a system builder module and a TIP3P (transferable intermolecular potential with three points) water model with a simulation box size of 10 Å × 10 Å × 10 Å and an all-atom force field 2005 from Optimized Potentials for Liquid Simulations (OPLS). The system was reduced for 1000 iterations using steepest descent minimization while the NPT (number of atoms, pressure, and temperature were held constant) ensemble was running with 300 K and 1.01 bar, constant volume, Smooth Particle Mesh Ewald (PME) technique. Following that, the histogram for torsional bonds, root mean square deviation (RMSD), root mean square fluctuation (RMSF), Hydrogen bond, a radius of gyration (Rg), and a radius of gyration (Rg) were analyzed for the identification of structural changes associated with the dynamic role of the selected protein–ligand complexes^29^.

#### Binding free energy calculations

The binding free energy was calculated using molecular mechanics generalized Born surface area (MM/GBSA) utilizing a single trajectory technique. The MM/GBSA computations include 1000 ns MD simulations of the best docked protein–ligand complexes. The following Eqs. (1, 2) were used to determine the free energy values:

1 $$ \Delta G_{bind} = \Delta G_{complex(minimized)} {-} \, [\Delta G_{ligand(minimized)} + \Delta G_{receptor(minimized)} ] $$

and

2 $$ \Delta G_{bind} = \Delta G_{MM} + \Delta G_{PB} + \Delta G_{SA - } T\Delta S_{{}} $$

where Δ_*TDS*_ is the conformation entropic contribution, and Δ*G*_*MM*_ is the molecular mechanics' interaction energy (electrostatic + van der Waals interaction) between protein and ligand. Δ*G*_*PB*_ and Δ*G*_*SA*_ depict the polar solvation energy and the nonpolar solvation energy, respectively.

### Declaration

No specimen of *Limoniastrum monopetalum* (L.) Boiss*.* has been deposited in a public herbarium, as we did not work in a specialized laboratory. The plant is a spontaneous herbaceous species, which grows abundantly on the coastal areas of Tunisia, where it is not protected and the collection requires no permission for research purposes. The experimental protocols followed to study extracts of this plant are referenced, and results are compared with other works. For some experiments, the microorganisms and strains used are referenced as noted in the work. The cytotoxic activity and MD simulation data were mentioned in supplementary file. For the rest of the activities the data is included in the paper. Overlapping passages in the Methods do not include our optimization methods. In fact, we performed our proper tests and used multiple optimized parameters, such as (temperature, injection mode and exposure time to multiple degrees. Original source of method descriptions is not assigned to a precise person or previously described protocols**.**

## Results and discussion

### Structure elucidation of isolated compound S1

**Figure 1:**
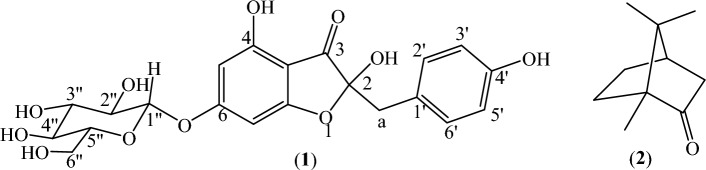
Chemical structure of maesopsin-6-*O*-glucoside (**1**) and camphor (**2**).

Multiple chromatographic separations of the residue A’ from the methanol extract of *L. monopetalum* leaves afforded pure compound S1. The molecular formula of S1 was determined as C_21_H_22_O_11_ (MW = 450) from the pseudomolecular ion peak [M + Na]^+^ at *m*/*z* 473.16 in the HPLC–UV-HRESIMS spectrum (Fig. S1 in the Supplementary Information). The IR spectrum of S1 showed a peak at 1685 cm^−1^ assignable to an aromatic ketone. The ^1^H NMR spectrum (400 MHz) of S1 (Fig. S2 and Table S1 in the Supplementary Information) showed two *meta*-coupled (*J* = 1.5 Hz) aromatic protons (H-5 and H-7), an AA’BB’ system for four aromatic protons (H-2’, H-3’, H-5’, H-6’) on a *para*-substituted aromatic ring, a singlet (*δ*_H_ 3.06, 2H) attributable to an isolated benzylic methylene group, and the characteristic signals of a β-d-glucosyl residue [multiplets between *δ*_H_ 3.35–3.9 for H-2”–H_2_-6”, and a complex signal at about *δ*_H_ 4.85 (*J* = 8 Hz) for H-1”]. The data were identical to those of the known flavonoid maesopsin-6-*O*-glucoside (Fig. 1), isolated for the first times in 1997 by Li et al.^30^ from the root bark of *Ceanothus americanus* (family Rhamnaceae)^32^. Different protons of compound **1**, e.g., H-1”, showed two sets of equally intense signals in the NMR spectrum, due to the reversible nature of the hemiketal at C-2. Therefore compound **1** consisted of a pair of diastereomers, to which was assigned the structure of (2*R*,*S*)-2,4-dihydroxy-2-[(4-hydroxyphenyl)methyl]-6-[(2*S*,3*R*,4*S*,5*S*,6*R*)-3,4,5-trihydroxy-6-(hydroxymethyl)oxan-2-yl]oxy-1-benzofuran-3-one^32^.

The compound exhibited antimicrobial activity against selected oral Gram-negative pathogens, anaerobic periodontal bacteria and Gram-positive cariogenic bacteria, only at concentrations above 500 µg/ml^31^.

### Chemical composition of the volatile fractions V1–V3

The three residues A, B and C from the methanol extracts of different parts of *L. monopetalum* had a pleasant camphoraceous smell. The volatile fractions V1–V3, isolated from the residues by hydrodistillation, were analyzed by GC–FID and GC–MS techniques. A total of eleven components (Table 1) were detected, eight of which were identified by comparing the corresponding mass spectrum and the calculated linear retention index (LRI_exp_)^21^, with the literature^22,23^. The identity of most components was also confirmed by coelution with authentic standards. All the fractions were of monoterpenoid type, except for the minimal amounts of indole in V1. Camphor (**2**) was the largely predominant component of all fractions, and it precipitated in the aqueous distillates. The precipitate (P1) was especially abundant (200 mg) in the distillate from the root methanol extract (V3). The identity of P1 with camphor was confirmed by NMR and MS spectra. The composition of V1–V3 markedly differed from the volatiles of other *Limoniastrum* species, for example from the essential oils of *L. gyonianum* leaves, flowers, seeds, and roots^33^ Moreover, the high content of camphor (**2**), which exhibited a wide range of anti-fungal and antibacterial activities^34,35^, could contribute to the antimicrobial and antiproliferative effects of the methanol extracts (see below), although phenolic derivatives were likely the main contributors to the activities^36,37^.

### Antibacterial activity

In search for new antibacterial agents, the residues A’, B and C from the methanol extracts of leaves, stems and roots of *L. monopetalum*, respectively, as well as the fractions F1–F8 and compound S1, maesopsin-6-*O*-glucoside (**1**), resulting from the separation of residue A’, were tested in vitro against a pair of gram-negative and a pair of gram-positive bacteria. The experimental MIC values (μg/mL) are reported in the Table 2. The tested samples exhibited a wide range of antibacterial activities, likely due to varying chemical contents. Among the residues, the residue C from the methanol extract of the roots showed the highest activity against *E. coli*, *S. aureus* and *E. faecalis* with MIC values of 83 µg/mL, while the growth of *P. aeruginosa* was inhibited by the residues B and C from the methanol extracts of the stems and roots with the same MIC of 150 µg/mL. Quite remarkable were also the antibacterial activity of the flavonoid S1, maesopsin-6-*O*-glucoside (**1**) against *S. aureus* and *P. aeruginosa* with a MIC of 67 µg/mL. as well as the inhibitory effects of fractions F7 and F8 against the gram- *E. coli* and the gram + *E. faecalis,* with MIC values in the range of 67- 83 µg/mL. The detected antimicrobial activity may involve complex mechanisms, like the inhibition of the synthesis of cell membranes, nucleic acids and proteins^38^. Moreover, the effects observed against both gram-positive and gram-negative bacteria may indicate the presence of not only antibiotic compounds but also metabolic toxins in plant fractions^39^.

**Table 2:** MIC (μg/mL, mean ± SD) of samples from *L. monopetalum* against human pathogenic bacteria.

Sample	MIC against Gram− bacteria		MIC against Gram + bacteria	
	*E. coli*	*P. aeruginosa*	*S. aureus*	*E. faecalis*
S1 (**1**)	250 ± 2.16	62.5 ± 0.54	62.5 ± 0.54	250 ± 2.16
F2	208.33 ± 0.9	208.33 ± 0.9	156.25 ± 1.35	133.20 ± 0.45
F3	520.83 ± 0.18	312.5 ± 2.70	416.66 ± 0.18	546.87 ± 6.2
F4	1041.66 ± 3.6	Inactive	625 ± 5.4	625 ± 5.4
F5	2500 ± 21.6	312.5 ± 2.70	2083.33 ± 7.21	2500 ± 21.6
F6	1041.66 ± 3.6	Inactive	364.58 ± 2.38	1250 ± 10.82
F7	78.12 ± 0.67	625 ± 5.41	2916.66 ± 19.09	833.5 ± 3.6
F8	62.5 ± 0.54	Inactive	156.25 ± 1.35	78.12 ± 0.36
Residue A’	156.25 ± 1.35	625 ± 5.41	156.25 ± 1.35	156.25 ± 1.35
Residue B	156.25 ± 1.35	156.25 ± 1.35	156.25 ± 1.35	156.25 ± 1.35
Residue C	78.12 ± 0.67	156.25 ± 1.35	78.12 ± 0.67	78.12 ± 0.67
Vancomycin	3.75 ± 0.00	1.875 ± 0.00	Inactive	60 ± 0.00
Imipenem	Inactive	Inactive	60 ± 0.00	Inactive

### Antiproliferative activity (MTT assay)

The cytotoxicity of *L. monopetalum* organs extracts and purified compounds was evaluated on Hela and A549 cell lines after incubation time of 48 h (Table 3). The IC_50_ values allowed us to note that the cytotoxic effects varied considerably with tested organs and the used cell lines. Organs extracts exhibited cytotoxicity against A549 lung epithelial carcinoma cell lines, with IC_50_ values ranging from 309 to 364 μg/mL. F8 exhibited the highest cytotoxicity against Hela and A549 cell lines (226 and 242.52 μg/mL, respectively). Leaves extract showed the lowest cytotoxic activity (IC_50_ = 514.8 μg/mL). In addition, the IC_50_ values allowed us to note that all tested fractions were more active towards A549 than Hela cell lines. Furthermore, Both F8 and maesopsin 6-*O*-glucoside exhibited much greater cytotoxicity than other fractions against the tested cell lines. F3 and F6 showed the similar effects against Hela and A549 cell lines, with IC50 around 315 µg/mL. The difference observed between cytotoxicity of crude leaves extract and the different fractions, may be explained by the difference of their chemical compositions, such as their polyphenol and flavonoid components, which can exhibit synergetic action (Table 3).

**Table 3:** Antiproliferative activity (MTT assay) of different samples from *L. monopetalum*. IC_50_ ± SD values are expressed in μg/mL.

	Hela cell line	A549 cell line
S1	309.15 ± 7.51	283.59 ± 12.26
F2	544.24 ± 10.22	376.95 ± 13.41
F3	416.11 ± 6.71	314.98 ± 10.25
F4	876.97 ± 16.33	395.79 ± 14.72
F5	409.20 ± 11.46	303.01 ± 12.14
F6	415.86 ± 13.34	314.67 ± 11.18
F7	427.20 ± 10.41	329.18 ± 8.44
F8	226.00 ± 8.83	242.52 ± 15.63
Residue A’	514.80 ± 18.36	309.99 ± 17.44
Residue B	201.94 ± 7.33	321.49 ± 11.66
Residue C	441.48 ± 21.42	364.85 ± 14.58

### Chemical composition of volatile fractions

The analyzation of volatile fractions with GC–FID and GC–MS, was reported in Table 4. Odorous properties of methanolic extracts of *L. monopetalum* were due to the presence of volatile compounds, which were obtainable by hydrodistillation, using a Clevenger-type apparatus. Consequently, Six constituents were identified in leaves and roots extract, however, five compounds where identified in stem methanolic extracts. The most abundant constituent from methanolic extracts was camphor. It was naturally precipitated by hydro distillation of *L. monopetalum* roots (200 mg), and it was more predominant in roots than stems and leaves. However, the volatile fraction of *L. monopetalum* stems (F2) and roots (F3), were characterized by the presence of cyclopent-2-enone < 5-methylene-2,3,4,4-tetramethyl- > , with 1.39% and 3.54% respectively. In addition of odecanoic acid (0.66%). While Thujone < cis- > , was present only in *L. monopetalum* roots extract (2.88%). Hammami et al., 2011, analyzed the essential oil of *Limoniastrum gyonianum* leaves, flowers, seeds and roots^35^. The results indicated the absence of all the chemical compounds presented in Table 3 and identified in the volatile fractions of *L. monopetalum* (L.).

**Table 4:** Chemical composition (%) of the volatile fractions of *L. monopetalum* (L.) Boiss. leaves, stems and roots methanolic extracts.

Compounds		CIR	TIR	F_1_	F_2_	F_3_
Fenchone		1087	1086		0.11	0.36
Thujone < cis- >		1098	1095			2.88
NI		1103		0.33	1.32	7.27
NI		1117		0.03	0.17	0.72
Camphor		1143	1141	99.48	96.45	84.58
Karahanaenone		1156	1154		0.22	0.16
NI		1159			0.24	0.34
Isoborneol		1164	1155		0.09	0.14
Cyclopent-2-enone < 5-methylene-2,3,4,4-tetramethyl- >		1191	1183	0.01	1.39	3.54
Isophorone < 4-methylene- >		1215	1216	0.15		
Indole		1291	1290	0.01		

*CIR* calculated index retention, *TIR* theoretical index retention, *NI* not identified, *F*_*1*_ methanolic leaves extract, *F*_*2*_ methanolic stems extract, *F*_*3*_ methanolic root extract.

The interesting biological activities observed with *L. monopetalum* methanolic organ extracts, can be explained by the presence of phenolic components, which can exhibit antimicrobial and antitumoral activities^36,37^. In fact, the antibacterial activities can also be attributed to the presence of camphor as a major component (from 84.58 to 99.48%). However, some volatile compounds, such as β-thujone, a-thujone and camphor, have been reported to exhibit a wide range of anti-fungal and antibacterial activities^38,39^.

### In silico study

The virulence of *S. aureus* strain, is administered by the accessory gene regulator (agr) quorum sensing (QS) system. When the agr system is triggered, the pathogen releases an extracellular peptide signal (Agr), by which the bacteria become able to sense the local density of *S. aureus* population^18^. When the cell bacteria density reaches the quorum, the peptide Agr induce the expression of several extracellular toxins^19^, at the same time, repressed the cell surface adhesins to block cells colonization^19^. As discussed in the Introduction, the inhibition of the Agr protein secreted by *Staphylococcus aureus* may be a mechanism to reduce the severity of the infections caused by the bacteria. In this context, we considered it to be interesting to investigate, through the in silico study, the inhibitory potential of (2*S*)-maesopsin-6-*O*-glucoside (**1**), which showed good activity against *S. aureus* (Table 2). A few years ago, a hydrophobic cleft was identified in the LytTR domain of AgrA as a locus for small molecule interactions that inhibit DNA binding, making it a potential target for antimicrobial development. Moreover, the crystal structure of the apo AgrA LytTR domain was determined^41^.

### Molecular docking

**Figure 2:**
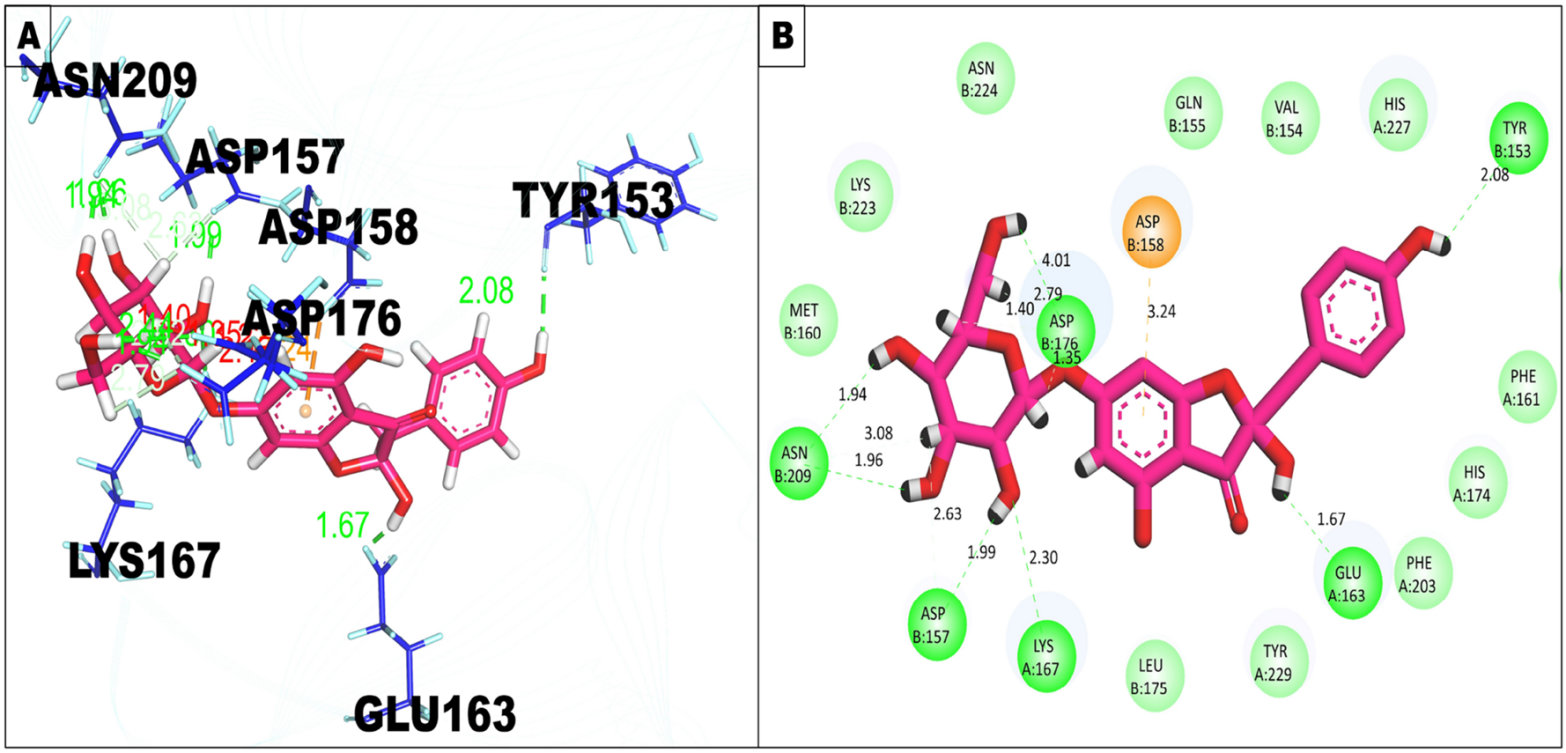
Interaction of (2*S*)-maesopsin 6-*O*-glucoside with the binding cleft of 4g4k, shown in 3D and 2D representation.

Molecular docking was performed, using AutoDock Tools 1.5.4, to evaluate the inhibitory potential of (2*S*)-maesopsin 6-*O*-glucoside against the accessory gene regulator protein A (AgrA) of *Staphylococcus aureus* (Fig. 2).

The results indicate that the (2*S*)-maesopsin 6-*O*-glucoside binds to the protein 4g4k with a docking score of −7.2 kcal/mol, which suggests a stronger interaction between the compound and the target protein (4G4K). Remarkably, the docking score was better than the polyhydroxy anthraquinone ω-hydroxyemodin (ΔG = −5.95 kcal/mol), a polyhydroxyanthraquinone isolated from solid-phase cultures of *Penicillium restrictum*^42^, as well as by leotiomycene A (ΔG = −5.5 kcal/mol), a prenylated diresorcinol isolated from a freshwater fungus (*Helotiales* sp.)^43^. In addition, Daly et al.^40^ and Paguigan et al.^39^ indicate that the fungi metabolites, polyhydroxy anthraquinone ω-hydroxyemodin and leotiomycene, exhibit in vitro inhibition of *Staphylococcus aureus* Quorum Sensing by direct binding to AgrA, at not cytotoxic concentrations to eukaryotic cells. Interestingly, docking studies indicated that the putative mode of binding of (2*S*)-maesopsin 6-*O*-glucoside bind to the amino acids found in the binding site with three hydrogen bonds: Glu 144, Thr 142, Glu 188, along with two pi-alkyl bonds: Leu 192, Leu 189. This putative mode of binding is predicted to be the same as both fungi metabolites, polyhydroxyanthraquinone ω-hydroxyemodin and Leotiomycene, as described by Paguigan et al.^41^.

### Molecular dynamic simulation analysis

**Figure 3:**
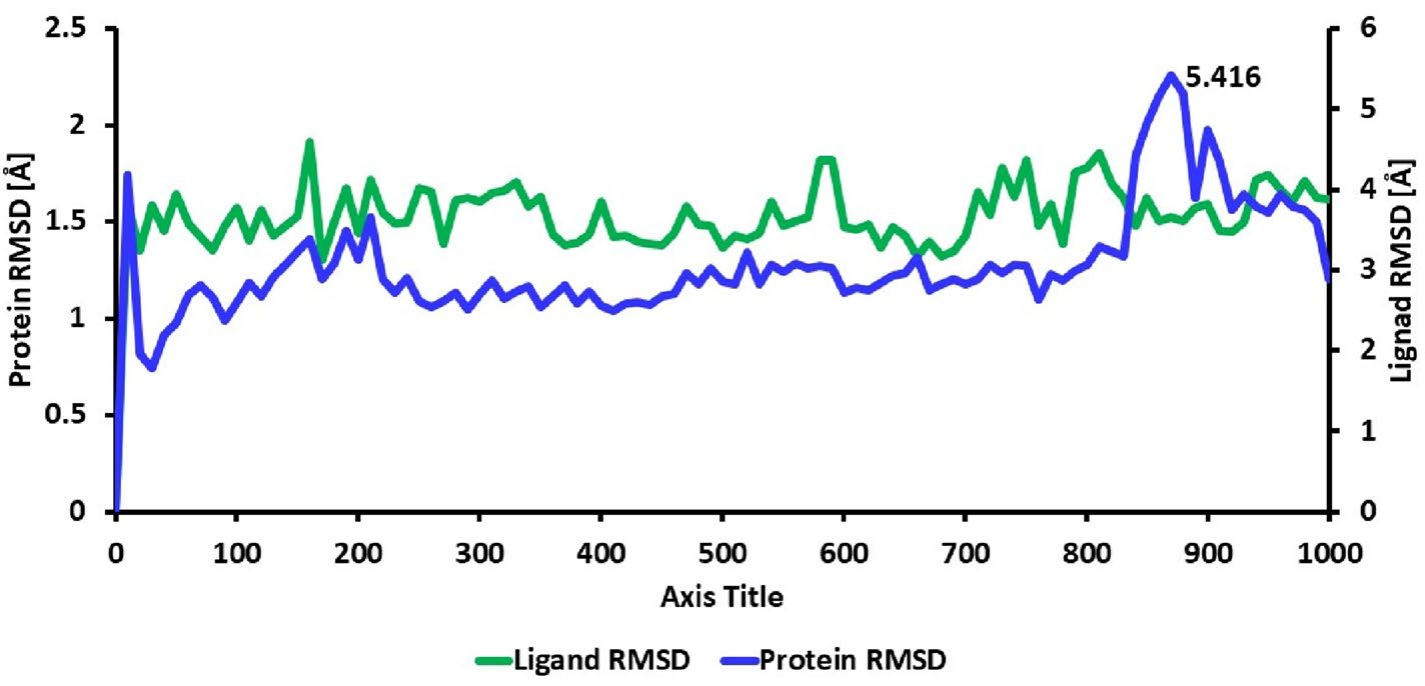
RMSD plot of (2*S*)-maesopsin 6-*O*-glucoside**-**4g4k complex system during 1000 ns.

**Figure 4:**
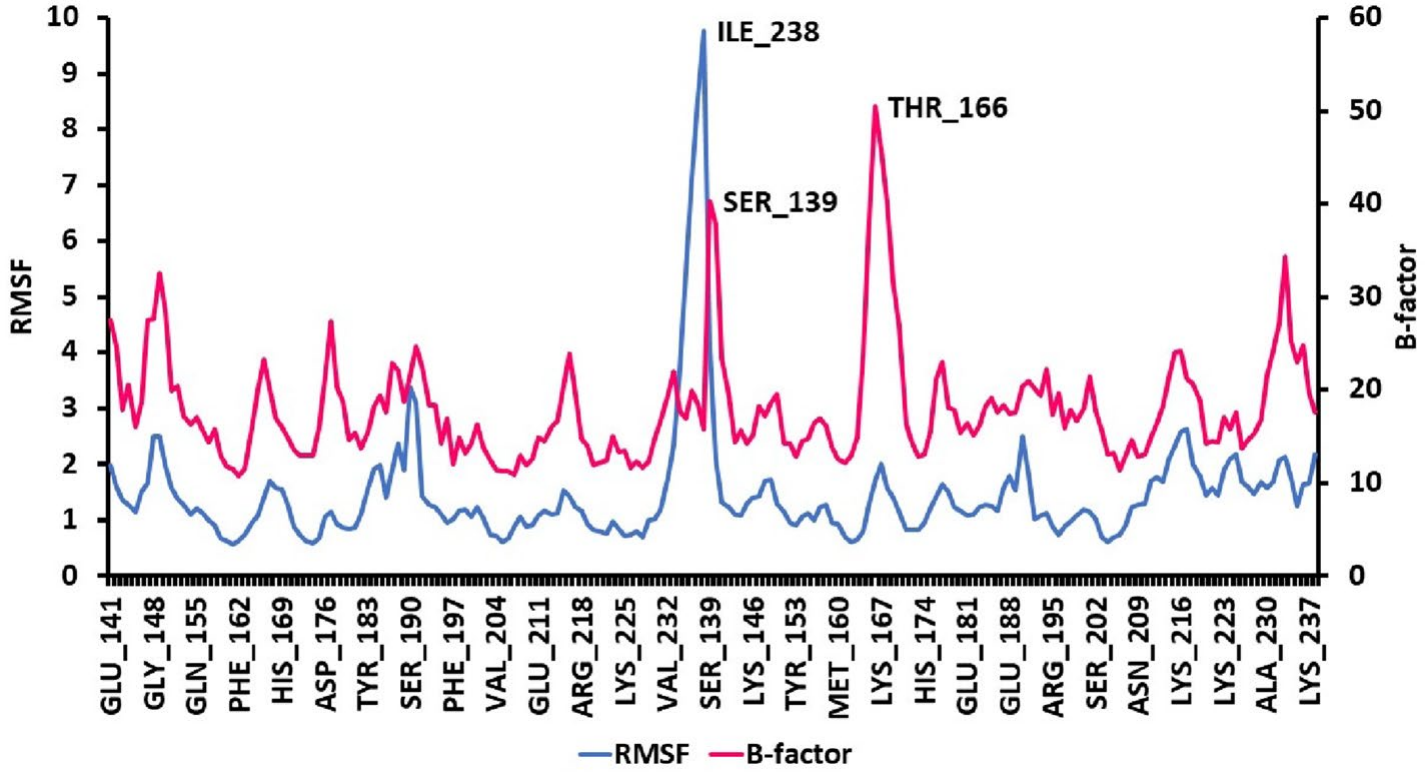
Protein RMSF of (2*S*)-maesopsin 6-*O*-glucoside-4g4k complex (1000 ns).

Molecular dynamics simulations were performed to evaluate the stability of the complex of (2*S*)-maesopsin 6-*O*-glucoside-4g4k. The RMSD value was used to assess the stability of the simulation system and the conformational perturbations of the protein backbone caused by simulation. Figure 3 shows the RMSD plots of the complex having the fluctuation during 1000 ns time interval. The complexes, (2*S*)-maesopsin 6-O-glucoside**-**4g4k, in Fig. 3 depict lesser variation during the entire MD simulation. In fact, the RMSD values of protein and ligand was calculated as 0–2.5 Å and 0–5 Å respectively. The sudden change was noticed at 870 ns with the 5.4 Å in protein RMSD. Furthermore, RMSF analysis was carried out to comprehend the residue-wise variation, along the protein chain, of the docked complexes. The docked complex was used to produce RMSF behavior, and a plot was created utilizing the RMSF, B factor, and interactions Fig. 4**.** The Root Mean Square Fluctuation (RMSF) property shows the average deviation of the receptor relative to the reference position. From Fig. 4**,** it is evident that the RMSF value of the protein backbone residues remained under 4 Å range except Ile 238 residues (9.75 Å). Various parameters for the ligand were estimated to get insight into the conformational strain the ligand suffers to maintain the protein-bound complex. The average RMSF value was acquired by the (2*S*)-maesopsin 6-*O*-glucoside**-**4g4k complex which possessed the 1.50 Å, while β-factors showed higher fluctuations which ranged between 15 and 35 Å.

**Figure 5:**
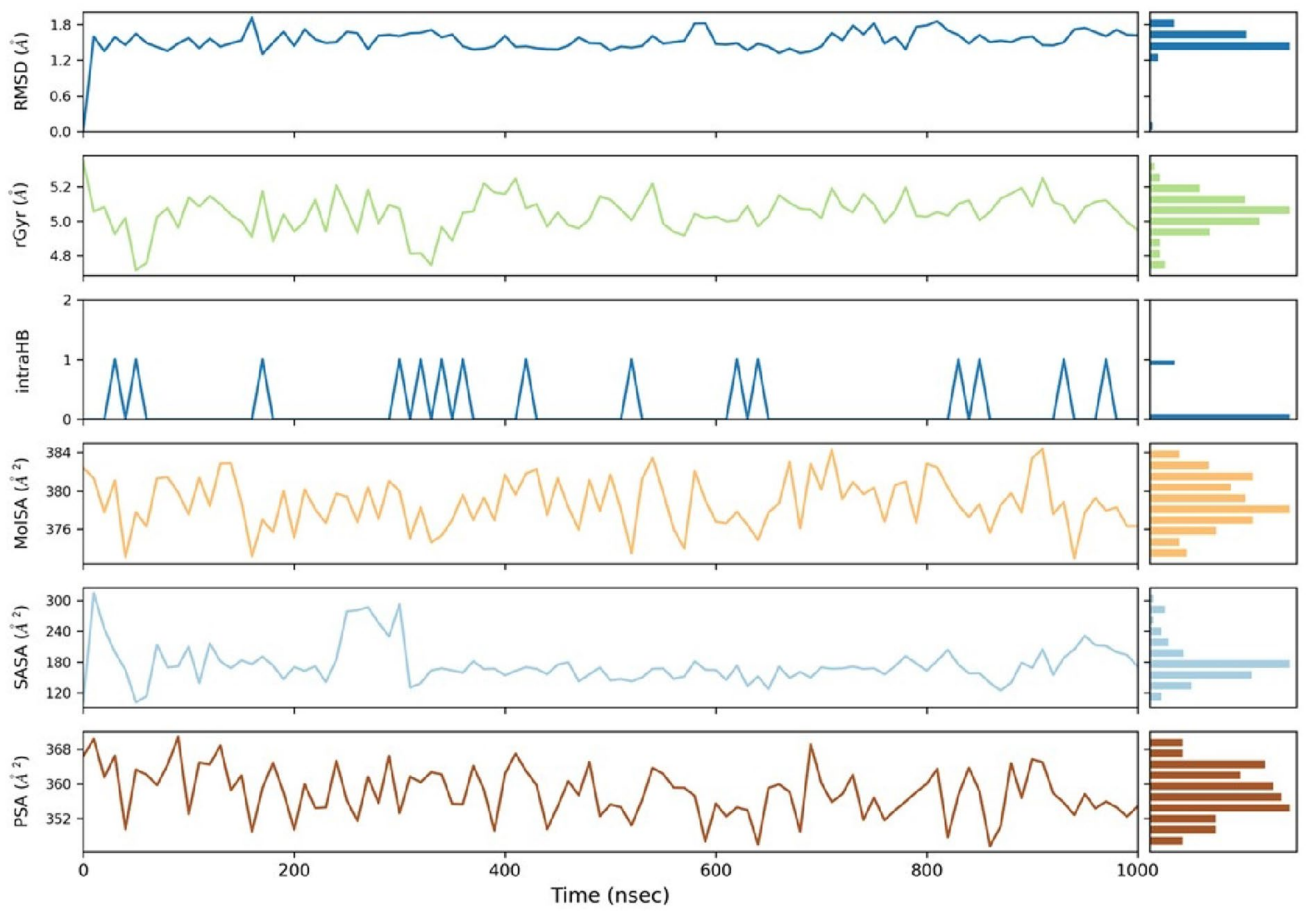
Ligand properties of (2*S*)-maesopsin 6-*O*-glucoside-4g4k complex.

**Figure 6:**
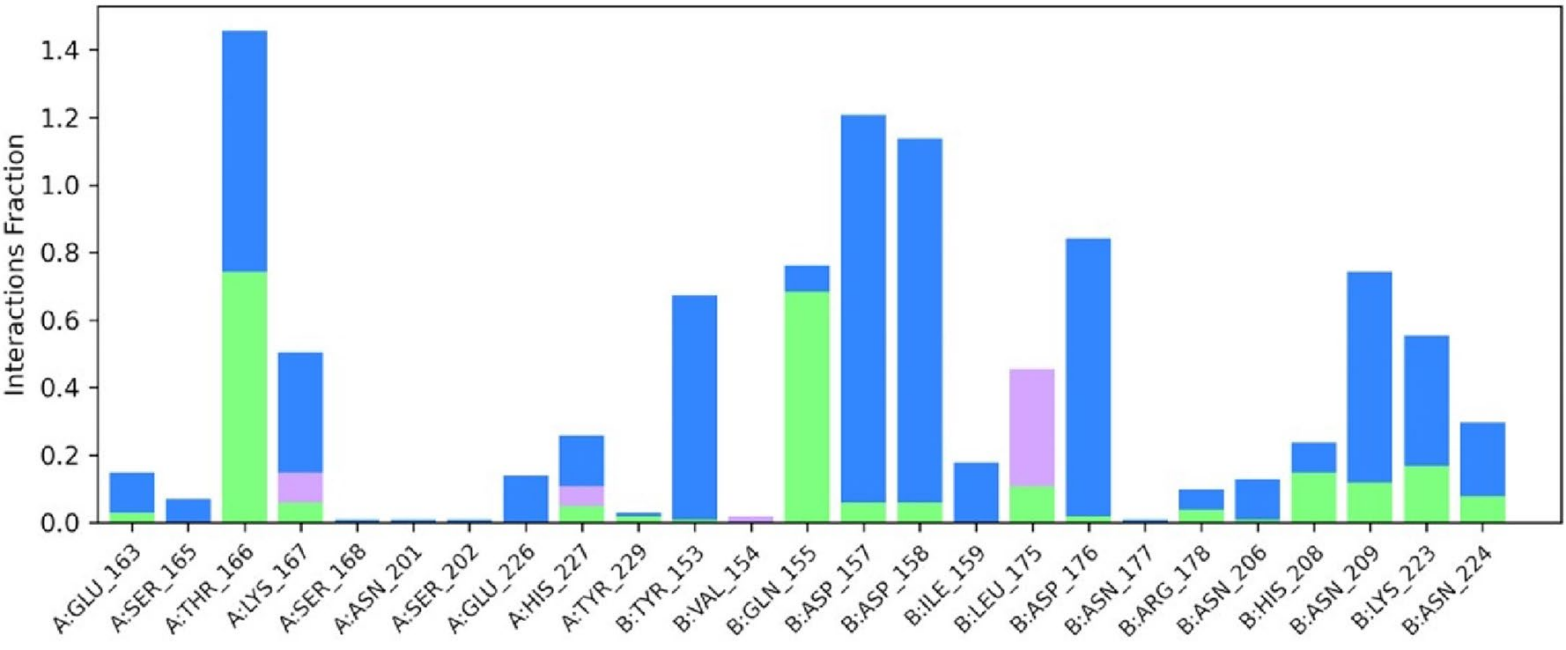
A histogram displaying the different types of molecular interactions involved between (2*S*)-maesopsin 6-*O*-glucoside within the pockets of 4g4k receptor at specific sites.

Figure 5 represents the ligand properties of the complex, including RMSD, rGyr, intraHB, MolSA, SASA and PSA. The RMSD value remained under 2 Å, while the radius of gyration rGyr were measured slightly higher to RMSD values. The other values of intra-H-bonds (intraHB), molecular surface area (MolSA), solvent accessible surface area (SASA) and polar surface area (PSA), were also notified with less fluctuation. The values for RMSD (Cα atoms and ligand fit on protein) as well as RMSF (Cα atoms) were consistent with the stability of both the complexes formed during the entire time of 1000 ns MD simulation. Figure 6 describes the protein–ligand interaction profile of crucial interacting amino acids, with the color denotation viz: hydrogen bond (green), hydrophobic contacts (purple), ionic bond and water-bridge (blue).

From the result in Fig. 6**,** hydrogen and water bridges bonds were identified with higher numbers during MD simulation event. In fact, hydrogen bonds play a central role in the maintenance of the conformational integrity of ligands at the active protein residue bonding sites. As a result, more hydrogen bonds and water bridges between the protein and ligand improve the stability of (2*S*)-maesopsin 6-*O*-glucoside**-**4g4k complex. Thr166 and Gln 155 accumulated higher numbers of hydrogen bonds among 17 hydrogen bonds, Leu175 developed the most numbers of hydrophobic interactions. The free energy of binding of the complexes were calculated using MM/GBSA method of Maestro prime module. To estimate the ligand-binding affinities in the protein system, free energy calculations are frequently utilized and highly recognized (Table 5).

**Table 5:** MM/GBSA profiles of the complex, maesopsin 6-*O*-glucoside with the accessory gene regulator protein A.

Time (ns)	ΔG bind (kcal/mol)	ΔG bind Coulomb (kcal/mol)	ΔG bind covalent (kcal/mol)	ΔG bind Vander (kcal/mol)	ΔG bind H Bond (kcal/mol)	ΔG bind Lipophilic (kcal/mol)	ΔG binding SolvGB (kcal/mol)
0	−63.52	−54.80	6.83	−49.24	−4.38	−10.23	49.43
100	−47.70	−19.57	2.32	−47.18	−2.03	−11.43	30.90
200	−37.98	−10.90	4.86	−46.99	−1.78	−11.44	28.61
300	−42.63	−24.15	2.87	−36.48	−1.94	−9.67	27.02
400	−40.21	−29.29	7.42	−43.92	−3.95	−8.43	38.18
500	−42.73	−16.63	4.71	−46.00	−2.67	−11.43	29.30
600	−43.27	−14.24	4.23	−46.73	−2.24	−12.31	28.33
700	−47.61	−23.88	3.93	−42.46	−3.49	−11.88	30.57
800	−54.85	−22.52	6.15	−49.39	−3.20	−12.10	28.10
900	−42.84	−24.54	2.67	−43.48	−2.66	−10.84	37.18
1000	−33.82	−23.21	1.77	−40.06	−2.08	−9.96	40.58
Avg	−45.20	−23.98	4.34	−44.72	−2.77	−10.88	33.47

*ΔG bind* free energy of binding, *ΔG bind Coulomb* Coulomb energy, *ΔG bind covalent* covalent energy (internal energy), *ΔG bind Vander* van der Waals energy, *ΔG bind H bond* hydrogen bonding energy, *ΔG bind lipophilic* hydrophobic energy (non-polar contribution estimated by solvent accessible surface area).

The *ΔG* (MM/GBSA) free energy calculated was −45.20 kcal/mol for the complex (2*S*)-maesopsin 6-*O*-glucoside-*Staphylococcus aureus* accessory gene regulator protein A (4g4k). While coulomb energy was −23.98 kcal/mol. The covalent bind energy remained 4.34 kcal/mol. However, the van Der Waals interactions were notified at −44.72 kcal/mol and hydrogen bonding energy was found with −2.77 kcal/mol. The lipophilicity and solvation energy acquired the energy values of −10.88 kcal/mol and 33.47 kcal/mol, respectively. The binding energy value was negative, suggesting that the compounds had favorable interactions with the protein 4g4k. Considering the obtained results, we hypothesized that maesopsin 6-O-glucoside would be a potent inhibitor of the AgrA component of the agr quorum sensing system, however, in vitro study should be undertaken in order to confirm this finding.

## Conclusion

In this inaugural study of *L. monopetalum*, the chemical composition of volatile fractions isolated from different parts of the plant was determined. Moreover, important biological activities, such as the antibacterial and antiproliferative effects of different extracts, chromatographic fractions, and isolated compounds, have been evaluated by in vitro assays. Especially promising are the antibacterial properties determined against gram + and gram − bacteria. They suggest the presence of bioactive compounds which shall stimulate further studies aimed at their bio-guided isolation.

In silico studies of the (2*S*)-stereoisomer indicated that the isolated metabolite maesopsin-6-*O*-glucoside has the potential to reduce the virulence of *Staphylococcus aureus* strains through the inhibition of the AgrA protein, which is involved in the regulation of the Quorum Sensing system. Interestingly, the cytotoxicity of this flavonoid (Table 3) was significantly lower than the MIC against *S. aureus* (Table 2). However, to complete the biological characterization of maesopsin-6-*O*-glucoside, the in-silico investigation must be extended to both stereoisomers constituting this flavonoid; moreover, other specific in vitro and in vivo experiments are needed to confirm the therapeutic potential. As the final remark, the good antibacterial properties observed for *L. monopetalum* extracts give scientific support to the traditional use of this halophyte against infectious diseases and parasites that cause painful and bloody diarrhea^12^.

### Supplementary Information

Supplementary Information.

## Data Availability

The datasets used and/or analysed during the current study available from the corresponding author on reasonable request.
