# Author Correction: Phytochemical analysis, biological activities of methanolic extracts and an isolated flavonoid from Tunisian *Limoniastrum monopetalum* (L.) Boiss: an in vitro and in silico investigations

**DOI:** 10.1038/s41598-024-53821-7

**Published:** 2024-02-08

**Authors:** Amel Bouzidi, Ahmed Azizi, Omar Messaoudi, Kirouani Abderrezzak, Giovanni Vidari, Ahmed Noureddine Hellal, Chirag N. Patel

**Affiliations:** 1 BTP Laboratory, Department of Biology, Faculty of Sciences, University of Medea, Médéa, Algeria; 2 Faculty of Technology, University Amar Telidji, Highway Ghardaia, Post Box G37 (M’kam), 03000 Laghouat, Algeria; 3 Laboratory of Applied Microbiology in Food, Biomedical and Environment, Abou Bekr Belkaïd University, 13000 Tlemcen, Algeria; 4 Department of Biology, Faculty of Science, University of Amar Telidji, 03000 Laghouat, Algeria; 5 Department of Medical Analysis, Faculty of Applied Science, Ishk International University, Erbil, 44001 Iraq; 6 Laboratory of Bioressources, Biology Integrative and Valorization, Higher Institute of Biotechnology of Monastir, University of Monastir, Monastir, Tunisia; 7 Department of Botany, Bioinformatics and Climate Change Impacts Management, School of Science, Gujarat University, Ahmedabad, Gujarat 380009 India; 8 Biotechnology Research Center, Technology Innovation Institute, 9639 Abu Dhabi, United Arab Emirates

Correction to: *Scientific Reports* 10.1038/s41598-023-46457-6, published online 06 November 2023

The original version of this Article contained errors in the spelling of the authors Omar Messaoudi and Giovanni Vidari which were incorrectly given as Omar Messoudi and Vidari Giovanni.

The original Article has been corrected.

