# Supplementary material for: Phytochemical analysis, biological activities of methanolic extracts and an isolated flavonoid from Tunisian Limoniastrum monopetalum (L.) Boiss: an in vitro and in silico investigations
Source: Sci Rep. 2023 Nov 6;13:19144. doi: 10.1038/s41598-023-46457-6 (PMC10628221; doi:10.1038/s41598-023-46457-6)
Supplement: Supplementary file 1 — Supplementary Information. [file 41598_2023_46457_MOESM1_ESM.zip › Supplementary files/4G4K_10765955.docx]

HEADER DNA BINDING PROTEIN 16-JUL-12 4G4K

REMARK 4 4G4K COMPLIES WITH FORMAT V. 3.30,

REMARK 888

REMARK 888 WRITTEN BY MAESTRO (A PRODUCT OF SCHRODINGER, LLC)

TITLE STRUCTURE OF THE STAPHYLOCOCCUS AUREUS AGRA LYTTR DOMAIN

EXPDTA X-RAY DIFFRACTION

REMARK 2 RESOLUTION. 1.52 ANGSTROMS

REMARK 200 TEMPERATURE (KELVIN) : 100.00

REMARK 200 PH : 8.00

REMARK 350 BIOMOLECULE: 1

REMARK 350 APPLY THE FOLLOWING TO CHAINS: A

REMARK 350 BIOMT1 1 1.000000 0.000000 0.000000 0.000000

REMARK 350 BIOMT2 1 0.000000 1.000000 0.000000 0.000000

REMARK 350 BIOMT3 1 0.000000 0.000000 1.000000 0.000000

REMARK 350 BIOMOLECULE: 2

REMARK 350 APPLY THE FOLLOWING TO CHAINS: B

REMARK 350 BIOMT1 1 1.000000 0.000000 0.000000 0.000000

REMARK 350 BIOMT2 1 0.000000 1.000000 0.000000 0.000000

REMARK 350 BIOMT3 1 0.000000 0.000000 1.000000 0.000000

CRYST1 41.414 45.894 112.138 90.00 90.00 90.00 P 21 21 21 8

MODEL 1

ATOM 1 CH3 ACE A 140 38.811 56.085 43.369 1.00 0.00 C

ATOM 2 C ACE A 140 38.508 54.587 43.310 1.00 0.00 C

ATOM 3 O ACE A 140 38.556 53.998 42.233 1.00 0.00 O

ATOM 4 1H ACE A 140 39.101 56.461 42.388 1.00 0.00 H

ATOM 5 2H ACE A 140 37.929 56.634 43.699 1.00 0.00 H

ATOM 6 3H ACE A 140 39.624 56.280 44.068 1.00 0.00 H

ATOM 7 N GLU A 141 38.182 53.898 44.460 1.00 33.10 N

ATOM 8 CA GLU A 141 37.894 52.467 44.397 1.00 26.23 C

ATOM 9 C GLU A 141 36.711 52.178 43.486 1.00 25.29 C

ATOM 10 O GLU A 141 35.864 51.331 43.783 1.00 25.15 O

ATOM 11 CB GLU A 141 37.670 51.898 45.797 1.00 29.86 C

ATOM 12 CG GLU A 141 38.941 51.943 46.631 1.00 28.01 C

ATOM 13 CD GLU A 141 38.767 51.420 48.040 1.00 29.70 C

ATOM 14 OE1 GLU A 141 39.758 51.473 48.800 1.00 29.81 O

ATOM 15 OE2 GLU A 141 37.657 50.958 48.387 1.00 29.72 O1-

ATOM 16 H1 GLU A 141 38.147 54.368 45.353 1.00 0.00 H

ATOM 17 HA GLU A 141 38.755 51.957 43.959 1.00 0.00 H

ATOM 18 HB3 GLU A 141 37.344 50.859 45.739 1.00 0.00 H

ATOM 19 HB2 GLU A 141 36.868 52.446 46.295 1.00 0.00 H

ATOM 20 HG3 GLU A 141 39.316 52.964 46.704 1.00 0.00 H

ATOM 21 HG2 GLU A 141 39.722 51.356 46.147 1.00 0.00 H

ATOM 22 N THR A 142 36.678 52.885 42.363 1.00 26.09 N

ATOM 23 CA THR A 142 35.604 52.757 41.389 1.00 22.90 C

ATOM 24 C THR A 142 35.992 51.789 40.281 1.00 21.99 C

ATOM 25 O THR A 142 37.108 51.847 39.767 1.00 27.53 O

ATOM 26 CB THR A 142 35.289 54.125 40.766 1.00 24.70 C

ATOM 27 OG1 THR A 142 34.861 55.019 41.795 1.00 28.77 O

ATOM 28 CG2 THR A 142 34.192 54.007 39.720 1.00 21.51 C

ATOM 29 H THR A 142 37.465 53.501 42.156 1.00 0.00 H

ATOM 30 HA THR A 142 34.696 52.401 41.876 1.00 0.00 H

ATOM 31 HB THR A 142 36.182 54.552 40.307 1.00 0.00 H

ATOM 32 HG1 THR A 142 34.107 54.647 42.234 1.00 0.00 H

ATOM 33 HG21 THR A 142 33.867 54.996 39.394 1.00 0.00 H

ATOM 34 HG22 THR A 142 34.516 53.484 38.820 1.00 0.00 H

ATOM 35 HG23 THR A 142 33.314 53.492 40.114 1.00 0.00 H

ATOM 36 N ILE A 143 35.074 50.896 39.913 1.00 18.36 N

ATOM 37 CA ILE A 143 35.311 49.984 38.807 1.00 18.05 C

ATOM 38 C ILE A 143 34.228 50.162 37.758 1.00 17.89 C

ATOM 39 O ILE A 143 33.047 50.228 38.075 1.00 17.17 O

ATOM 40 CB ILE A 143 35.382 48.497 39.254 1.00 19.79 C

ATOM 41 CG1 ILE A 143 35.597 47.575 38.055 1.00 22.17 C

ATOM 42 CG2 ILE A 143 34.127 48.092 39.985 1.00 24.59 C

ATOM 43 CD1 ILE A 143 35.805 46.133 38.441 1.00 26.31 C

ATOM 44 H ILE A 143 34.174 50.870 40.379 1.00 0.00 H

ATOM 45 HA ILE A 143 36.260 50.196 38.310 1.00 0.00 H

ATOM 46 HB ILE A 143 36.226 48.387 39.933 1.00 0.00 H

ATOM 47 HG13 ILE A 143 36.473 47.911 37.499 1.00 0.00 H

ATOM 48 HG12 ILE A 143 34.760 47.601 37.358 1.00 0.00 H

ATOM 49 HG21 ILE A 143 34.266 47.109 40.422 1.00 0.00 H

ATOM 50 HG22 ILE A 143 33.906 48.744 40.824 1.00 0.00 H

ATOM 51 HG23 ILE A 143 33.256 48.028 39.335 1.00 0.00 H

ATOM 52 HD11 ILE A 143 36.143 45.562 37.578 1.00 0.00 H

ATOM 53 HD12 ILE A 143 36.551 46.027 39.228 1.00 0.00 H

ATOM 54 HD13 ILE A 143 34.881 45.669 38.785 1.00 0.00 H

ATOM 55 N GLU A 144 34.644 50.264 36.502 1.00 20.17 N

ATOM 56 CA GLU A 144 33.693 50.360 35.412 1.00 19.15 C

ATOM 57 C GLU A 144 33.317 48.955 34.955 1.00 20.96 C

ATOM 58 O GLU A 144 34.189 48.163 34.592 1.00 22.12 O

ATOM 59 CB GLU A 144 34.284 51.156 34.248 1.00 20.80 C

ATOM 60 CG GLU A 144 33.345 51.245 33.053 1.00 20.77 C

ATOM 61 CD GLU A 144 34.008 51.836 31.827 1.00 28.52 C

ATOM 62 OE1 GLU A 144 33.362 51.840 30.760 1.00 28.52 O

ATOM 63 OE2 GLU A 144 35.172 52.281 31.931 1.00 30.48 O1-

ATOM 64 H GLU A 144 35.628 50.210 36.284 1.00 0.00 H

ATOM 65 HA GLU A 144 32.792 50.892 35.725 1.00 0.00 H

ATOM 66 HB3 GLU A 144 35.222 50.685 33.947 1.00 0.00 H

ATOM 67 HB2 GLU A 144 34.551 52.156 34.591 1.00 0.00 H

ATOM 68 HG3 GLU A 144 32.490 51.862 33.320 1.00 0.00 H

ATOM 69 HG2 GLU A 144 32.957 50.275 32.743 1.00 0.00 H

ATOM 70 N LEU A 145 32.025 48.637 34.998 1.00 13.50 N

ATOM 71 CA LEU A 145 31.554 47.362 34.485 1.00 15.36 C

ATOM 72 C LEU A 145 30.910 47.622 33.128 1.00 16.67 C

ATOM 73 O LEU A 145 29.783 48.115 33.037 1.00 18.45 O

ATOM 74 CB LEU A 145 30.541 46.710 35.420 1.00 15.37 C

ATOM 75 CG LEU A 145 30.932 46.499 36.884 1.00 16.21 C

ATOM 76 CD1 LEU A 145 29.739 45.943 37.670 1.00 15.84 C

ATOM 77 CD2 LEU A 145 32.165 45.588 37.006 1.00 16.97 C

ATOM 78 H LEU A 145 31.341 49.306 35.335 1.00 0.00 H

ATOM 79 HA LEU A 145 32.359 46.638 34.348 1.00 0.00 H

ATOM 80 HB3 LEU A 145 30.199 45.767 34.998 1.00 0.00 H

ATOM 81 HB2 LEU A 145 29.710 47.365 35.405 1.00 0.00 H

ATOM 82 HG LEU A 145 31.194 47.462 37.324 1.00 0.00 H

ATOM 83 HD11 LEU A 145 30.060 45.573 38.645 1.00 0.00 H

ATOM 84 HD12 LEU A 145 28.986 46.700 37.874 1.00 0.00 H

ATOM 85 HD13 LEU A 145 29.262 45.111 37.150 1.00 0.00 H

ATOM 86 HD21 LEU A 145 32.448 45.457 38.051 1.00 0.00 H

ATOM 87 HD22 LEU A 145 31.968 44.600 36.592 1.00 0.00 H

ATOM 88 HD23 LEU A 145 33.031 46.003 36.491 1.00 0.00 H

ATOM 89 N LYS A 146 31.635 47.261 32.079 1.00 17.47 N

ATOM 90 CA LYS A 146 31.195 47.504 30.715 1.00 19.89 C

ATOM 91 C LYS A 146 30.107 46.507 30.328 1.00 19.66 C

ATOM 92 O LYS A 146 30.348 45.306 30.293 1.00 17.43 O

ATOM 93 CB LYS A 146 32.397 47.406 29.777 1.00 19.76 C

ATOM 94 CG LYS A 146 33.481 48.441 30.079 1.00 30.18 C

ATOM 95 CD LYS A 146 34.631 48.376 29.086 1.00 45.06 C

ATOM 96 CE LYS A 146 35.580 49.554 29.266 1.00 41.55 C

ATOM 97 NZ LYS A 146 36.753 49.499 28.346 1.00 42.91 N1+

ATOM 98 H LYS A 146 32.548 46.853 32.221 1.00 0.00 H

ATOM 99 HA LYS A 146 30.778 48.504 30.638 1.00 0.00 H

ATOM 100 HB3 LYS A 146 32.057 47.537 28.748 1.00 0.00 H

ATOM 101 HB2 LYS A 146 32.832 46.406 29.828 1.00 0.00 H

ATOM 102 HG3 LYS A 146 33.866 48.323 31.093 1.00 0.00 H

ATOM 103 HG2 LYS A 146 33.026 49.428 30.031 1.00 0.00 H

ATOM 104 HD3 LYS A 146 34.241 48.382 28.067 1.00 0.00 H

ATOM 105 HD2 LYS A 146 35.169 47.435 29.211 1.00 0.00 H

ATOM 106 HE3 LYS A 146 35.949 49.588 30.292 1.00 0.00 H

ATOM 107 HE2 LYS A 146 35.053 50.492 29.089 1.00 0.00 H

ATOM 108 HZ1 LYS A 146 37.280 48.655 28.520 1.00 0.00 H

ATOM 109 HZ2 LYS A 146 36.432 49.508 27.388 1.00 0.00 H

ATOM 110 HZ3 LYS A 146 37.342 50.303 28.511 1.00 0.00 H

ATOM 111 N ARG A 147 28.901 46.995 30.053 1.00 23.40 N

ATOM 112 CA ARG A 147 27.770 46.099 29.810 1.00 24.43 C

ATOM 113 C ARG A 147 27.475 45.863 28.339 1.00 28.85 C

ATOM 114 O ARG A 147 26.810 44.884 27.978 1.00 33.41 O

ATOM 115 CB ARG A 147 26.511 46.609 30.509 1.00 26.99 C

ATOM 116 CG ARG A 147 26.554 46.411 32.007 1.00 23.74 C

ATOM 117 CD ARG A 147 25.332 46.972 32.697 1.00 30.23 C

ATOM 118 NE ARG A 147 24.085 46.572 32.062 1.00 31.67 N

ATOM 119 CZ ARG A 147 23.551 45.360 32.131 1.00 35.57 C

ATOM 120 NH1 ARG A 147 24.161 44.390 32.802 1.00 39.21 N

ATOM 121 NH2 ARG A 147 22.405 45.114 31.511 1.00 39.29 N1+

ATOM 122 H ARG A 147 28.733 47.993 30.074 1.00 0.00 H

ATOM 123 HA ARG A 147 27.957 45.106 30.223 1.00 0.00 H

ATOM 124 HB3 ARG A 147 25.652 46.061 30.118 1.00 0.00 H

ATOM 125 HB2 ARG A 147 26.321 47.654 30.273 1.00 0.00 H

ATOM 126 HG3 ARG A 147 27.436 46.904 32.414 1.00 0.00 H

ATOM 127 HG2 ARG A 147 26.666 45.369 32.245 1.00 0.00 H

ATOM 128 HD3 ARG A 147 25.376 48.061 32.655 1.00 0.00 H

ATOM 129 HD2 ARG A 147 25.322 46.721 33.754 1.00 0.00 H

ATOM 130 HE ARG A 147 23.619 47.281 31.513 1.00 0.00 H

ATOM 131 HH12 ARG A 147 23.757 43.466 32.845 1.00 0.00 H

ATOM 132 HH11 ARG A 147 25.066 44.554 33.220 1.00 0.00 H

ATOM 133 HH22 ARG A 147 21.992 44.193 31.544 1.00 0.00 H

ATOM 134 HH21 ARG A 147 21.935 45.845 30.996 1.00 0.00 H

ATOM 135 N GLY A 148 27.976 46.761 27.502 1.00 23.61 N

ATOM 136 CA GLY A 148 27.685 46.752 26.082 1.00 25.87 C

ATOM 137 C GLY A 148 27.619 48.195 25.632 1.00 24.62 C

ATOM 138 O GLY A 148 28.646 48.859 25.498 1.00 36.50 O

ATOM 139 H GLY A 148 28.517 47.532 27.870 1.00 0.00 H

ATOM 140 HA3 GLY A 148 26.751 46.239 25.843 1.00 0.00 H

ATOM 141 HA2 GLY A 148 28.487 46.238 25.550 1.00 0.00 H

ATOM 142 N SER A 149 26.405 48.686 25.417 1.00 29.97 N

ATOM 143 CA SER A 149 26.200 50.073 25.019 1.00 32.16 C

ATOM 144 C SER A 149 26.455 51.018 26.188 1.00 34.88 C

ATOM 145 O SER A 149 26.863 52.164 25.993 1.00 32.96 O

ATOM 146 CB SER A 149 24.776 50.265 24.504 1.00 34.94 C

ATOM 147 OG SER A 149 23.839 50.041 25.545 1.00 45.66 O

ATOM 148 H SER A 149 25.591 48.102 25.544 1.00 0.00 H

ATOM 149 HA SER A 149 26.891 50.323 24.211 1.00 0.00 H

ATOM 150 HB3 SER A 149 24.570 49.576 23.684 1.00 0.00 H

ATOM 151 HB2 SER A 149 24.636 51.274 24.113 1.00 0.00 H

ATOM 152 HG SER A 149 23.910 50.738 26.182 1.00 0.00 H

ATOM 153 N ASN A 150 26.213 50.527 27.402 1.00 30.97 N

ATOM 154 CA ASN A 150 26.381 51.318 28.619 1.00 29.82 C

ATOM 155 C ASN A 150 27.388 50.671 29.563 1.00 26.43 C

ATOM 156 O ASN A 150 27.707 49.491 29.427 1.00 26.50 O

ATOM 157 CB ASN A 150 25.049 51.442 29.362 1.00 27.46 C

ATOM 158 CG ASN A 150 24.086 52.403 28.694 1.00 37.24 C

ATOM 159 OD1 ASN A 150 23.188 51.988 27.959 1.00 41.67 O

ATOM 160 ND2 ASN A 150 24.259 53.697 28.957 1.00 36.06 N

ATOM 161 H ASN A 150 25.909 49.569 27.494 1.00 0.00 H

ATOM 162 HA ASN A 150 26.764 52.319 28.408 1.00 0.00 H

ATOM 163 HB3 ASN A 150 25.195 51.792 30.386 1.00 0.00 H

ATOM 164 HB2 ASN A 150 24.572 50.464 29.451 1.00 0.00 H

ATOM 165 HD22 ASN A 150 23.640 54.375 28.536 1.00 0.00 H

ATOM 166 HD21 ASN A 150 25.001 53.999 29.571 1.00 0.00 H

ATOM 167 N SER A 151 27.887 51.450 30.516 1.00 21.71 N

ATOM 168 CA SER A 151 28.649 50.887 31.629 1.00 19.97 C

ATOM 169 C SER A 151 27.953 51.229 32.931 1.00 17.38 C

ATOM 170 O SER A 151 27.236 52.223 33.012 1.00 20.47 O

ATOM 171 CB SER A 151 30.074 51.438 31.657 1.00 20.94 C

ATOM 172 OG SER A 151 30.859 50.947 30.579 1.00 21.71 O

ATOM 173 H SER A 151 27.610 52.419 30.576 1.00 0.00 H

ATOM 174 HA SER A 151 28.702 49.801 31.574 1.00 0.00 H

ATOM 175 HB3 SER A 151 30.561 51.129 32.580 1.00 0.00 H

ATOM 176 HB2 SER A 151 30.081 52.529 31.638 1.00 0.00 H

ATOM 177 HG SER A 151 31.735 51.320 30.641 1.00 0.00 H

ATOM 178 N VAL A 152 28.156 50.390 33.942 1.00 16.90 N

ATOM 179 CA VAL A 152 27.755 50.714 35.307 1.00 20.70 C

ATOM 180 C VAL A 152 29.042 50.915 36.093 1.00 22.40 C

ATOM 181 O VAL A 152 30.008 50.171 35.914 1.00 21.59 O

ATOM 182 CB VAL A 152 26.905 49.586 35.936 1.00 24.12 C

ATOM 183 CG1 VAL A 152 27.480 48.252 35.606 1.00 29.90 C

ATOM 184 CG2 VAL A 152 26.867 49.710 37.413 1.00 28.87 C

ATOM 185 H VAL A 152 28.745 49.578 33.801 1.00 0.00 H

ATOM 186 HA VAL A 152 27.177 51.639 35.364 1.00 0.00 H

ATOM 187 HB VAL A 152 25.895 49.657 35.528 1.00 0.00 H

ATOM 188 HG11 VAL A 152 26.620 47.601 35.636 1.00 0.00 H

ATOM 189 HG12 VAL A 152 27.883 48.097 34.612 1.00 0.00 H

ATOM 190 HG13 VAL A 152 28.131 47.915 36.398 1.00 0.00 H

ATOM 191 HG21 VAL A 152 26.184 48.977 37.843 1.00 0.00 H

ATOM 192 HG22 VAL A 152 27.828 49.547 37.877 1.00 0.00 H

ATOM 193 HG23 VAL A 152 26.508 50.693 37.721 1.00 0.00 H

ATOM 194 N TYR A 153 29.074 51.936 36.939 1.00 14.57 N

ATOM 195 CA TYR A 153 30.244 52.191 37.766 1.00 15.20 C

ATOM 196 C TYR A 153 29.867 51.874 39.198 1.00 18.93 C

ATOM 197 O TYR A 153 28.863 52.384 39.701 1.00 19.83 O

ATOM 198 CB TYR A 153 30.669 53.656 37.639 1.00 15.70 C

ATOM 199 CG TYR A 153 31.133 54.005 36.244 1.00 16.13 C

ATOM 200 CD1 TYR A 153 30.218 54.223 35.221 1.00 17.28 C

ATOM 201 CD2 TYR A 153 32.484 54.112 35.953 1.00 20.90 C

ATOM 202 CE1 TYR A 153 30.638 54.528 33.936 1.00 21.80 C

ATOM 203 CE2 TYR A 153 32.916 54.419 34.679 1.00 24.94 C

ATOM 204 CZ TYR A 153 31.991 54.623 33.676 1.00 23.49 C

ATOM 205 OH TYR A 153 32.424 54.930 32.405 1.00 27.87 O

ATOM 206 H TYR A 153 28.261 52.521 37.063 1.00 0.00 H

ATOM 207 HA TYR A 153 31.102 51.588 37.480 1.00 0.00 H

ATOM 208 HB3 TYR A 153 31.474 53.873 38.343 1.00 0.00 H

ATOM 209 HB2 TYR A 153 29.847 54.322 37.908 1.00 0.00 H

ATOM 210 HD1 TYR A 153 29.158 54.157 35.419 1.00 0.00 H

ATOM 211 HD2 TYR A 153 33.217 53.945 36.729 1.00 0.00 H

ATOM 212 HE1 TYR A 153 29.914 54.691 33.151 1.00 0.00 H

ATOM 213 HE2 TYR A 153 33.974 54.495 34.472 1.00 0.00 H

ATOM 214 HH TYR A 153 33.362 54.860 32.302 1.00 0.00 H

ATOM 215 N VAL A 154 30.639 51.002 39.845 1.00 15.16 N

ATOM 216 CA VAL A 154 30.371 50.658 41.240 1.00 16.49 C

ATOM 217 C VAL A 154 31.659 50.762 42.051 1.00 16.42 C

ATOM 218 O VAL A 154 32.743 50.804 41.483 1.00 17.17 O

ATOM 219 CB VAL A 154 29.773 49.240 41.384 1.00 20.65 C

ATOM 220 CG1 VAL A 154 28.485 49.120 40.580 1.00 21.66 C

ATOM 221 CG2 VAL A 154 30.779 48.180 40.952 1.00 19.29 C

ATOM 222 H VAL A 154 31.454 50.606 39.394 1.00 0.00 H

ATOM 223 HA VAL A 154 29.680 51.363 41.707 1.00 0.00 H

ATOM 224 HB VAL A 154 29.522 49.074 42.432 1.00 0.00 H

ATOM 225 HG11 VAL A 154 27.899 48.266 40.920 1.00 0.00 H

ATOM 226 HG12 VAL A 154 27.854 50.006 40.651 1.00 0.00 H

ATOM 227 HG13 VAL A 154 28.744 48.929 39.546 1.00 0.00 H

ATOM 228 HG21 VAL A 154 30.322 47.190 40.991 1.00 0.00 H

ATOM 229 HG22 VAL A 154 31.111 48.334 39.926 1.00 0.00 H

ATOM 230 HG23 VAL A 154 31.654 48.130 41.600 1.00 0.00 H

ATOM 231 N GLN A 155 31.541 50.829 43.373 1.00 16.29 N

ATOM 232 CA GLN A 155 32.723 50.785 44.222 1.00 16.97 C

ATOM 233 C GLN A 155 33.161 49.338 44.452 1.00 17.33 C

ATOM 234 O GLN A 155 32.325 48.428 44.438 1.00 17.81 O

ATOM 235 CB GLN A 155 32.436 51.451 45.566 1.00 23.06 C

ATOM 236 CG GLN A 155 32.026 52.908 45.448 1.00 23.24 C

ATOM 237 CD GLN A 155 33.084 53.737 44.775 1.00 28.26 C

ATOM 238 OE1 GLN A 155 32.947 54.119 43.606 1.00 30.59 O

ATOM 239 NE2 GLN A 155 34.156 54.026 45.503 1.00 29.87 N

ATOM 240 H GLN A 155 30.628 50.779 43.811 1.00 0.00 H

ATOM 241 HA GLN A 155 33.525 51.321 43.731 1.00 0.00 H

ATOM 242 HB3 GLN A 155 33.314 51.376 46.210 1.00 0.00 H

ATOM 243 HB2 GLN A 155 31.645 50.908 46.086 1.00 0.00 H

ATOM 244 HG3 GLN A 155 31.830 53.317 46.440 1.00 0.00 H

ATOM 245 HG2 GLN A 155 31.089 53.001 44.896 1.00 0.00 H

ATOM 246 HE22 GLN A 155 34.898 54.578 45.098 1.00 0.00 H

ATOM 247 HE21 GLN A 155 34.229 53.690 46.452 1.00 0.00 H

ATOM 248 N TYR A 156 34.462 49.142 44.676 1.00 16.08 N

ATOM 249 CA TYR A 156 35.015 47.812 44.939 1.00 14.80 C

ATOM 250 C TYR A 156 34.176 47.035 45.944 1.00 15.49 C

ATOM 251 O TYR A 156 33.822 45.890 45.712 1.00 16.38 O

ATOM 252 CB TYR A 156 36.448 47.898 45.479 1.00 17.78 C

ATOM 253 CG TYR A 156 37.512 48.286 44.476 1.00 20.42 C

ATOM 254 CD1 TYR A 156 37.200 48.484 43.141 1.00 23.00 C

ATOM 255 CD2 TYR A 156 38.837 48.439 44.869 1.00 19.79 C

ATOM 256 CE1 TYR A 156 38.173 48.837 42.225 1.00 27.33 C

ATOM 257 CE2 TYR A 156 39.822 48.788 43.960 1.00 21.46 C

ATOM 258 CZ TYR A 156 39.480 48.984 42.635 1.00 30.64 C

ATOM 259 OH TYR A 156 40.448 49.332 41.718 1.00 32.29 O

ATOM 260 H TYR A 156 35.098 49.930 44.625 1.00 0.00 H

ATOM 261 HA TYR A 156 34.984 47.224 44.022 1.00 0.00 H

ATOM 262 HB3 TYR A 156 36.746 46.927 45.872 1.00 0.00 H

ATOM 263 HB2 TYR A 156 36.496 48.592 46.320 1.00 0.00 H

ATOM 264 HD1 TYR A 156 36.197 48.395 42.757 1.00 0.00 H

ATOM 265 HD2 TYR A 156 39.108 48.289 45.904 1.00 0.00 H

ATOM 266 HE1 TYR A 156 37.916 49.007 41.191 1.00 0.00 H

ATOM 267 HE2 TYR A 156 40.843 48.909 44.291 1.00 0.00 H

ATOM 268 HH TYR A 156 41.314 49.379 42.094 1.00 0.00 H

ATOM 269 N ASP A 157 33.859 47.654 47.077 1.00 15.43 N

ATOM 270 CA ASP A 157 33.207 46.897 48.132 1.00 13.78 C

ATOM 271 C ASP A 157 31.693 46.773 47.971 1.00 13.74 C

ATOM 272 O ASP A 157 31.007 46.215 48.834 1.00 14.35 O

ATOM 273 CB ASP A 157 33.624 47.404 49.520 1.00 16.05 C

ATOM 274 CG ASP A 157 35.079 47.091 49.833 1.00 17.66 C

ATOM 275 OD1 ASP A 157 35.739 46.369 49.039 1.00 14.37 O

ATOM 276 OD2 ASP A 157 35.564 47.561 50.882 1.00 16.21 O1-

ATOM 277 H ASP A 157 34.155 48.603 47.252 1.00 0.00 H

ATOM 278 HA ASP A 157 33.506 45.852 48.104 1.00 0.00 H

ATOM 279 HB3 ASP A 157 33.024 46.939 50.304 1.00 0.00 H

ATOM 280 HB2 ASP A 157 33.463 48.480 49.598 1.00 0.00 H

ATOM 281 N ASP A 158 31.178 47.253 46.835 1.00 16.15 N

ATOM 282 CA ASP A 158 29.813 46.931 46.426 1.00 16.57 C

ATOM 283 C ASP A 158 29.783 45.520 45.849 1.00 14.99 C

ATOM 284 O ASP A 158 28.713 44.925 45.695 1.00 15.24 O

ATOM 285 CB ASP A 158 29.309 47.882 45.337 1.00 16.72 C

ATOM 286 CG ASP A 158 29.084 49.292 45.832 1.00 22.73 C

ATOM 287 OD1 ASP A 158 28.939 49.493 47.052 1.00 25.06 O

ATOM 288 OD2 ASP A 158 29.040 50.200 44.976 1.00 22.81 O1-

ATOM 289 H ASP A 158 31.778 47.721 46.168 1.00 0.00 H

ATOM 290 HA ASP A 158 29.132 46.952 47.280 1.00 0.00 H

ATOM 291 HB3 ASP A 158 28.340 47.540 44.970 1.00 0.00 H

ATOM 292 HB2 ASP A 158 29.960 47.884 44.466 1.00 0.00 H

ATOM 293 N ILE A 159 30.964 44.999 45.520 1.00 13.02 N

ATOM 294 CA ILE A 159 31.066 43.709 44.855 1.00 13.34 C

ATOM 295 C ILE A 159 31.380 42.615 45.852 1.00 13.20 C

ATOM 296 O ILE A 159 32.387 42.689 46.561 1.00 12.18 O

ATOM 297 CB ILE A 159 32.170 43.724 43.800 1.00 12.49 C

ATOM 298 CG1 ILE A 159 31.869 44.802 42.750 1.00 16.88 C

ATOM 299 CG2 ILE A 159 32.315 42.342 43.160 1.00 15.56 C

ATOM 300 CD1 ILE A 159 33.068 45.209 41.916 1.00 21.97 C

ATOM 301 H ILE A 159 31.811 45.530 45.676 1.00 0.00 H

ATOM 302 HA ILE A 159 30.134 43.464 44.339 1.00 0.00 H

ATOM 303 HB ILE A 159 33.121 43.969 44.272 1.00 0.00 H

ATOM 304 HG13 ILE A 159 31.463 45.716 43.184 1.00 0.00 H

ATOM 305 HG12 ILE A 159 31.085 44.432 42.088 1.00 0.00 H

ATOM 306 HG21 ILE A 159 32.939 42.378 42.269 1.00 0.00 H

ATOM 307 HG22 ILE A 159 32.795 41.620 43.821 1.00 0.00 H

ATOM 308 HG23 ILE A 159 31.351 41.935 42.852 1.00 0.00 H

ATOM 309 HD11 ILE A 159 32.787 45.400 40.880 1.00 0.00 H

ATOM 310 HD12 ILE A 159 33.514 46.115 42.327 1.00 0.00 H

ATOM 311 HD13 ILE A 159 33.841 44.450 41.944 1.00 0.00 H

ATOM 312 N MET A 160 30.519 41.607 45.904 1.00 10.74 N

ATOM 313 CA MET A 160 30.792 40.415 46.701 1.00 11.54 C

ATOM 314 C MET A 160 31.849 39.573 46.002 1.00 12.93 C

ATOM 315 O MET A 160 32.895 39.259 46.576 1.00 11.92 O

ATOM 316 CB MET A 160 29.525 39.576 46.890 1.00 14.42 C

ATOM 317 CG MET A 160 28.338 40.321 47.520 1.00 12.79 C

ATOM 318 SD MET A 160 28.574 40.869 49.226 1.00 13.79 S

ATOM 319 CE MET A 160 28.618 42.631 48.966 1.00 18.65 C

ATOM 320 H MET A 160 29.707 41.604 45.297 1.00 0.00 H

ATOM 321 HA MET A 160 31.165 40.704 47.685 1.00 0.00 H

ATOM 322 HB3 MET A 160 29.752 38.706 47.506 1.00 0.00 H

ATOM 323 HB2 MET A 160 29.183 39.178 45.933 1.00 0.00 H

ATOM 324 HG3 MET A 160 27.506 39.624 47.517 1.00 0.00 H

ATOM 325 HG2 MET A 160 28.020 41.158 46.897 1.00 0.00 H

ATOM 326 HE1 MET A 160 28.790 43.147 49.910 1.00 0.00 H

ATOM 327 HE2 MET A 160 29.413 42.901 48.272 1.00 0.00 H

ATOM 328 HE3 MET A 160 27.671 42.979 48.556 1.00 0.00 H

ATOM 329 N PHE A 161 31.546 39.185 44.767 1.00 11.79 N

ATOM 330 CA PHE A 161 32.483 38.423 43.963 1.00 11.87 C

ATOM 331 C PHE A 161 32.131 38.539 42.496 1.00 10.39 C

ATOM 332 O PHE A 161 31.053 39.011 42.149 1.00 12.15 O

ATOM 333 CB PHE A 161 32.551 36.952 44.407 1.00 12.71 C

ATOM 334 CG PHE A 161 31.249 36.195 44.284 1.00 11.38 C

ATOM 335 CD1 PHE A 161 31.001 35.403 43.168 1.00 12.44 C

ATOM 336 CD2 PHE A 161 30.297 36.238 45.293 1.00 13.39 C

ATOM 337 CE1 PHE A 161 29.820 34.680 43.062 1.00 13.16 C

ATOM 338 CE2 PHE A 161 29.106 35.516 45.184 1.00 12.14 C

ATOM 339 CZ PHE A 161 28.874 34.737 44.065 1.00 11.01 C

ATOM 340 H PHE A 161 30.672 39.467 44.345 1.00 0.00 H

ATOM 341 HA PHE A 161 33.473 38.871 44.078 1.00 0.00 H

ATOM 342 HB3 PHE A 161 32.879 36.885 45.443 1.00 0.00 H

ATOM 343 HB2 PHE A 161 33.307 36.412 43.838 1.00 0.00 H

ATOM 344 HD1 PHE A 161 31.731 35.342 42.375 1.00 0.00 H

ATOM 345 HD2 PHE A 161 30.474 36.833 46.176 1.00 0.00 H

ATOM 346 HE1 PHE A 161 29.640 34.067 42.191 1.00 0.00 H

ATOM 347 HE2 PHE A 161 28.375 35.563 45.977 1.00 0.00 H

ATOM 348 HZ PHE A 161 27.957 34.172 43.980 1.00 0.00 H

ATOM 349 N PHE A 162 33.085 38.154 41.654 1.00 10.26 N

ATOM 350 CA PHE A 162 32.879 38.041 40.211 1.00 10.06 C

ATOM 351 C PHE A 162 32.817 36.571 39.832 1.00 10.84 C

ATOM 352 O PHE A 162 33.541 35.753 40.399 1.00 11.78 O

ATOM 353 CB PHE A 162 34.060 38.644 39.461 1.00 11.22 C

ATOM 354 CG PHE A 162 34.304 40.104 39.732 1.00 13.56 C

ATOM 355 CD1 PHE A 162 33.512 41.079 39.150 1.00 11.10 C

ATOM 356 CD2 PHE A 162 35.373 40.499 40.532 1.00 12.41 C

ATOM 357 CE1 PHE A 162 33.770 42.445 39.383 1.00 12.68 C

ATOM 358 CE2 PHE A 162 35.633 41.843 40.762 1.00 13.94 C

ATOM 359 CZ PHE A 162 34.841 42.817 40.174 1.00 11.98 C

ATOM 360 H PHE A 162 33.965 37.822 42.032 1.00 0.00 H

ATOM 361 HA PHE A 162 31.972 38.555 39.901 1.00 0.00 H

ATOM 362 HB3 PHE A 162 33.907 38.536 38.385 1.00 0.00 H

ATOM 363 HB2 PHE A 162 34.971 38.088 39.679 1.00 0.00 H

ATOM 364 HD1 PHE A 162 32.678 40.790 38.528 1.00 0.00 H

ATOM 365 HD2 PHE A 162 36.010 39.757 40.989 1.00 0.00 H

ATOM 366 HE1 PHE A 162 33.148 43.199 38.923 1.00 0.00 H

ATOM 367 HE2 PHE A 162 36.472 42.131 41.377 1.00 0.00 H

ATOM 368 HZ PHE A 162 35.081 43.859 40.315 1.00 0.00 H

ATOM 369 N GLU A 163 31.986 36.249 38.836 1.00 10.31 N

ATOM 370 CA GLU A 163 31.783 34.872 38.407 1.00 10.63 C

ATOM 371 C GLU A 163 31.890 34.730 36.899 1.00 11.95 C

ATOM 372 O GLU A 163 31.366 35.562 36.153 1.00 13.19 O

ATOM 373 CB GLU A 163 30.373 34.420 38.813 1.00 12.34 C

ATOM 374 CG GLU A 163 29.974 33.062 38.266 1.00 14.26 C

ATOM 375 CD GLU A 163 28.559 32.677 38.640 1.00 20.76 C

ATOM 376 OE1 GLU A 163 27.809 33.556 39.118 1.00 19.28 O

ATOM 377 OE2 GLU A 163 28.189 31.498 38.449 1.00 20.89 O1-

ATOM 378 H GLU A 163 31.415 36.969 38.411 1.00 0.00 H

ATOM 379 HA GLU A 163 32.479 34.210 38.899 1.00 0.00 H

ATOM 380 HB3 GLU A 163 29.659 35.175 38.495 1.00 0.00 H

ATOM 381 HB2 GLU A 163 30.317 34.401 39.902 1.00 0.00 H

ATOM 382 HG3 GLU A 163 30.670 32.288 38.588 1.00 0.00 H

ATOM 383 HG2 GLU A 163 29.959 33.045 37.178 1.00 0.00 H

ATOM 384 N SER A 164 32.582 33.685 36.460 1.00 12.81 N

ATOM 385 CA ASER A 164 32.527 33.249 35.069 0.55 15.71 C

ATOM 386 CA BSER A 164 32.499 33.271 35.067 0.45 15.71 C

ATOM 387 C SER A 164 31.563 32.072 34.985 1.00 17.64 C

ATOM 388 O SER A 164 31.597 31.178 35.835 1.00 17.20 O

ATOM 389 CB ASER A 164 33.915 32.828 34.584 0.55 16.35 C

ATOM 390 CB BSER A 164 33.874 32.904 34.525 0.45 16.38 C

ATOM 391 OG ASER A 164 33.862 32.283 33.276 0.55 18.12 O

ATOM 392 OG BSER A 164 34.300 31.678 35.076 0.45 13.63 O

ATOM 393 H SER A 164 33.012 33.065 37.136 1.00 0.00 H

ATOM 394 HA ASER A 164 32.170 34.043 34.410 0.55 0.00 H

ATOM 395 HA BSER A 164 32.087 34.088 34.475 0.45 0.00 H

ATOM 396 HB3ASER A 164 34.352 32.085 35.251 0.55 0.00 H

ATOM 397 HB3BSER A 164 34.587 33.686 34.785 0.45 0.00 H

ATOM 398 HB2ASER A 164 34.588 33.686 34.576 0.55 0.00 H

ATOM 399 HB2BSER A 164 33.825 32.817 33.440 0.45 0.00 H

ATOM 400 HG ASER A 164 34.738 32.050 33.008 0.55 0.00 H

ATOM 401 HG BSER A 164 35.167 31.455 34.729 0.45 0.00 H

ATOM 402 N SER A 165 30.712 32.065 33.966 1.00 19.18 N

ATOM 403 CA SER A 165 29.741 30.989 33.792 1.00 20.57 C

ATOM 404 C SER A 165 29.824 30.456 32.380 1.00 20.47 C

ATOM 405 O SER A 165 30.046 31.219 31.458 1.00 19.76 O

ATOM 406 CB SER A 165 28.331 31.506 34.047 1.00 23.79 C

ATOM 407 OG SER A 165 27.369 30.574 33.581 1.00 25.85 O

ATOM 408 H SER A 165 30.744 32.798 33.271 1.00 0.00 H

ATOM 409 HA SER A 165 29.921 30.147 34.463 1.00 0.00 H

ATOM 410 HB3 SER A 165 28.164 32.454 33.532 1.00 0.00 H

ATOM 411 HB2 SER A 165 28.177 31.687 35.112 1.00 0.00 H

ATOM 412 HG SER A 165 26.511 30.920 33.778 1.00 0.00 H

ATOM 413 N THR A 166 29.638 29.150 32.211 1.00 19.85 N

ATOM 414 CA THR A 166 29.632 28.575 30.868 1.00 21.71 C

ATOM 415 C THR A 166 28.364 28.967 30.122 1.00 23.37 C

ATOM 416 O THR A 166 28.255 28.737 28.917 1.00 28.00 O

ATOM 417 CB THR A 166 29.718 27.048 30.906 1.00 28.14 C

ATOM 418 OG1 THR A 166 28.580 26.538 31.604 1.00 27.59 O

ATOM 419 CG2 THR A 166 30.998 26.599 31.617 1.00 29.17 C

ATOM 420 H THR A 166 29.441 28.549 32.998 1.00 0.00 H

ATOM 421 HA THR A 166 30.487 28.953 30.303 1.00 0.00 H

ATOM 422 HB THR A 166 29.714 26.641 29.893 1.00 0.00 H

ATOM 423 HG1 THR A 166 28.627 25.594 31.608 1.00 0.00 H

ATOM 424 HG21 THR A 166 31.090 25.513 31.598 1.00 0.00 H

ATOM 425 HG22 THR A 166 31.882 27.010 31.129 1.00 0.00 H

ATOM 426 HG23 THR A 166 31.021 26.907 32.663 1.00 0.00 H

ATOM 427 N LYS A 167 27.403 29.558 30.826 1.00 21.24 N

ATOM 428 CA LYS A 167 26.141 29.930 30.182 1.00 21.49 C

ATOM 429 C LYS A 167 26.131 31.361 29.640 1.00 18.68 C

ATOM 430 O LYS A 167 25.129 31.814 29.083 1.00 18.79 O

ATOM 431 CB LYS A 167 24.968 29.694 31.129 1.00 26.51 C

ATOM 432 CG LYS A 167 24.795 28.220 31.492 1.00 32.74 C

ATOM 433 CD LYS A 167 23.865 28.026 32.676 1.00 39.53 C

ATOM 434 CE LYS A 167 24.551 28.385 33.982 1.00 43.89 C

ATOM 435 NZ LYS A 167 23.659 28.158 35.150 1.00 44.18 N1+

ATOM 436 H LYS A 167 27.530 29.742 31.813 1.00 0.00 H

ATOM 437 HA LYS A 167 25.944 29.294 29.317 1.00 0.00 H

ATOM 438 HB3 LYS A 167 24.036 30.046 30.684 1.00 0.00 H

ATOM 439 HB2 LYS A 167 25.117 30.306 32.017 1.00 0.00 H

ATOM 440 HG3 LYS A 167 25.752 27.745 31.712 1.00 0.00 H

ATOM 441 HG2 LYS A 167 24.395 27.692 30.626 1.00 0.00 H

ATOM 442 HD3 LYS A 167 23.558 26.980 32.713 1.00 0.00 H

ATOM 443 HD2 LYS A 167 22.954 28.610 32.542 1.00 0.00 H

ATOM 444 HE3 LYS A 167 24.849 29.432 34.003 1.00 0.00 H

ATOM 445 HE2 LYS A 167 25.456 27.791 34.116 1.00 0.00 H

ATOM 446 HZ1 LYS A 167 23.391 27.185 35.191 1.00 0.00 H

ATOM 447 HZ2 LYS A 167 24.145 28.401 36.002 1.00 0.00 H

ATOM 448 HZ3 LYS A 167 22.831 28.729 35.065 1.00 0.00 H

ATOM 449 N SER A 168 27.248 32.064 29.792 1.00 18.03 N

ATOM 450 CA SER A 168 27.357 33.421 29.270 1.00 16.30 C

ATOM 451 C SER A 168 28.753 33.729 28.798 1.00 14.51 C

ATOM 452 O SER A 168 29.732 33.231 29.357 1.00 18.73 O

ATOM 453 CB SER A 168 26.976 34.463 30.330 1.00 16.61 C

ATOM 454 OG SER A 168 26.866 35.738 29.722 1.00 16.00 O

ATOM 455 H SER A 168 28.057 31.652 30.237 1.00 0.00 H

ATOM 456 HA SER A 168 26.699 33.541 28.405 1.00 0.00 H

ATOM 457 HB3 SER A 168 27.694 34.497 31.151 1.00 0.00 H

ATOM 458 HB2 SER A 168 26.006 34.220 30.766 1.00 0.00 H

ATOM 459 HG SER A 168 26.434 36.332 30.326 1.00 0.00 H

ATOM 460 N HIS A 169 28.839 34.582 27.784 1.00 12.77 N

ATOM 461 CA HIS A 169 30.110 35.132 27.344 1.00 14.96 C

ATOM 462 C HIS A 169 30.525 36.331 28.210 1.00 15.86 C

ATOM 463 O HIS A 169 31.632 36.853 28.063 1.00 20.77 O

ATOM 464 CB HIS A 169 30.014 35.559 25.868 1.00 14.74 C

ATOM 465 CG HIS A 169 28.990 36.621 25.619 1.00 12.81 C

ATOM 466 ND1 HIS A 169 27.668 36.479 25.986 1.00 12.40 N

ATOM 467 CD2 HIS A 169 29.098 37.856 25.077 1.00 14.74 C

ATOM 468 CE1 HIS A 169 27.004 37.576 25.669 1.00 14.19 C

ATOM 469 NE2 HIS A 169 27.848 38.428 25.115 1.00 15.05 N

ATOM 470 H HIS A 169 27.995 34.962 27.376 1.00 0.00 H

ATOM 471 HA HIS A 169 30.895 34.377 27.420 1.00 0.00 H

ATOM 472 HB3 HIS A 169 29.765 34.695 25.251 1.00 0.00 H

ATOM 473 HB2 HIS A 169 30.982 35.915 25.513 1.00 0.00 H

ATOM 474 HD2 HIS A 169 29.954 38.360 24.659 1.00 0.00 H

ATOM 475 HE1 HIS A 169 25.949 37.737 25.829 1.00 0.00 H

ATOM 476 HE2 HIS A 169 27.618 39.352 24.777 1.00 0.00 H

ATOM 477 N ARG A 170 29.634 36.771 29.099 1.00 14.18 N

ATOM 478 CA ARG A 170 29.922 37.901 29.987 1.00 14.55 C

ATOM 479 C ARG A 170 30.215 37.354 31.375 1.00 15.73 C

ATOM 480 O ARG A 170 29.713 36.299 31.755 1.00 15.25 O

ATOM 481 CB ARG A 170 28.738 38.885 30.055 1.00 14.07 C

ATOM 482 CG ARG A 170 28.564 39.782 28.794 1.00 14.00 C

ATOM 483 CD ARG A 170 27.251 40.587 28.875 1.00 15.41 C

ATOM 484 NE ARG A 170 26.124 39.672 28.970 1.00 16.87 N

ATOM 485 CZ ARG A 170 25.226 39.457 28.010 1.00 19.58 C

ATOM 486 NH1 ARG A 170 25.256 40.152 26.871 1.00 20.03 N

ATOM 487 NH2 ARG A 170 24.276 38.555 28.211 1.00 16.82 N1+

ATOM 488 H ARG A 170 28.743 36.300 29.193 1.00 0.00 H

ATOM 489 HA ARG A 170 30.796 38.469 29.661 1.00 0.00 H

ATOM 490 HB3 ARG A 170 28.842 39.538 30.920 1.00 0.00 H

ATOM 491 HB2 ARG A 170 27.844 38.296 30.241 1.00 0.00 H

ATOM 492 HG3 ARG A 170 28.565 39.169 27.892 1.00 0.00 H

ATOM 493 HG2 ARG A 170 29.412 40.462 28.709 1.00 0.00 H

ATOM 494 HD3 ARG A 170 27.173 41.267 28.029 1.00 0.00 H

ATOM 495 HD2 ARG A 170 27.259 41.217 29.765 1.00 0.00 H

ATOM 496 HE ARG A 170 26.049 39.108 29.816 1.00 0.00 H

ATOM 497 HH12 ARG A 170 24.559 40.000 26.157 1.00 0.00 H

ATOM 498 HH11 ARG A 170 25.973 40.848 26.728 1.00 0.00 H

ATOM 499 HH22 ARG A 170 23.569 38.369 27.516 1.00 0.00 H

ATOM 500 HH21 ARG A 170 24.273 38.034 29.081 1.00 0.00 H

ATOM 501 N LEU A 171 31.032 38.076 32.133 1.00 13.15 N

ATOM 502 CA LEU A 171 31.196 37.762 33.547 1.00 12.59 C

ATOM 503 C LEU A 171 30.004 38.304 34.306 1.00 13.75 C

ATOM 504 O LEU A 171 29.258 39.134 33.791 1.00 14.58 O

ATOM 505 CB LEU A 171 32.460 38.422 34.092 1.00 13.55 C

ATOM 506 CG LEU A 171 33.772 37.949 33.483 1.00 15.45 C

ATOM 507 CD1 LEU A 171 34.887 38.692 34.180 1.00 15.45 C

ATOM 508 CD2 LEU A 171 33.911 36.454 33.692 1.00 15.38 C

ATOM 509 H LEU A 171 31.391 38.958 31.790 1.00 0.00 H

ATOM 510 HA LEU A 171 31.233 36.684 33.709 1.00 0.00 H

ATOM 511 HB3 LEU A 171 32.505 38.270 35.173 1.00 0.00 H

ATOM 512 HB2 LEU A 171 32.383 39.502 33.952 1.00 0.00 H

ATOM 513 HG LEU A 171 33.803 38.172 32.416 1.00 0.00 H

ATOM 514 HD11 LEU A 171 35.860 38.320 33.871 1.00 0.00 H

ATOM 515 HD12 LEU A 171 34.848 39.759 33.961 1.00 0.00 H

ATOM 516 HD13 LEU A 171 34.829 38.579 35.262 1.00 0.00 H

ATOM 517 HD21 LEU A 171 34.903 36.114 33.393 1.00 0.00 H

ATOM 518 HD22 LEU A 171 33.775 36.177 34.738 1.00 0.00 H

ATOM 519 HD23 LEU A 171 33.201 35.887 33.090 1.00 0.00 H

ATOM 520 N ILE A 172 29.830 37.835 35.541 1.00 11.78 N

ATOM 521 CA ILE A 172 28.753 38.303 36.408 1.00 12.79 C

ATOM 522 C ILE A 172 29.313 38.931 37.682 1.00 13.79 C

ATOM 523 O ILE A 172 30.014 38.264 38.459 1.00 13.18 O

ATOM 524 CB ILE A 172 27.805 37.154 36.807 1.00 13.18 C

ATOM 525 CG1 ILE A 172 27.258 36.432 35.579 1.00 15.33 C

ATOM 526 CG2 ILE A 172 26.625 37.682 37.652 1.00 14.78 C

ATOM 527 CD1 ILE A 172 26.630 35.097 35.935 1.00 24.06 C

ATOM 528 H ILE A 172 30.458 37.124 35.897 1.00 0.00 H

ATOM 529 HA ILE A 172 28.142 39.058 35.912 1.00 0.00 H

ATOM 530 HB ILE A 172 28.359 36.431 37.399 1.00 0.00 H

ATOM 531 HG13 ILE A 172 28.022 36.241 34.826 1.00 0.00 H

ATOM 532 HG12 ILE A 172 26.489 37.030 35.115 1.00 0.00 H

ATOM 533 HG21 ILE A 172 25.945 36.884 37.937 1.00 0.00 H

ATOM 534 HG22 ILE A 172 26.940 38.142 38.588 1.00 0.00 H

ATOM 535 HG23 ILE A 172 26.054 38.424 37.093 1.00 0.00 H

ATOM 536 HD11 ILE A 172 26.310 34.578 35.031 1.00 0.00 H

ATOM 537 HD12 ILE A 172 27.343 34.448 36.444 1.00 0.00 H

ATOM 538 HD13 ILE A 172 25.751 35.197 36.570 1.00 0.00 H

ATOM 539 N ALA A 173 29.023 40.214 37.885 1.00 11.55 N

ATOM 540 CA ALA A 173 29.339 40.883 39.142 1.00 11.81 C

ATOM 541 C ALA A 173 28.195 40.682 40.118 1.00 14.18 C

ATOM 542 O ALA A 173 27.070 41.075 39.844 1.00 13.96 O

ATOM 543 CB ALA A 173 29.604 42.347 38.922 1.00 12.73 C

ATOM 544 H ALA A 173 28.458 40.713 37.209 1.00 0.00 H

ATOM 545 HA ALA A 173 30.252 40.468 39.575 1.00 0.00 H

ATOM 546 HB1 ALA A 173 29.772 42.865 39.868 1.00 0.00 H

ATOM 547 HB2 ALA A 173 30.493 42.508 38.316 1.00 0.00 H

ATOM 548 HB3 ALA A 173 28.769 42.839 38.421 1.00 0.00 H

ATOM 549 N HIS A 174 28.493 40.064 41.260 1.00 12.77 N

ATOM 550 CA HIS A 174 27.498 39.855 42.292 1.00 10.01 C

ATOM 551 C HIS A 174 27.566 41.022 43.250 1.00 15.14 C

ATOM 552 O HIS A 174 28.488 41.125 44.067 1.00 13.79 O

ATOM 553 CB HIS A 174 27.741 38.528 43.023 1.00 13.60 C

ATOM 554 CG HIS A 174 27.491 37.322 42.170 1.00 11.28 C

ATOM 555 ND1 HIS A 174 26.449 36.448 42.401 1.00 14.69 N1+

ATOM 556 CD2 HIS A 174 28.131 36.861 41.069 1.00 13.57 C

ATOM 557 CE1 HIS A 174 26.472 35.490 41.494 1.00 13.62 C

ATOM 558 NE2 HIS A 174 27.484 35.715 40.674 1.00 13.44 N

ATOM 559 H HIS A 174 29.444 39.766 41.442 1.00 0.00 H

ATOM 560 HA HIS A 174 26.490 39.803 41.874 1.00 0.00 H

ATOM 561 HB3 HIS A 174 27.112 38.473 43.902 1.00 0.00 H

ATOM 562 HB2 HIS A 174 28.765 38.471 43.393 1.00 0.00 H

ATOM 563 HD1 HIS A 174 25.794 36.483 43.188 1.00 0.00 H

ATOM 564 HD2 HIS A 174 28.997 37.224 40.539 1.00 0.00 H

ATOM 565 HE1 HIS A 174 25.796 34.649 41.446 1.00 0.00 H

ATOM 566 HE2 HIS A 174 27.742 35.085 39.913 1.00 0.00 H

ATOM 567 N LEU A 175 26.594 41.919 43.133 1.00 14.75 N

ATOM 568 CA LEU A 175 26.535 43.072 44.011 1.00 14.38 C

ATOM 569 C LEU A 175 25.663 42.719 45.204 1.00 17.14 C

ATOM 570 O LEU A 175 25.078 41.643 45.252 1.00 17.23 O

ATOM 571 CB LEU A 175 25.957 44.280 43.271 1.00 14.97 C

ATOM 572 CG LEU A 175 26.583 44.619 41.931 1.00 18.87 C

ATOM 573 CD1 LEU A 175 25.986 45.933 41.424 1.00 21.59 C

ATOM 574 CD2 LEU A 175 28.087 44.692 42.035 1.00 18.47 C

ATOM 575 H LEU A 175 25.842 41.783 42.469 1.00 0.00 H

ATOM 576 HA LEU A 175 27.526 43.327 44.374 1.00 0.00 H

ATOM 577 HB3 LEU A 175 26.057 45.150 43.923 1.00 0.00 H

ATOM 578 HB2 LEU A 175 24.882 44.157 43.131 1.00 0.00 H

ATOM 579 HG LEU A 175 26.333 43.839 41.210 1.00 0.00 H

ATOM 580 HD11 LEU A 175 26.442 46.223 40.480 1.00 0.00 H

ATOM 581 HD12 LEU A 175 24.911 45.845 41.263 1.00 0.00 H

ATOM 582 HD13 LEU A 175 26.154 46.748 42.129 1.00 0.00 H

ATOM 583 HD21 LEU A 175 28.524 45.024 41.093 1.00 0.00 H

ATOM 584 HD22 LEU A 175 28.402 45.397 42.806 1.00 0.00 H

ATOM 585 HD23 LEU A 175 28.537 43.726 42.260 1.00 0.00 H

ATOM 586 N ASP A 176 25.579 43.628 46.167 1.00 20.45 N

ATOM 587 CA ASP A 176 24.837 43.344 47.384 1.00 21.33 C

ATOM 588 C ASP A 176 23.415 42.903 47.073 1.00 22.63 C

ATOM 589 O ASP A 176 22.937 41.899 47.596 1.00 20.71 O

ATOM 590 CB ASP A 176 24.821 44.580 48.286 1.00 25.63 C

ATOM 591 CG ASP A 176 24.268 44.287 49.649 1.00 36.73 C

ATOM 592 OD1 ASP A 176 23.180 44.805 49.975 1.00 47.25 O

ATOM 593 OD2 ASP A 176 24.912 43.517 50.386 1.00 30.83 O1-

ATOM 594 H ASP A 176 26.090 44.497 46.094 1.00 0.00 H

ATOM 595 HA ASP A 176 25.351 42.528 47.897 1.00 0.00 H

ATOM 596 HB3 ASP A 176 24.261 45.397 47.827 1.00 0.00 H

ATOM 597 HB2 ASP A 176 25.838 44.950 48.420 1.00 0.00 H

ATOM 598 N ASN A 177 22.758 43.636 46.175 1.00 24.84 N

ATOM 599 CA ASN A 177 21.336 43.458 45.930 1.00 26.93 C

ATOM 600 C ASN A 177 20.956 42.868 44.573 1.00 30.62 C

ATOM 601 O ASN A 177 19.789 42.559 44.336 1.00 26.96 O

ATOM 602 CB ASN A 177 20.604 44.796 46.132 1.00 30.83 C

ATOM 603 CG ASN A 177 21.283 45.959 45.409 1.00 36.55 C

ATOM 604 OD1 ASN A 177 22.303 45.789 44.739 1.00 26.86 O

ATOM 605 ND2 ASN A 177 20.711 47.155 45.550 1.00 40.53 N

ATOM 606 H ASN A 177 23.212 44.434 45.750 1.00 0.00 H

ATOM 607 HA ASN A 177 20.896 42.773 46.657 1.00 0.00 H

ATOM 608 HB3 ASN A 177 20.593 45.032 47.197 1.00 0.00 H

ATOM 609 HB2 ASN A 177 19.558 44.736 45.827 1.00 0.00 H

ATOM 610 HD22 ASN A 177 21.125 47.960 45.103 1.00 0.00 H

ATOM 611 HD21 ASN A 177 19.882 47.256 46.117 1.00 0.00 H

ATOM 612 N ARG A 178 21.926 42.705 43.681 1.00 20.85 N

ATOM 613 CA ARG A 178 21.613 42.172 42.371 1.00 24.52 C

ATOM 614 C ARG A 178 22.867 41.736 41.636 1.00 17.35 C

ATOM 615 O ARG A 178 23.981 42.018 42.071 1.00 18.64 O

ATOM 616 CB ARG A 178 20.888 43.223 41.540 1.00 26.88 C

ATOM 617 CG ARG A 178 21.752 44.405 41.154 1.00 23.38 C

ATOM 618 CD ARG A 178 21.131 45.146 39.965 1.00 27.90 C

ATOM 619 NE ARG A 178 19.736 45.530 40.202 1.00 27.35 N

ATOM 620 CZ ARG A 178 18.863 45.797 39.232 1.00 30.01 C

ATOM 621 NH1 ARG A 178 17.614 46.145 39.524 1.00 29.76 N

ATOM 622 NH2 ARG A 178 19.240 45.715 37.965 1.00 29.32 N1+

ATOM 623 H ARG A 178 22.884 42.934 43.907 1.00 0.00 H

ATOM 624 HA ARG A 178 20.983 41.286 42.479 1.00 0.00 H

ATOM 625 HB3 ARG A 178 19.983 43.572 42.036 1.00 0.00 H

ATOM 626 HB2 ARG A 178 20.527 42.737 40.631 1.00 0.00 H

ATOM 627 HG3 ARG A 178 22.766 44.148 40.852 1.00 0.00 H

ATOM 628 HG2 ARG A 178 21.861 45.078 42.005 1.00 0.00 H

ATOM 629 HD3 ARG A 178 21.196 44.497 39.091 1.00 0.00 H

ATOM 630 HD2 ARG A 178 21.711 46.041 39.736 1.00 0.00 H

ATOM 631 HE ARG A 178 19.455 45.638 41.166 1.00 0.00 H

ATOM 632 HH12 ARG A 178 16.960 46.377 38.789 1.00 0.00 H

ATOM 633 HH11 ARG A 178 17.305 46.170 40.486 1.00 0.00 H

ATOM 634 HH22 ARG A 178 18.588 45.911 37.219 1.00 0.00 H

ATOM 635 HH21 ARG A 178 20.186 45.448 37.737 1.00 0.00 H

ATOM 636 N GLN A 179 22.674 41.066 40.508 1.00 16.85 N

ATOM 637 CA GLN A 179 23.778 40.660 39.647 1.00 17.66 C

ATOM 638 C GLN A 179 23.776 41.479 38.366 1.00 18.52 C

ATOM 639 O GLN A 179 22.717 41.790 37.817 1.00 21.76 O

ATOM 640 CB GLN A 179 23.671 39.178 39.310 1.00 17.75 C

ATOM 641 CG GLN A 179 23.757 38.280 40.547 1.00 18.26 C

ATOM 642 CD GLN A 179 23.438 36.836 40.247 1.00 23.34 C

ATOM 643 OE1 GLN A 179 23.732 36.332 39.163 1.00 26.13 O

ATOM 644 NE2 GLN A 179 22.834 36.153 41.215 1.00 24.20 N

ATOM 645 H GLN A 179 21.734 40.873 40.195 1.00 0.00 H

ATOM 646 HA GLN A 179 24.728 40.800 40.152 1.00 0.00 H

ATOM 647 HB3 GLN A 179 24.458 38.906 38.612 1.00 0.00 H

ATOM 648 HB2 GLN A 179 22.729 38.993 38.789 1.00 0.00 H

ATOM 649 HG3 GLN A 179 23.074 38.627 41.323 1.00 0.00 H

ATOM 650 HG2 GLN A 179 24.759 38.334 40.972 1.00 0.00 H

ATOM 651 HE22 GLN A 179 22.598 35.183 41.067 1.00 0.00 H

ATOM 652 HE21 GLN A 179 22.634 36.595 42.101 1.00 0.00 H

ATOM 653 N ILE A 180 24.966 41.834 37.901 1.00 14.00 N

ATOM 654 CA ILE A 180 25.115 42.598 36.665 1.00 14.54 C

ATOM 655 C ILE A 180 26.135 41.923 35.753 1.00 15.76 C

ATOM 656 O ILE A 180 27.281 41.718 36.155 1.00 14.25 O

ATOM 657 CB ILE A 180 25.585 44.028 36.972 1.00 15.71 C

ATOM 658 CG1 ILE A 180 24.524 44.754 37.804 1.00 21.32 C

ATOM 659 CG2 ILE A 180 25.879 44.785 35.674 1.00 17.77 C

ATOM 660 CD1 ILE A 180 24.794 46.226 38.024 1.00 25.67 C

ATOM 661 H ILE A 180 25.802 41.561 38.403 1.00 0.00 H

ATOM 662 HA ILE A 180 24.179 42.658 36.106 1.00 0.00 H

ATOM 663 HB ILE A 180 26.509 43.983 37.553 1.00 0.00 H

ATOM 664 HG13 ILE A 180 24.453 44.286 38.786 1.00 0.00 H

ATOM 665 HG12 ILE A 180 23.541 44.644 37.345 1.00 0.00 H

ATOM 666 HG21 ILE A 180 26.454 45.654 35.944 1.00 0.00 H

ATOM 667 HG22 ILE A 180 26.510 44.287 34.943 1.00 0.00 H

ATOM 668 HG23 ILE A 180 24.954 45.098 35.195 1.00 0.00 H

ATOM 669 HD11 ILE A 180 24.135 46.632 38.792 1.00 0.00 H

ATOM 670 HD12 ILE A 180 25.823 46.399 38.336 1.00 0.00 H

ATOM 671 HD13 ILE A 180 24.625 46.804 37.115 1.00 0.00 H

ATOM 672 N GLU A 181 25.731 41.566 34.533 1.00 14.38 N

ATOM 673 CA GLU A 181 26.675 40.974 33.589 1.00 14.46 C

ATOM 674 C GLU A 181 27.491 42.056 32.915 1.00 14.20 C

ATOM 675 O GLU A 181 26.981 43.138 32.647 1.00 18.41 O

ATOM 676 CB GLU A 181 25.953 40.127 32.529 1.00 16.27 C

ATOM 677 CG GLU A 181 25.340 38.870 33.118 1.00 18.40 C

ATOM 678 CD GLU A 181 24.957 37.832 32.084 1.00 20.70 C

ATOM 679 OE1 GLU A 181 25.462 37.863 30.931 1.00 19.61 O

ATOM 680 OE2 GLU A 181 24.147 36.958 32.441 1.00 23.02 O1-

ATOM 681 H GLU A 181 24.776 41.714 34.244 1.00 0.00 H

ATOM 682 HA GLU A 181 27.327 40.320 34.150 1.00 0.00 H

ATOM 683 HB3 GLU A 181 26.685 39.852 31.772 1.00 0.00 H

ATOM 684 HB2 GLU A 181 25.189 40.716 32.018 1.00 0.00 H

ATOM 685 HG3 GLU A 181 24.487 39.105 33.755 1.00 0.00 H

ATOM 686 HG2 GLU A 181 26.100 38.421 33.739 1.00 0.00 H

ATOM 687 N PHE A 182 28.760 41.767 32.645 1.00 12.96 N

ATOM 688 CA PHE A 182 29.646 42.742 32.014 1.00 13.43 C

ATOM 689 C PHE A 182 30.808 42.040 31.315 1.00 13.37 C

ATOM 690 O PHE A 182 31.028 40.837 31.513 1.00 14.97 O

ATOM 691 CB PHE A 182 30.181 43.724 33.082 1.00 12.53 C

ATOM 692 CG PHE A 182 31.150 43.091 34.038 1.00 12.06 C

ATOM 693 CD1 PHE A 182 30.703 42.266 35.059 1.00 11.86 C

ATOM 694 CD2 PHE A 182 32.510 43.291 33.898 1.00 13.11 C

ATOM 695 CE1 PHE A 182 31.597 41.662 35.934 1.00 13.19 C

ATOM 696 CE2 PHE A 182 33.413 42.688 34.764 1.00 13.54 C

ATOM 697 CZ PHE A 182 32.948 41.873 35.788 1.00 14.17 C

ATOM 698 H PHE A 182 29.138 40.859 32.890 1.00 0.00 H

ATOM 699 HA PHE A 182 29.089 43.284 31.249 1.00 0.00 H

ATOM 700 HB3 PHE A 182 29.352 44.152 33.646 1.00 0.00 H

ATOM 701 HB2 PHE A 182 30.678 44.572 32.618 1.00 0.00 H

ATOM 702 HD1 PHE A 182 29.647 42.085 35.186 1.00 0.00 H

ATOM 703 HD2 PHE A 182 32.882 43.928 33.110 1.00 0.00 H

ATOM 704 HE1 PHE A 182 31.240 41.008 36.712 1.00 0.00 H

ATOM 705 HE2 PHE A 182 34.473 42.853 34.640 1.00 0.00 H

ATOM 706 HZ PHE A 182 33.648 41.393 36.452 1.00 0.00 H

ATOM 707 N TYR A 183 31.562 42.794 30.520 1.00 14.11 N

ATOM 708 CA TYR A 183 32.706 42.270 29.790 1.00 13.92 C

ATOM 709 C TYR A 183 33.988 42.450 30.577 1.00 16.16 C

ATOM 710 O TYR A 183 34.345 43.562 30.966 1.00 17.65 O

ATOM 711 CB TYR A 183 32.818 42.955 28.418 1.00 14.56 C

ATOM 712 CG TYR A 183 31.641 42.630 27.524 1.00 14.35 C

ATOM 713 CD1 TYR A 183 30.510 43.433 27.513 1.00 15.53 C

ATOM 714 CD2 TYR A 183 31.647 41.484 26.728 1.00 15.81 C

ATOM 715 CE1 TYR A 183 29.424 43.129 26.715 1.00 16.51 C

ATOM 716 CE2 TYR A 183 30.559 41.162 25.936 1.00 15.75 C

ATOM 717 CZ TYR A 183 29.454 41.985 25.940 1.00 17.66 C

ATOM 718 OH TYR A 183 28.370 41.685 25.153 1.00 18.59 O

ATOM 719 H TYR A 183 31.327 43.772 30.404 1.00 0.00 H

ATOM 720 HA TYR A 183 32.569 41.203 29.596 1.00 0.00 H

ATOM 721 HB3 TYR A 183 33.732 42.641 27.910 1.00 0.00 H

ATOM 722 HB2 TYR A 183 32.893 44.038 28.534 1.00 0.00 H

ATOM 723 HD1 TYR A 183 30.483 44.334 28.100 1.00 0.00 H

ATOM 724 HD2 TYR A 183 32.510 40.834 26.728 1.00 0.00 H

ATOM 725 HE1 TYR A 183 28.567 43.779 26.685 1.00 0.00 H

ATOM 726 HE2 TYR A 183 30.589 40.283 25.317 1.00 0.00 H

ATOM 727 HH TYR A 183 27.733 42.383 25.131 1.00 0.00 H

ATOM 728 N GLY A 184 34.682 41.351 30.824 1.00 16.27 N

ATOM 729 CA GLY A 184 35.954 41.430 31.508 1.00 20.16 C

ATOM 730 C GLY A 184 36.625 40.084 31.592 1.00 17.71 C

ATOM 731 O GLY A 184 36.149 39.110 31.021 1.00 18.66 O

ATOM 732 H GLY A 184 34.362 40.448 30.503 1.00 0.00 H

ATOM 733 HA3 GLY A 184 35.804 41.815 32.519 1.00 0.00 H

ATOM 734 HA2 GLY A 184 36.622 42.118 30.987 1.00 0.00 H

ATOM 735 N ASN A 185 37.737 40.018 32.310 1.00 19.49 N

ATOM 736 CA ASN A 185 38.349 38.727 32.561 1.00 20.86 C

ATOM 737 C ASN A 185 38.812 38.619 33.996 1.00 18.37 C

ATOM 738 O ASN A 185 39.238 39.603 34.603 1.00 18.79 O

ATOM 739 CB ASN A 185 39.472 38.422 31.567 1.00 32.51 C

ATOM 740 CG ASN A 185 40.711 39.231 31.819 1.00 24.34 C

ATOM 741 OD1 ASN A 185 41.629 38.787 32.521 1.00 27.72 O

ATOM 742 ND2 ASN A 185 40.763 40.426 31.240 1.00 36.42 N

ATOM 743 H ASN A 185 38.111 40.835 32.772 1.00 0.00 H

ATOM 744 HA ASN A 185 37.619 37.924 32.439 1.00 0.00 H

ATOM 745 HB3 ASN A 185 39.129 38.573 30.542 1.00 0.00 H

ATOM 746 HB2 ASN A 185 39.741 37.367 31.634 1.00 0.00 H

ATOM 747 HD22 ASN A 185 41.534 41.059 31.416 1.00 0.00 H

ATOM 748 HD21 ASN A 185 39.994 40.744 30.670 1.00 0.00 H

ATOM 749 N LEU A 186 38.679 37.417 34.540 1.00 16.59 N

ATOM 750 CA LEU A 186 38.900 37.200 35.962 1.00 15.47 C

ATOM 751 C LEU A 186 40.326 37.526 36.371 1.00 21.46 C

ATOM 752 O LEU A 186 40.548 38.058 37.456 1.00 16.65 O

ATOM 753 CB LEU A 186 38.551 35.768 36.339 1.00 14.95 C

ATOM 754 CG LEU A 186 37.068 35.394 36.251 1.00 16.54 C

ATOM 755 CD1 LEU A 186 36.880 33.960 36.683 1.00 15.89 C

ATOM 756 CD2 LEU A 186 36.199 36.337 37.086 1.00 17.09 C

ATOM 757 H LEU A 186 38.315 36.655 33.987 1.00 0.00 H

ATOM 758 HA LEU A 186 38.274 37.895 36.521 1.00 0.00 H

ATOM 759 HB3 LEU A 186 38.894 35.572 37.354 1.00 0.00 H

ATOM 760 HB2 LEU A 186 39.124 35.084 35.710 1.00 0.00 H

ATOM 761 HG LEU A 186 36.744 35.474 35.213 1.00 0.00 H

ATOM 762 HD11 LEU A 186 35.832 33.667 36.635 1.00 0.00 H

ATOM 763 HD12 LEU A 186 37.441 33.276 36.046 1.00 0.00 H

ATOM 764 HD13 LEU A 186 37.211 33.804 37.711 1.00 0.00 H

ATOM 765 HD21 LEU A 186 35.162 35.999 37.095 1.00 0.00 H

ATOM 766 HD22 LEU A 186 36.530 36.384 38.123 1.00 0.00 H

ATOM 767 HD23 LEU A 186 36.185 37.350 36.689 1.00 0.00 H

ATOM 768 N LYS A 187 41.288 37.218 35.505 1.00 21.45 N

ATOM 769 CA LYS A 187 42.690 37.428 35.866 1.00 25.90 C

ATOM 770 C LYS A 187 42.958 38.909 36.082 1.00 22.24 C

ATOM 771 O LYS A 187 43.543 39.311 37.092 1.00 21.80 O

ATOM 772 CB LYS A 187 43.621 36.845 34.795 1.00 26.24 C

ATOM 773 CG LYS A 187 45.094 36.884 35.173 1.00 32.77 C

ATOM 774 CD LYS A 187 45.816 35.659 34.635 1.00 40.20 C

ATOM 775 CE LYS A 187 45.175 34.382 35.167 1.00 46.21 C

ATOM 776 NZ LYS A 187 45.810 33.153 34.619 1.00 51.44 N1+

ATOM 777 H LYS A 187 41.059 36.827 34.603 1.00 0.00 H

ATOM 778 HA LYS A 187 42.877 36.900 36.804 1.00 0.00 H

ATOM 779 HB3 LYS A 187 43.518 37.429 33.879 1.00 0.00 H

ATOM 780 HB2 LYS A 187 43.213 35.892 34.488 1.00 0.00 H

ATOM 781 HG3 LYS A 187 45.208 36.902 36.258 1.00 0.00 H

ATOM 782 HG2 LYS A 187 45.559 37.800 34.805 1.00 0.00 H

ATOM 783 HD3 LYS A 187 46.868 35.699 34.919 1.00 0.00 H

ATOM 784 HD2 LYS A 187 45.786 35.667 33.544 1.00 0.00 H

ATOM 785 HE3 LYS A 187 44.127 34.227 34.939 1.00 0.00 H

ATOM 786 HE2 LYS A 187 45.260 34.354 36.254 1.00 0.00 H

ATOM 787 HZ1 LYS A 187 45.377 32.335 35.023 1.00 0.00 H

ATOM 788 HZ2 LYS A 187 45.697 33.132 33.615 1.00 0.00 H

ATOM 789 HZ3 LYS A 187 46.795 33.154 34.844 1.00 0.00 H

ATOM 790 N GLU A 188 42.500 39.722 35.141 1.00 22.45 N

ATOM 791 CA GLU A 188 42.651 41.163 35.229 1.00 22.76 C

ATOM 792 C GLU A 188 41.963 41.727 36.471 1.00 22.06 C

ATOM 793 O GLU A 188 42.505 42.595 37.158 1.00 21.05 O

ATOM 794 CB GLU A 188 42.079 41.805 33.969 1.00 27.17 C

ATOM 795 CG GLU A 188 42.046 43.312 33.990 1.00 33.78 C

ATOM 796 CD GLU A 188 41.626 43.889 32.655 1.00 40.90 C

ATOM 797 OE1 GLU A 188 41.388 43.099 31.714 1.00 37.93 O

ATOM 798 OE2 GLU A 188 41.538 45.130 32.548 1.00 55.35 O1-

ATOM 799 H GLU A 188 42.032 39.339 34.329 1.00 0.00 H

ATOM 800 HA GLU A 188 43.715 41.400 35.292 1.00 0.00 H

ATOM 801 HB3 GLU A 188 41.064 41.439 33.809 1.00 0.00 H

ATOM 802 HB2 GLU A 188 42.679 41.455 33.127 1.00 0.00 H

ATOM 803 HG3 GLU A 188 43.031 43.708 34.240 1.00 0.00 H

ATOM 804 HG2 GLU A 188 41.347 43.689 34.737 1.00 0.00 H

ATOM 805 N LEU A 189 40.768 41.229 36.760 1.00 19.90 N

ATOM 806 CA LEU A 189 40.028 41.697 37.934 1.00 19.72 C

ATOM 807 C LEU A 189 40.748 41.370 39.238 1.00 17.29 C

ATOM 808 O LEU A 189 40.772 42.187 40.169 1.00 18.11 O

ATOM 809 CB LEU A 189 38.608 41.125 37.940 1.00 15.85 C

ATOM 810 CG LEU A 189 37.746 41.668 36.808 1.00 16.40 C

ATOM 811 CD1 LEU A 189 36.496 40.822 36.636 1.00 16.74 C

ATOM 812 CD2 LEU A 189 37.395 43.115 37.057 1.00 18.69 C

ATOM 813 H LEU A 189 40.351 40.530 36.160 1.00 0.00 H

ATOM 814 HA LEU A 189 39.975 42.784 37.891 1.00 0.00 H

ATOM 815 HB3 LEU A 189 38.119 41.327 38.895 1.00 0.00 H

ATOM 816 HB2 LEU A 189 38.667 40.038 37.867 1.00 0.00 H

ATOM 817 HG LEU A 189 38.295 41.619 35.869 1.00 0.00 H

ATOM 818 HD11 LEU A 189 35.908 41.168 35.787 1.00 0.00 H

ATOM 819 HD12 LEU A 189 36.747 39.779 36.448 1.00 0.00 H

ATOM 820 HD13 LEU A 189 35.866 40.860 37.522 1.00 0.00 H

ATOM 821 HD21 LEU A 189 36.682 43.471 36.313 1.00 0.00 H

ATOM 822 HD22 LEU A 189 36.939 43.254 38.038 1.00 0.00 H

ATOM 823 HD23 LEU A 189 38.262 43.771 36.991 1.00 0.00 H

ATOM 824 N SER A 190 41.346 40.188 39.318 1.00 19.95 N

ATOM 825 CA SER A 190 42.078 39.811 40.520 1.00 18.40 C

ATOM 826 C SER A 190 43.277 40.738 40.732 1.00 23.96 C

ATOM 827 O SER A 190 43.745 40.913 41.854 1.00 23.77 O

ATOM 828 CB SER A 190 42.530 38.354 40.457 1.00 20.74 C

ATOM 829 OG SER A 190 43.511 38.148 39.450 1.00 24.49 O

ATOM 830 H SER A 190 41.287 39.536 38.546 1.00 0.00 H

ATOM 831 HA SER A 190 41.413 39.914 41.379 1.00 0.00 H

ATOM 832 HB3 SER A 190 41.677 37.705 40.257 1.00 0.00 H

ATOM 833 HB2 SER A 190 42.943 38.040 41.416 1.00 0.00 H

ATOM 834 HG SER A 190 43.217 38.536 38.636 1.00 0.00 H

ATOM 835 N GLN A 191 43.767 41.340 39.655 1.00 20.86 N

ATOM 836 CA GLN A 191 44.911 42.249 39.763 1.00 22.27 C

ATOM 837 C GLN A 191 44.579 43.701 40.103 1.00 27.72 C

ATOM 838 O GLN A 191 45.486 44.497 40.336 1.00 27.97 O

ATOM 839 CB GLN A 191 45.754 42.195 38.486 1.00 25.89 C

ATOM 840 CG GLN A 191 46.382 40.841 38.250 1.00 28.01 C

ATOM 841 CD GLN A 191 47.042 40.730 36.893 1.00 36.18 C

ATOM 842 OE1 GLN A 191 46.919 41.621 36.051 1.00 40.90 O

ATOM 843 NE2 GLN A 191 47.742 39.626 36.671 1.00 41.25 N

ATOM 844 H GLN A 191 43.350 41.185 38.747 1.00 0.00 H

ATOM 845 HA GLN A 191 45.572 41.912 40.564 1.00 0.00 H

ATOM 846 HB3 GLN A 191 46.550 42.941 38.520 1.00 0.00 H

ATOM 847 HB2 GLN A 191 45.118 42.471 37.643 1.00 0.00 H

ATOM 848 HG3 GLN A 191 45.644 40.045 38.329 1.00 0.00 H

ATOM 849 HG2 GLN A 191 47.126 40.646 39.024 1.00 0.00 H

ATOM 850 HE22 GLN A 191 48.205 39.503 35.783 1.00 0.00 H

ATOM 851 HE21 GLN A 191 47.818 38.922 37.391 1.00 0.00 H

ATOM 852 N LEU A 192 43.299 44.058 40.127 1.00 23.09 N

ATOM 853 CA LEU A 192 42.924 45.431 40.437 1.00 20.56 C

ATOM 854 C LEU A 192 43.300 45.834 41.857 1.00 19.98 C

ATOM 855 O LEU A 192 43.612 46.994 42.110 1.00 26.45 O

ATOM 856 CB LEU A 192 41.426 45.661 40.245 1.00 27.25 C

ATOM 857 CG LEU A 192 40.878 45.792 38.829 1.00 29.55 C

ATOM 858 CD1 LEU A 192 39.372 45.956 38.899 1.00 32.48 C

ATOM 859 CD2 LEU A 192 41.511 46.972 38.101 1.00 33.22 C

ATOM 860 H LEU A 192 42.575 43.380 39.929 1.00 0.00 H

ATOM 861 HA LEU A 192 43.478 46.099 39.777 1.00 0.00 H

ATOM 862 HB3 LEU A 192 41.136 46.571 40.775 1.00 0.00 H

ATOM 863 HB2 LEU A 192 40.888 44.863 40.760 1.00 0.00 H

ATOM 864 HG LEU A 192 41.103 44.880 38.274 1.00 0.00 H

ATOM 865 HD11 LEU A 192 38.939 46.039 37.902 1.00 0.00 H

ATOM 866 HD12 LEU A 192 38.905 45.098 39.384 1.00 0.00 H

ATOM 867 HD13 LEU A 192 39.089 46.850 39.457 1.00 0.00 H

ATOM 868 HD21 LEU A 192 41.035 47.131 37.133 1.00 0.00 H

ATOM 869 HD22 LEU A 192 41.412 47.896 38.672 1.00 0.00 H

ATOM 870 HD23 LEU A 192 42.569 46.809 37.901 1.00 0.00 H

ATOM 871 N ASP A 193 43.258 44.888 42.789 1.00 17.26 N

ATOM 872 CA ASP A 193 43.437 45.246 44.194 1.00 18.47 C

ATOM 873 C ASP A 193 43.699 43.999 45.029 1.00 20.69 C

ATOM 874 O ASP A 193 43.189 42.928 44.710 1.00 16.92 O

ATOM 875 CB ASP A 193 42.187 45.959 44.716 1.00 19.57 C

ATOM 876 CG ASP A 193 42.395 46.589 46.089 1.00 23.82 C

ATOM 877 OD1 ASP A 193 42.353 45.858 47.102 1.00 19.70 O

ATOM 878 OD2 ASP A 193 42.596 47.822 46.159 1.00 30.50 O1-

ATOM 879 H ASP A 193 43.028 43.933 42.551 1.00 0.00 H

ATOM 880 HA ASP A 193 44.309 45.899 44.287 1.00 0.00 H

ATOM 881 HB3 ASP A 193 41.347 45.272 44.788 1.00 0.00 H

ATOM 882 HB2 ASP A 193 41.866 46.749 44.039 1.00 0.00 H

ATOM 883 N ASP A 194 44.490 44.143 46.094 1.00 19.13 N

ATOM 884 CA ASP A 194 44.826 43.024 46.975 1.00 20.37 C

ATOM 885 C ASP A 194 43.599 42.341 47.572 1.00 17.86 C

ATOM 886 O ASP A 194 43.657 41.173 47.945 1.00 16.04 O

ATOM 887 CB ASP A 194 45.732 43.500 48.125 1.00 20.19 C

ATOM 888 CG ASP A 194 47.165 43.730 47.686 1.00 27.52 C

ATOM 889 OD1 ASP A 194 47.507 43.358 46.542 1.00 26.19 O

ATOM 890 OD2 ASP A 194 47.955 44.273 48.491 1.00 29.14 O1-

ATOM 891 H ASP A 194 44.871 45.052 46.312 1.00 0.00 H

ATOM 892 HA ASP A 194 45.323 42.252 46.383 1.00 0.00 H

ATOM 893 HB3 ASP A 194 45.770 42.756 48.923 1.00 0.00 H

ATOM 894 HB2 ASP A 194 45.339 44.414 48.573 1.00 0.00 H

ATOM 895 N ARG A 195 42.494 43.071 47.701 1.00 14.82 N

ATOM 896 CA ARG A 195 41.303 42.470 48.295 1.00 14.46 C

ATOM 897 C ARG A 195 40.645 41.447 47.366 1.00 14.94 C

ATOM 898 O ARG A 195 39.892 40.593 47.822 1.00 12.69 O

ATOM 899 CB ARG A 195 40.289 43.546 48.688 1.00 13.48 C

ATOM 900 CG ARG A 195 39.510 44.126 47.510 1.00 14.33 C

ATOM 901 CD ARG A 195 38.757 45.385 47.938 1.00 17.55 C

ATOM 902 NE ARG A 195 39.685 46.492 48.131 1.00 16.47 N

ATOM 903 CZ ARG A 195 39.328 47.699 48.557 1.00 18.57 C

ATOM 904 NH1 ARG A 195 38.061 47.951 48.867 1.00 15.92 N

ATOM 905 NH2 ARG A 195 40.252 48.647 48.681 1.00 21.02 N1+

ATOM 906 H ARG A 195 42.481 44.047 47.421 1.00 0.00 H

ATOM 907 HA ARG A 195 41.592 41.956 49.215 1.00 0.00 H

ATOM 908 HB3 ARG A 195 40.813 44.334 49.231 1.00 0.00 H

ATOM 909 HB2 ARG A 195 39.576 43.127 49.400 1.00 0.00 H

ATOM 910 HG3 ARG A 195 38.789 43.406 47.121 1.00 0.00 H

ATOM 911 HG2 ARG A 195 40.177 44.364 46.682 1.00 0.00 H

ATOM 912 HD3 ARG A 195 38.191 45.198 48.850 1.00 0.00 H

ATOM 913 HD2 ARG A 195 38.042 45.659 47.165 1.00 0.00 H

ATOM 914 HE ARG A 195 40.656 46.321 47.880 1.00 0.00 H

ATOM 915 HH12 ARG A 195 37.785 48.894 49.117 1.00 0.00 H

ATOM 916 HH11 ARG A 195 37.347 47.227 48.830 1.00 0.00 H

ATOM 917 HH22 ARG A 195 39.995 49.596 48.945 1.00 0.00 H

ATOM 918 HH21 ARG A 195 41.208 48.457 48.411 1.00 0.00 H

ATOM 919 N PHE A 196 40.924 41.541 46.069 1.00 15.95 N

ATOM 920 CA PHE A 196 40.342 40.609 45.115 1.00 14.70 C

ATOM 921 C PHE A 196 41.246 39.389 44.969 1.00 15.95 C

ATOM 922 O PHE A 196 42.474 39.513 44.913 1.00 21.27 O

ATOM 923 CB PHE A 196 40.102 41.295 43.765 1.00 15.27 C

ATOM 924 CG PHE A 196 39.070 42.405 43.814 1.00 13.40 C

ATOM 925 CD1 PHE A 196 37.747 42.124 44.128 1.00 15.03 C

ATOM 926 CD2 PHE A 196 39.418 43.720 43.531 1.00 17.45 C

ATOM 927 CE1 PHE A 196 36.795 43.131 44.167 1.00 15.63 C

ATOM 928 CE2 PHE A 196 38.465 44.734 43.580 1.00 21.25 C

ATOM 929 CZ PHE A 196 37.155 44.432 43.897 1.00 14.63 C

ATOM 930 H PHE A 196 41.582 42.234 45.736 1.00 0.00 H

ATOM 931 HA PHE A 196 39.368 40.334 45.467 1.00 0.00 H

ATOM 932 HB3 PHE A 196 39.752 40.565 43.036 1.00 0.00 H

ATOM 933 HB2 PHE A 196 41.041 41.684 43.367 1.00 0.00 H

ATOM 934 HD1 PHE A 196 37.445 41.108 44.336 1.00 0.00 H

ATOM 935 HD2 PHE A 196 40.435 43.958 43.257 1.00 0.00 H

ATOM 936 HE1 PHE A 196 35.765 42.901 44.393 1.00 0.00 H

ATOM 937 HE2 PHE A 196 38.749 45.752 43.358 1.00 0.00 H

ATOM 938 HZ PHE A 196 36.411 45.214 43.925 1.00 0.00 H

ATOM 939 N PHE A 197 40.647 38.206 44.937 1.00 11.66 N

ATOM 940 CA PHE A 197 41.441 36.988 44.958 1.00 11.98 C

ATOM 941 C PHE A 197 40.765 35.897 44.132 1.00 13.13 C

ATOM 942 O PHE A 197 39.595 35.572 44.341 1.00 11.41 O

ATOM 943 CB PHE A 197 41.674 36.522 46.404 1.00 13.43 C

ATOM 944 CG PHE A 197 42.467 35.247 46.513 1.00 15.93 C

ATOM 945 CD1 PHE A 197 43.816 35.233 46.195 1.00 15.91 C

ATOM 946 CD2 PHE A 197 41.868 34.077 46.939 1.00 15.63 C

ATOM 947 CE1 PHE A 197 44.550 34.049 46.292 1.00 17.41 C

ATOM 948 CE2 PHE A 197 42.589 32.893 47.043 1.00 15.86 C

ATOM 949 CZ PHE A 197 43.939 32.885 46.719 1.00 16.65 C

ATOM 950 H PHE A 197 39.640 38.142 45.014 1.00 0.00 H

ATOM 951 HA PHE A 197 42.417 37.156 44.497 1.00 0.00 H

ATOM 952 HB3 PHE A 197 40.718 36.402 46.917 1.00 0.00 H

ATOM 953 HB2 PHE A 197 42.204 37.298 46.955 1.00 0.00 H

ATOM 954 HD1 PHE A 197 44.304 36.138 45.864 1.00 0.00 H

ATOM 955 HD2 PHE A 197 40.819 34.087 47.189 1.00 0.00 H

ATOM 956 HE1 PHE A 197 45.599 34.043 46.034 1.00 0.00 H

ATOM 957 HE2 PHE A 197 42.106 31.985 47.370 1.00 0.00 H

ATOM 958 HZ PHE A 197 44.506 31.969 46.779 1.00 0.00 H

ATOM 959 N ARG A 198 41.508 35.341 43.181 1.00 12.84 N

ATOM 960 CA ARG A 198 41.038 34.202 42.399 1.00 14.29 C

ATOM 961 C ARG A 198 41.092 32.937 43.242 1.00 17.09 C

ATOM 962 O ARG A 198 42.172 32.413 43.515 1.00 15.50 O

ATOM 963 CB ARG A 198 41.921 34.038 41.162 1.00 14.96 C

ATOM 964 CG ARG A 198 41.483 32.921 40.211 1.00 18.11 C

ATOM 965 CD ARG A 198 40.289 33.369 39.391 1.00 16.01 C

ATOM 966 NE ARG A 198 39.981 32.452 38.281 1.00 16.44 N

ATOM 967 CZ ARG A 198 40.526 32.530 37.073 1.00 21.31 C

ATOM 968 NH1 ARG A 198 41.410 33.476 36.811 1.00 21.87 N

ATOM 969 NH2 ARG A 198 40.178 31.655 36.128 1.00 20.47 N1+

ATOM 970 H ARG A 198 42.462 35.647 43.053 1.00 0.00 H

ATOM 971 HA ARG A 198 40.014 34.385 42.075 1.00 0.00 H

ATOM 972 HB3 ARG A 198 42.949 33.844 41.473 1.00 0.00 H

ATOM 973 HB2 ARG A 198 41.970 34.983 40.618 1.00 0.00 H

ATOM 974 HG3 ARG A 198 41.268 31.985 40.727 1.00 0.00 H

ATOM 975 HG2 ARG A 198 42.310 32.701 39.535 1.00 0.00 H

ATOM 976 HD3 ARG A 198 40.431 34.376 39.000 1.00 0.00 H

ATOM 977 HD2 ARG A 198 39.407 33.418 40.020 1.00 0.00 H

ATOM 978 HE ARG A 198 39.326 31.708 38.490 1.00 0.00 H

ATOM 979 HH12 ARG A 198 41.816 33.557 35.891 1.00 0.00 H

ATOM 980 HH11 ARG A 198 41.664 34.138 37.530 1.00 0.00 H

ATOM 981 HH22 ARG A 198 40.590 31.691 35.207 1.00 0.00 H

ATOM 982 HH21 ARG A 198 39.448 30.977 36.311 1.00 0.00 H

ATOM 983 N CYS A 199 39.935 32.440 43.666 1.00 13.55 N

ATOM 984 CA CYS A 199 39.907 31.294 44.575 1.00 13.10 C

ATOM 985 C CYS A 199 39.423 29.997 43.917 1.00 12.18 C

ATOM 986 O CYS A 199 39.325 28.955 44.570 1.00 13.71 O

ATOM 987 CB CYS A 199 39.049 31.617 45.809 1.00 14.26 C

ATOM 988 SG CYS A 199 37.275 31.777 45.469 1.00 15.23 S

ATOM 989 H CYS A 199 39.065 32.896 43.426 1.00 0.00 H

ATOM 990 HA CYS A 199 40.896 31.069 44.971 1.00 0.00 H

ATOM 991 HB3 CYS A 199 39.387 32.547 46.263 1.00 0.00 H

ATOM 992 HB2 CYS A 199 39.182 30.837 46.556 1.00 0.00 H

ATOM 993 HG CYS A 199 37.092 30.528 45.032 1.00 0.00 H

ATOM 994 N HIS A 200 39.087 30.096 42.638 1.00 14.01 N

ATOM 995 CA HIS A 200 38.435 29.024 41.905 1.00 15.39 C

ATOM 996 C HIS A 200 38.522 29.413 40.439 1.00 14.08 C

ATOM 997 O HIS A 200 38.632 30.594 40.113 1.00 13.62 O

ATOM 998 CB HIS A 200 36.972 28.955 42.347 1.00 15.00 C

ATOM 999 CG HIS A 200 36.219 27.772 41.827 1.00 15.22 C

ATOM 1000 ND1 HIS A 200 35.675 27.737 40.560 1.00 16.47 N

ATOM 1001 CD2 HIS A 200 35.883 26.601 42.416 1.00 15.64 C

ATOM 1002 CE1 HIS A 200 35.045 26.588 40.386 1.00 18.44 C

ATOM 1003 NE2 HIS A 200 35.156 25.879 41.496 1.00 19.33 N

ATOM 1004 H HIS A 200 39.191 30.985 42.167 1.00 0.00 H

ATOM 1005 HA HIS A 200 38.950 28.077 42.077 1.00 0.00 H

ATOM 1006 HB3 HIS A 200 36.468 29.872 42.048 1.00 0.00 H

ATOM 1007 HB2 HIS A 200 36.906 28.912 43.430 1.00 0.00 H

ATOM 1008 HD2 HIS A 200 36.087 26.223 43.406 1.00 0.00 H

ATOM 1009 HE1 HIS A 200 34.522 26.287 39.490 1.00 0.00 H

ATOM 1010 HE2 HIS A 200 34.761 24.961 41.642 1.00 0.00 H

ATOM 1011 N ASN A 201 38.486 28.427 39.547 1.00 14.87 N

ATOM 1012 CA ASN A 201 38.494 28.744 38.126 1.00 16.95 C

ATOM 1013 C ASN A 201 37.388 29.731 37.746 1.00 15.73 C

ATOM 1014 O ASN A 201 37.561 30.552 36.848 1.00 17.70 O

ATOM 1015 CB ASN A 201 38.351 27.473 37.292 1.00 18.99 C

ATOM 1016 CG ASN A 201 38.412 27.758 35.808 1.00 27.19 C

ATOM 1017 OD1 ASN A 201 39.420 28.257 35.309 1.00 30.17 O

ATOM 1018 ND2 ASN A 201 37.329 27.459 35.095 1.00 31.52 N

ATOM 1019 H ASN A 201 38.389 27.466 39.841 1.00 0.00 H

ATOM 1020 HA ASN A 201 39.457 29.208 37.899 1.00 0.00 H

ATOM 1021 HB3 ASN A 201 37.421 26.957 37.541 1.00 0.00 H

ATOM 1022 HB2 ASN A 201 39.158 26.780 37.533 1.00 0.00 H

ATOM 1023 HD22 ASN A 201 37.324 27.635 34.101 1.00 0.00 H

ATOM 1024 HD21 ASN A 201 36.518 27.060 35.544 1.00 0.00 H

ATOM 1025 N SER A 202 36.266 29.657 38.459 1.00 15.36 N

ATOM 1026 CA SER A 202 35.088 30.450 38.120 1.00 14.06 C

ATOM 1027 C SER A 202 34.894 31.737 38.930 1.00 13.85 C

ATOM 1028 O SER A 202 33.985 32.511 38.621 1.00 12.27 O

ATOM 1029 CB SER A 202 33.828 29.598 38.284 1.00 18.17 C

ATOM 1030 OG SER A 202 33.818 28.525 37.356 1.00 18.84 O

ATOM 1031 H SER A 202 36.178 28.962 39.191 1.00 0.00 H

ATOM 1032 HA SER A 202 35.122 30.759 37.074 1.00 0.00 H

ATOM 1033 HB3 SER A 202 32.921 30.180 38.115 1.00 0.00 H

ATOM 1034 HB2 SER A 202 33.755 29.197 39.294 1.00 0.00 H

ATOM 1035 HG SER A 202 33.709 28.882 36.488 1.00 0.00 H

ATOM 1036 N PHE A 203 35.698 31.955 39.974 1.00 13.06 N

ATOM 1037 CA PHE A 203 35.377 33.042 40.914 1.00 11.28 C

ATOM 1038 C PHE A 203 36.544 33.887 41.342 1.00 12.36 C

ATOM 1039 O PHE A 203 37.614 33.368 41.667 1.00 12.82 O

ATOM 1040 CB PHE A 203 34.699 32.514 42.185 1.00 12.25 C

ATOM 1041 CG PHE A 203 33.503 31.666 41.927 1.00 12.84 C

ATOM 1042 CD1 PHE A 203 32.316 32.216 41.466 1.00 12.26 C

ATOM 1043 CD2 PHE A 203 33.558 30.306 42.165 1.00 13.12 C

ATOM 1044 CE1 PHE A 203 31.210 31.401 41.229 1.00 11.74 C

ATOM 1045 CE2 PHE A 203 32.472 29.496 41.937 1.00 14.28 C

ATOM 1046 CZ PHE A 203 31.292 30.041 41.462 1.00 12.80 C

ATOM 1047 H PHE A 203 36.453 31.319 40.190 1.00 0.00 H

ATOM 1048 HA PHE A 203 34.675 33.725 40.457 1.00 0.00 H

ATOM 1049 HB3 PHE A 203 34.383 33.347 42.817 1.00 0.00 H

ATOM 1050 HB2 PHE A 203 35.413 31.947 42.785 1.00 0.00 H

ATOM 1051 HD1 PHE A 203 32.247 33.278 41.280 1.00 0.00 H

ATOM 1052 HD2 PHE A 203 34.446 29.857 42.568 1.00 0.00 H

ATOM 1053 HE1 PHE A 203 30.285 31.831 40.877 1.00 0.00 H

ATOM 1054 HE2 PHE A 203 32.540 28.436 42.132 1.00 0.00 H

ATOM 1055 HZ PHE A 203 30.435 29.409 41.282 1.00 0.00 H

ATOM 1056 N VAL A 204 36.316 35.199 41.365 1.00 11.90 N

ATOM 1057 CA VAL A 204 37.229 36.141 42.011 1.00 12.20 C

ATOM 1058 C VAL A 204 36.460 36.816 43.140 1.00 10.01 C

ATOM 1059 O VAL A 204 35.437 37.442 42.909 1.00 11.60 O

ATOM 1060 CB VAL A 204 37.802 37.188 41.038 1.00 12.41 C

ATOM 1061 CG1 VAL A 204 38.540 38.304 41.811 1.00 11.80 C

ATOM 1062 CG2 VAL A 204 38.740 36.512 40.051 1.00 13.33 C

ATOM 1063 H VAL A 204 35.411 35.544 41.072 1.00 0.00 H

ATOM 1064 HA VAL A 204 38.071 35.615 42.451 1.00 0.00 H

ATOM 1065 HB VAL A 204 36.990 37.642 40.473 1.00 0.00 H

ATOM 1066 HG11 VAL A 204 39.043 38.974 41.113 1.00 0.00 H

ATOM 1067 HG12 VAL A 204 37.876 38.934 42.404 1.00 0.00 H

ATOM 1068 HG13 VAL A 204 39.304 37.896 42.471 1.00 0.00 H

ATOM 1069 HG21 VAL A 204 39.048 37.219 39.285 1.00 0.00 H

ATOM 1070 HG22 VAL A 204 39.639 36.146 40.545 1.00 0.00 H

ATOM 1071 HG23 VAL A 204 38.264 35.670 39.552 1.00 0.00 H

ATOM 1072 N VAL A 205 36.951 36.659 44.368 1.00 9.67 N

ATOM 1073 CA VAL A 205 36.184 37.112 45.526 1.00 10.37 C

ATOM 1074 C VAL A 205 36.721 38.423 46.067 1.00 11.34 C

ATOM 1075 O VAL A 205 37.902 38.727 45.916 1.00 13.50 O

ATOM 1076 CB VAL A 205 36.162 36.055 46.666 1.00 12.59 C

ATOM 1077 CG1 VAL A 205 35.509 34.765 46.179 1.00 12.95 C

ATOM 1078 CG2 VAL A 205 37.578 35.784 47.205 1.00 14.14 C

ATOM 1079 H VAL A 205 37.817 36.155 44.516 1.00 0.00 H

ATOM 1080 HA VAL A 205 35.141 37.290 45.268 1.00 0.00 H

ATOM 1081 HB VAL A 205 35.558 36.437 47.490 1.00 0.00 H

ATOM 1082 HG11 VAL A 205 35.379 34.045 46.987 1.00 0.00 H

ATOM 1083 HG12 VAL A 205 34.545 34.958 45.712 1.00 0.00 H

ATOM 1084 HG13 VAL A 205 36.119 34.297 45.409 1.00 0.00 H

ATOM 1085 HG21 VAL A 205 37.549 35.003 47.964 1.00 0.00 H

ATOM 1086 HG22 VAL A 205 38.251 35.428 46.430 1.00 0.00 H

ATOM 1087 HG23 VAL A 205 38.031 36.657 47.676 1.00 0.00 H

ATOM 1088 N ASN A 206 35.841 39.213 46.682 1.00 11.64 N

ATOM 1089 CA ASN A 206 36.266 40.396 47.405 1.00 10.51 C

ATOM 1090 C ASN A 206 36.390 40.046 48.880 1.00 10.81 C

ATOM 1091 O ASN A 206 35.396 39.863 49.564 1.00 12.26 O

ATOM 1092 CB ASN A 206 35.263 41.543 47.207 1.00 9.64 C

ATOM 1093 CG ASN A 206 35.704 42.831 47.892 1.00 13.68 C

ATOM 1094 OD1 ASN A 206 36.622 42.829 48.713 1.00 13.67 O

ATOM 1095 ND2 ASN A 206 35.073 43.939 47.538 1.00 14.23 N

ATOM 1096 H ASN A 206 34.867 38.943 46.744 1.00 0.00 H

ATOM 1097 HA ASN A 206 37.229 40.758 47.038 1.00 0.00 H

ATOM 1098 HB3 ASN A 206 34.289 41.266 47.610 1.00 0.00 H

ATOM 1099 HB2 ASN A 206 35.113 41.726 46.145 1.00 0.00 H

ATOM 1100 HD22 ASN A 206 35.330 44.818 47.976 1.00 0.00 H

ATOM 1101 HD21 ASN A 206 34.328 43.911 46.855 1.00 0.00 H

ATOM 1102 N ARG A 207 37.627 39.960 49.350 1.00 9.83 N

ATOM 1103 CA ARG A 207 37.910 39.575 50.730 1.00 11.38 C

ATOM 1104 C ARG A 207 37.187 40.463 51.752 1.00 10.19 C

ATOM 1105 O ARG A 207 36.801 40.002 52.833 1.00 11.95 O

ATOM 1106 CB ARG A 207 39.425 39.595 50.948 1.00 12.91 C

ATOM 1107 CG ARG A 207 40.136 38.359 50.394 1.00 12.63 C

ATOM 1108 CD ARG A 207 41.606 38.633 50.129 1.00 13.57 C

ATOM 1109 NE ARG A 207 42.400 37.401 50.065 1.00 12.34 N

ATOM 1110 CZ ARG A 207 43.556 37.285 49.415 1.00 14.13 C

ATOM 1111 NH1 ARG A 207 44.034 38.299 48.706 1.00 13.99 N

ATOM 1112 NH2 ARG A 207 44.222 36.137 49.454 1.00 15.87 N1+

ATOM 1113 H ARG A 207 38.409 40.139 48.731 1.00 0.00 H

ATOM 1114 HA ARG A 207 37.546 38.560 50.883 1.00 0.00 H

ATOM 1115 HB3 ARG A 207 39.656 39.660 52.013 1.00 0.00 H

ATOM 1116 HB2 ARG A 207 39.838 40.505 50.509 1.00 0.00 H

ATOM 1117 HG3 ARG A 207 39.674 38.057 49.452 1.00 0.00 H

ATOM 1118 HG2 ARG A 207 40.006 37.519 51.078 1.00 0.00 H

ATOM 1119 HD3 ARG A 207 42.028 39.245 50.927 1.00 0.00 H

ATOM 1120 HD2 ARG A 207 41.700 39.206 49.207 1.00 0.00 H

ATOM 1121 HE ARG A 207 42.101 36.636 50.663 1.00 0.00 H

ATOM 1122 HH12 ARG A 207 44.907 38.212 48.207 1.00 0.00 H

ATOM 1123 HH11 ARG A 207 43.539 39.181 48.662 1.00 0.00 H

ATOM 1124 HH22 ARG A 207 45.091 36.032 48.953 1.00 0.00 H

ATOM 1125 HH21 ARG A 207 43.882 35.358 50.004 1.00 0.00 H

ATOM 1126 N HIS A 208 36.984 41.726 51.411 1.00 11.59 N

ATOM 1127 CA HIS A 208 36.302 42.634 52.333 1.00 11.32 C

ATOM 1128 C HIS A 208 34.857 42.240 52.590 1.00 14.99 C

ATOM 1129 O HIS A 208 34.266 42.653 53.586 1.00 13.94 O

ATOM 1130 CB HIS A 208 36.373 44.062 51.813 1.00 12.67 C

ATOM 1131 CG HIS A 208 37.743 44.647 51.872 1.00 12.97 C

ATOM 1132 ND1 HIS A 208 37.999 45.970 51.564 1.00 14.80 N

ATOM 1133 CD2 HIS A 208 38.937 44.097 52.214 1.00 13.48 C

ATOM 1134 CE1 HIS A 208 39.293 46.202 51.706 1.00 15.16 C

ATOM 1135 NE2 HIS A 208 39.882 45.088 52.104 1.00 14.54 N

ATOM 1136 H HIS A 208 37.270 42.069 50.503 1.00 0.00 H

ATOM 1137 HA HIS A 208 36.806 42.598 53.302 1.00 0.00 H

ATOM 1138 HB3 HIS A 208 35.735 44.710 52.416 1.00 0.00 H

ATOM 1139 HB2 HIS A 208 35.990 44.134 50.796 1.00 0.00 H

ATOM 1140 HD1 HIS A 208 37.297 46.646 51.275 1.00 0.00 H

ATOM 1141 HD2 HIS A 208 39.190 43.094 52.525 1.00 0.00 H

ATOM 1142 HE1 HIS A 208 39.786 47.147 51.528 1.00 0.00 H

ATOM 1143 N ASN A 209 34.282 41.448 51.689 1.00 12.09 N

ATOM 1144 CA ASN A 209 32.880 41.073 51.816 1.00 12.57 C

ATOM 1145 C ASN A 209 32.658 39.615 52.214 1.00 10.56 C

ATOM 1146 O ASN A 209 31.520 39.129 52.274 1.00 12.23 O

ATOM 1147 CB ASN A 209 32.123 41.440 50.532 1.00 11.95 C

ATOM 1148 CG ASN A 209 31.805 42.916 50.470 1.00 12.38 C

ATOM 1149 OD1 ASN A 209 31.430 43.507 51.478 1.00 14.54 O

ATOM 1150 ND2 ASN A 209 31.960 43.524 49.298 1.00 14.45 N

ATOM 1151 H ASN A 209 34.809 41.111 50.893 1.00 0.00 H

ATOM 1152 HA ASN A 209 32.389 41.584 52.642 1.00 0.00 H

ATOM 1153 HB3 ASN A 209 31.169 40.917 50.478 1.00 0.00 H

ATOM 1154 HB2 ASN A 209 32.690 41.129 49.652 1.00 0.00 H

ATOM 1155 HD22 ASN A 209 31.727 44.507 49.219 1.00 0.00 H

ATOM 1156 HD21 ASN A 209 32.265 43.010 48.481 1.00 0.00 H

ATOM 1157 N ILE A 210 33.745 38.917 52.538 1.00 9.97 N

ATOM 1158 CA ILE A 210 33.637 37.549 53.030 1.00 11.70 C

ATOM 1159 C ILE A 210 33.266 37.527 54.500 1.00 13.96 C

ATOM 1160 O ILE A 210 33.915 38.187 55.316 1.00 14.46 O

ATOM 1161 CB ILE A 210 34.946 36.768 52.791 1.00 10.79 C

ATOM 1162 CG1 ILE A 210 35.165 36.641 51.285 1.00 12.82 C

ATOM 1163 CG2 ILE A 210 34.881 35.397 53.432 1.00 14.95 C

ATOM 1164 CD1 ILE A 210 36.475 35.933 50.911 1.00 13.63 C

ATOM 1165 H ILE A 210 34.656 39.355 52.512 1.00 0.00 H

ATOM 1166 HA ILE A 210 32.856 37.030 52.469 1.00 0.00 H

ATOM 1167 HB ILE A 210 35.783 37.317 53.227 1.00 0.00 H

ATOM 1168 HG13 ILE A 210 35.188 37.628 50.829 1.00 0.00 H

ATOM 1169 HG12 ILE A 210 34.330 36.115 50.822 1.00 0.00 H

ATOM 1170 HG21 ILE A 210 35.770 34.808 53.215 1.00 0.00 H

ATOM 1171 HG22 ILE A 210 34.837 35.432 54.520 1.00 0.00 H

ATOM 1172 HG23 ILE A 210 34.021 34.827 53.079 1.00 0.00 H

ATOM 1173 HD11 ILE A 210 36.694 36.083 49.856 1.00 0.00 H

ATOM 1174 HD12 ILE A 210 37.320 36.317 51.483 1.00 0.00 H

ATOM 1175 HD13 ILE A 210 36.411 34.859 51.079 1.00 0.00 H

ATOM 1176 N GLU A 211 32.204 36.792 54.821 1.00 11.15 N

ATOM 1177 CA GLU A 211 31.740 36.621 56.204 1.00 13.14 C

ATOM 1178 C GLU A 211 32.355 35.391 56.871 1.00 17.77 C

ATOM 1179 O GLU A 211 32.718 35.432 58.044 1.00 17.51 O

ATOM 1180 CB GLU A 211 30.221 36.489 56.217 1.00 17.81 C

ATOM 1181 CG GLU A 211 29.583 36.705 57.573 1.00 26.10 C

ATOM 1182 CD GLU A 211 29.228 38.166 57.793 1.00 37.17 C

ATOM 1183 OE1 GLU A 211 30.155 38.987 57.962 1.00 34.38 O

ATOM 1184 OE2 GLU A 211 28.019 38.498 57.771 1.00 42.33 O1-

ATOM 1185 H GLU A 211 31.727 36.272 54.094 1.00 0.00 H

ATOM 1186 HA GLU A 211 32.014 37.500 56.790 1.00 0.00 H

ATOM 1187 HB3 GLU A 211 29.929 35.508 55.836 1.00 0.00 H

ATOM 1188 HB2 GLU A 211 29.792 37.192 55.499 1.00 0.00 H

ATOM 1189 HG3 GLU A 211 30.206 36.351 58.394 1.00 0.00 H

ATOM 1190 HG2 GLU A 211 28.660 36.127 57.626 1.00 0.00 H

ATOM 1191 N SER A 212 32.434 34.281 56.139 1.00 13.07 N

ATOM 1192 CA SER A 212 33.044 33.063 56.655 1.00 16.04 C

ATOM 1193 C SER A 212 33.441 32.144 55.504 1.00 14.37 C

ATOM 1194 O SER A 212 33.011 32.330 54.358 1.00 14.41 O

ATOM 1195 CB SER A 212 32.093 32.331 57.608 1.00 16.13 C

ATOM 1196 OG SER A 212 30.978 31.808 56.900 1.00 16.76 O

ATOM 1197 H SER A 212 32.109 34.281 55.182 1.00 0.00 H

ATOM 1198 HA SER A 212 33.959 33.325 57.191 1.00 0.00 H

ATOM 1199 HB3 SER A 212 31.737 32.984 58.405 1.00 0.00 H

ATOM 1200 HB2 SER A 212 32.608 31.503 58.097 1.00 0.00 H

ATOM 1201 HG SER A 212 30.435 31.328 57.507 1.00 0.00 H

ATOM 1202 N ILE A 213 34.290 31.171 55.805 1.00 13.69 N

ATOM 1203 CA ILE A 213 34.740 30.192 54.828 1.00 13.80 C

ATOM 1204 C ILE A 213 34.660 28.830 55.488 1.00 17.53 C

ATOM 1205 O ILE A 213 35.185 28.625 56.586 1.00 19.01 O

ATOM 1206 CB ILE A 213 36.191 30.422 54.366 1.00 13.08 C

ATOM 1207 CG1 ILE A 213 36.320 31.763 53.644 1.00 15.80 C

ATOM 1208 CG2 ILE A 213 36.651 29.276 53.426 1.00 16.64 C

ATOM 1209 CD1 ILE A 213 37.753 32.189 53.418 1.00 16.16 C

ATOM 1210 H ILE A 213 34.613 31.065 56.756 1.00 0.00 H

ATOM 1211 HA ILE A 213 34.092 30.189 53.951 1.00 0.00 H

ATOM 1212 HB ILE A 213 36.844 30.439 55.241 1.00 0.00 H

ATOM 1213 HG13 ILE A 213 35.859 32.556 54.229 1.00 0.00 H

ATOM 1214 HG12 ILE A 213 35.783 31.735 52.696 1.00 0.00 H

ATOM 1215 HG21 ILE A 213 37.636 29.462 53.001 1.00 0.00 H

ATOM 1216 HG22 ILE A 213 36.746 28.320 53.939 1.00 0.00 H

ATOM 1217 HG23 ILE A 213 35.960 29.142 52.592 1.00 0.00 H

ATOM 1218 HD11 ILE A 213 37.789 33.193 52.997 1.00 0.00 H

ATOM 1219 HD12 ILE A 213 38.318 32.204 54.350 1.00 0.00 H

ATOM 1220 HD13 ILE A 213 38.264 31.528 52.723 1.00 0.00 H

ATOM 1221 N ASP A 214 33.970 27.912 54.833 1.00 16.75 N

ATOM 1222 CA ASP A 214 33.943 26.517 55.242 1.00 15.57 C

ATOM 1223 C ASP A 214 35.072 25.861 54.456 1.00 18.74 C

ATOM 1224 O ASP A 214 34.936 25.597 53.267 1.00 15.88 O

ATOM 1225 CB ASP A 214 32.583 25.913 54.883 1.00 17.27 C

ATOM 1226 CG ASP A 214 32.463 24.432 55.235 1.00 19.55 C

ATOM 1227 OD1 ASP A 214 33.491 23.746 55.423 1.00 19.52 O

ATOM 1228 OD2 ASP A 214 31.316 23.943 55.292 1.00 22.29 O1-

ATOM 1229 H ASP A 214 33.548 28.153 53.944 1.00 0.00 H

ATOM 1230 HA ASP A 214 34.109 26.399 56.315 1.00 0.00 H

ATOM 1231 HB3 ASP A 214 32.375 26.026 53.819 1.00 0.00 H

ATOM 1232 HB2 ASP A 214 31.795 26.452 55.410 1.00 0.00 H

ATOM 1233 N SER A 215 36.207 25.622 55.108 1.00 19.97 N

ATOM 1234 CA ASER A 215 37.369 25.076 54.418 0.59 20.14 C

ATOM 1235 CA BSER A 215 37.370 25.072 54.420 0.41 20.15 C

ATOM 1236 C SER A 215 37.200 23.602 54.058 1.00 18.55 C

ATOM 1237 O SER A 215 37.840 23.114 53.131 1.00 23.17 O

ATOM 1238 CB ASER A 215 38.633 25.268 55.262 0.59 22.85 C

ATOM 1239 CB BSER A 215 38.639 25.267 55.256 0.41 22.83 C

ATOM 1240 OG ASER A 215 38.481 24.659 56.529 0.59 25.22 O

ATOM 1241 OG BSER A 215 39.032 26.626 55.259 0.41 24.07 O

ATOM 1242 H SER A 215 36.289 25.848 56.089 1.00 0.00 H

ATOM 1243 HA ASER A 215 37.515 25.627 53.488 0.59 0.00 H

ATOM 1244 HA BSER A 215 37.498 25.628 53.491 0.41 0.00 H

ATOM 1245 HB3ASER A 215 38.828 26.331 55.406 0.59 0.00 H

ATOM 1246 HB3BSER A 215 39.443 24.658 54.843 0.41 0.00 H

ATOM 1247 HB2ASER A 215 39.509 24.852 54.762 0.59 0.00 H

ATOM 1248 HB2BSER A 215 38.453 24.941 56.279 0.41 0.00 H

ATOM 1249 HG ASER A 215 39.245 24.849 57.051 0.59 0.00 H

ATOM 1250 HG BSER A 215 39.827 26.728 55.787 0.41 0.00 H

ATOM 1251 N LYS A 216 36.339 22.901 54.791 1.00 21.22 N

ATOM 1252 CA LYS A 216 36.093 21.490 54.507 1.00 22.25 C

ATOM 1253 C LYS A 216 35.289 21.311 53.222 1.00 24.98 C

ATOM 1254 O LYS A 216 35.705 20.582 52.317 1.00 26.97 O

ATOM 1255 CB LYS A 216 35.376 20.801 55.669 1.00 23.35 C

ATOM 1256 CG LYS A 216 35.241 19.302 55.467 1.00 37.50 C

ATOM 1257 CD LYS A 216 33.997 18.733 56.126 1.00 39.88 C

ATOM 1258 CE LYS A 216 33.811 17.274 55.739 1.00 46.27 C

ATOM 1259 NZ LYS A 216 32.505 16.733 56.202 1.00 57.04 N1+

ATOM 1260 H LYS A 216 35.799 23.356 55.515 1.00 0.00 H

ATOM 1261 HA LYS A 216 37.056 20.990 54.379 1.00 0.00 H

ATOM 1262 HB3 LYS A 216 34.392 21.253 55.802 1.00 0.00 H

ATOM 1263 HB2 LYS A 216 35.908 20.988 56.602 1.00 0.00 H

ATOM 1264 HG3 LYS A 216 36.132 18.805 55.854 1.00 0.00 H

ATOM 1265 HG2 LYS A 216 35.202 19.028 54.415 1.00 0.00 H

ATOM 1266 HD3 LYS A 216 33.124 19.300 55.798 1.00 0.00 H

ATOM 1267 HD2 LYS A 216 34.056 18.841 57.210 1.00 0.00 H

ATOM 1268 HE3 LYS A 216 34.616 16.666 56.153 1.00 0.00 H

ATOM 1269 HE2 LYS A 216 33.847 17.158 54.655 1.00 0.00 H

ATOM 1270 HZ1 LYS A 216 31.764 17.254 55.752 1.00 0.00 H

ATOM 1271 HZ2 LYS A 216 32.431 15.760 55.944 1.00 0.00 H

ATOM 1272 HZ3 LYS A 216 32.429 16.828 57.204 1.00 0.00 H

ATOM 1273 N GLU A 217 34.139 21.976 53.143 1.00 20.60 N

ATOM 1274 CA AGLU A 217 33.278 21.885 51.969 0.54 18.37 C

ATOM 1275 CA BGLU A 217 33.299 21.862 51.959 0.46 18.40 C

ATOM 1276 C GLU A 217 33.697 22.865 50.884 1.00 20.25 C

ATOM 1277 O GLU A 217 33.206 22.801 49.757 1.00 18.90 O

ATOM 1278 CB AGLU A 217 31.810 22.102 52.351 0.54 21.31 C

ATOM 1279 CB BGLU A 217 31.816 21.981 52.321 0.46 21.37 C

ATOM 1280 CG AGLU A 217 31.259 21.045 53.303 0.54 23.27 C

ATOM 1281 CG BGLU A 217 31.341 20.863 53.244 0.46 23.08 C

ATOM 1282 CD AGLU A 217 31.348 19.640 52.740 0.54 24.74 C

ATOM 1283 CD BGLU A 217 29.834 20.710 53.264 0.46 25.32 C

ATOM 1284 OE1AGLU A 217 31.258 19.481 51.505 0.54 25.29 O

ATOM 1285 OE1BGLU A 217 29.251 20.664 54.369 0.46 23.43 O

ATOM 1286 OE2AGLU A 217 31.508 18.691 53.536 0.54 32.25 O1-

ATOM 1287 OE2BGLU A 217 29.234 20.621 52.173 0.46 24.76 O1-

ATOM 1288 H GLU A 217 33.842 22.556 53.921 1.00 0.00 H

ATOM 1289 HA AGLU A 217 33.384 20.902 51.509 0.54 0.00 H

ATOM 1290 HA BGLU A 217 33.453 20.865 51.546 0.46 0.00 H

ATOM 1291 HB3AGLU A 217 31.190 22.119 51.453 0.54 0.00 H

ATOM 1292 HB3BGLU A 217 31.219 21.976 51.409 0.46 0.00 H

ATOM 1293 HB2AGLU A 217 31.688 23.089 52.796 0.54 0.00 H

ATOM 1294 HB2BGLU A 217 31.636 22.945 52.797 0.46 0.00 H

ATOM 1295 HG3AGLU A 217 30.213 21.260 53.522 0.54 0.00 H

ATOM 1296 HG3BGLU A 217 31.699 21.052 54.256 0.46 0.00 H

ATOM 1297 HG2AGLU A 217 31.788 21.085 54.256 0.54 0.00 H

ATOM 1298 HG2BGLU A 217 31.796 19.922 52.937 0.46 0.00 H

ATOM 1299 N ARG A 218 34.611 23.770 51.233 1.00 15.20 N

ATOM 1300 CA ARG A 218 35.129 24.754 50.284 1.00 15.66 C

ATOM 1301 C ARG A 218 34.022 25.688 49.785 1.00 12.79 C

ATOM 1302 O ARG A 218 33.851 25.903 48.590 1.00 15.28 O

ATOM 1303 CB ARG A 218 35.880 24.085 49.123 1.00 17.37 C

ATOM 1304 CG ARG A 218 37.099 23.302 49.593 1.00 18.00 C

ATOM 1305 CD ARG A 218 37.885 22.723 48.426 1.00 22.41 C

ATOM 1306 NE ARG A 218 37.250 21.527 47.883 1.00 28.15 N

ATOM 1307 CZ ARG A 218 36.544 21.500 46.760 1.00 29.69 C

ATOM 1308 NH1 ARG A 218 36.003 20.364 46.345 1.00 30.90 N

ATOM 1309 NH2 ARG A 218 36.381 22.608 46.049 1.00 29.65 N1+

ATOM 1310 H ARG A 218 34.949 23.795 52.186 1.00 0.00 H

ATOM 1311 HA ARG A 218 35.835 25.380 50.828 1.00 0.00 H

ATOM 1312 HB3 ARG A 218 36.242 24.900 48.512 1.00 0.00 H

ATOM 1313 HB2 ARG A 218 35.239 23.471 48.493 1.00 0.00 H

ATOM 1314 HG3 ARG A 218 36.793 22.495 50.260 1.00 0.00 H

ATOM 1315 HG2 ARG A 218 37.747 23.948 50.183 1.00 0.00 H

ATOM 1316 HD3 ARG A 218 38.838 22.387 48.829 1.00 0.00 H

ATOM 1317 HD2 ARG A 218 38.128 23.469 47.680 1.00 0.00 H

ATOM 1318 HE ARG A 218 37.341 20.682 48.430 1.00 0.00 H

ATOM 1319 HH12 ARG A 218 36.120 19.519 46.886 1.00 0.00 H

ATOM 1320 HH11 ARG A 218 35.466 20.328 45.491 1.00 0.00 H

ATOM 1321 HH22 ARG A 218 35.881 22.581 45.174 1.00 0.00 H

ATOM 1322 HH21 ARG A 218 36.759 23.495 46.371 1.00 0.00 H

ATOM 1323 N ILE A 219 33.298 26.258 50.739 1.00 15.96 N

ATOM 1324 CA ILE A 219 32.247 27.227 50.458 1.00 13.65 C

ATOM 1325 C ILE A 219 32.629 28.567 51.086 1.00 12.87 C

ATOM 1326 O ILE A 219 33.017 28.622 52.261 1.00 13.64 O

ATOM 1327 CB ILE A 219 30.898 26.733 51.017 1.00 14.60 C

ATOM 1328 CG1 ILE A 219 30.550 25.380 50.403 1.00 15.30 C

ATOM 1329 CG2 ILE A 219 29.776 27.732 50.708 1.00 13.02 C

ATOM 1330 CD1 ILE A 219 29.356 24.697 51.048 1.00 17.07 C

ATOM 1331 H ILE A 219 33.514 26.056 51.706 1.00 0.00 H

ATOM 1332 HA ILE A 219 32.122 27.379 49.385 1.00 0.00 H

ATOM 1333 HB ILE A 219 30.973 26.619 52.099 1.00 0.00 H

ATOM 1334 HG13 ILE A 219 31.376 24.682 50.512 1.00 0.00 H

ATOM 1335 HG12 ILE A 219 30.382 25.483 49.331 1.00 0.00 H

ATOM 1336 HG21 ILE A 219 28.805 27.386 51.056 1.00 0.00 H

ATOM 1337 HG22 ILE A 219 29.934 28.698 51.187 1.00 0.00 H

ATOM 1338 HG23 ILE A 219 29.691 27.906 49.635 1.00 0.00 H

ATOM 1339 HD11 ILE A 219 29.240 23.685 50.659 1.00 0.00 H

ATOM 1340 HD12 ILE A 219 29.481 24.619 52.129 1.00 0.00 H

ATOM 1341 HD13 ILE A 219 28.421 25.220 50.851 1.00 0.00 H

ATOM 1342 N VAL A 220 32.525 29.634 50.299 1.00 11.30 N

ATOM 1343 CA VAL A 220 32.841 30.987 50.731 1.00 11.33 C

ATOM 1344 C VAL A 220 31.529 31.734 50.879 1.00 12.36 C

ATOM 1345 O VAL A 220 30.749 31.796 49.935 1.00 12.66 O

ATOM 1346 CB VAL A 220 33.705 31.716 49.689 1.00 11.65 C

ATOM 1347 CG1 VAL A 220 34.063 33.118 50.184 1.00 13.44 C

ATOM 1348 CG2 VAL A 220 34.975 30.909 49.391 1.00 15.54 C

ATOM 1349 H VAL A 220 32.186 29.513 49.352 1.00 0.00 H

ATOM 1350 HA VAL A 220 33.372 30.989 51.685 1.00 0.00 H

ATOM 1351 HB VAL A 220 33.153 31.813 48.752 1.00 0.00 H

ATOM 1352 HG11 VAL A 220 34.733 33.614 49.482 1.00 0.00 H

ATOM 1353 HG12 VAL A 220 33.194 33.767 50.290 1.00 0.00 H

ATOM 1354 HG13 VAL A 220 34.569 33.086 51.149 1.00 0.00 H

ATOM 1355 HG21 VAL A 220 35.601 31.434 48.669 1.00 0.00 H

ATOM 1356 HG22 VAL A 220 35.567 30.745 50.292 1.00 0.00 H

ATOM 1357 HG23 VAL A 220 34.751 29.935 48.955 1.00 0.00 H

ATOM 1358 N TYR A 221 31.276 32.290 52.058 1.00 10.94 N

ATOM 1359 CA TYR A 221 30.025 33.004 52.343 1.00 12.21 C

ATOM 1360 C TYR A 221 30.251 34.504 52.409 1.00 12.74 C

ATOM 1361 O TYR A 221 31.203 34.973 53.035 1.00 12.92 O

ATOM 1362 CB TYR A 221 29.418 32.536 53.678 1.00 12.25 C

ATOM 1363 CG TYR A 221 28.978 31.089 53.688 1.00 14.58 C

ATOM 1364 CD1 TYR A 221 29.799 30.101 54.203 1.00 14.72 C

ATOM 1365 CD2 TYR A 221 27.739 30.711 53.182 1.00 13.70 C

ATOM 1366 CE1 TYR A 221 29.408 28.765 54.205 1.00 15.17 C

ATOM 1367 CE2 TYR A 221 27.343 29.381 53.174 1.00 12.46 C

ATOM 1368 CZ TYR A 221 28.180 28.412 53.693 1.00 14.20 C

ATOM 1369 OH TYR A 221 27.796 27.085 53.702 1.00 18.15 O

ATOM 1370 H TYR A 221 31.959 32.221 52.803 1.00 0.00 H

ATOM 1371 HA TYR A 221 29.277 32.807 51.573 1.00 0.00 H

ATOM 1372 HB3 TYR A 221 28.547 33.146 53.926 1.00 0.00 H

ATOM 1373 HB2 TYR A 221 30.130 32.698 54.488 1.00 0.00 H

ATOM 1374 HD1 TYR A 221 30.771 30.362 54.596 1.00 0.00 H

ATOM 1375 HD2 TYR A 221 27.077 31.460 52.771 1.00 0.00 H

ATOM 1376 HE1 TYR A 221 30.072 28.014 54.606 1.00 0.00 H

ATOM 1377 HE2 TYR A 221 26.382 29.107 52.766 1.00 0.00 H

ATOM 1378 HH TYR A 221 28.469 26.517 54.045 1.00 0.00 H

ATOM 1379 N PHE A 222 29.339 35.254 51.799 1.00 12.06 N

ATOM 1380 CA PHE A 222 29.448 36.704 51.706 1.00 12.88 C

ATOM 1381 C PHE A 222 28.449 37.422 52.585 1.00 12.26 C

ATOM 1382 O PHE A 222 27.468 36.822 53.046 1.00 12.28 O

ATOM 1383 CB PHE A 222 29.322 37.133 50.239 1.00 11.56 C

ATOM 1384 CG PHE A 222 30.427 36.588 49.399 1.00 10.53 C

ATOM 1385 CD1 PHE A 222 31.606 37.297 49.263 1.00 10.69 C

ATOM 1386 CD2 PHE A 222 30.337 35.313 48.852 1.00 12.50 C

ATOM 1387 CE1 PHE A 222 32.663 36.763 48.546 1.00 11.43 C

ATOM 1388 CE2 PHE A 222 31.380 34.776 48.129 1.00 12.13 C

ATOM 1389 CZ PHE A 222 32.561 35.515 47.984 1.00 10.34 C

ATOM 1390 H PHE A 222 28.565 34.808 51.321 1.00 0.00 H

ATOM 1391 HA PHE A 222 30.433 37.013 52.043 1.00 0.00 H

ATOM 1392 HB3 PHE A 222 29.335 38.220 50.157 1.00 0.00 H

ATOM 1393 HB2 PHE A 222 28.363 36.812 49.827 1.00 0.00 H

ATOM 1394 HD1 PHE A 222 31.708 38.276 49.706 1.00 0.00 H

ATOM 1395 HD2 PHE A 222 29.438 34.737 48.972 1.00 0.00 H

ATOM 1396 HE1 PHE A 222 33.578 37.328 48.446 1.00 0.00 H

ATOM 1397 HE2 PHE A 222 31.293 33.789 47.704 1.00 0.00 H

ATOM 1398 HZ PHE A 222 33.385 35.082 47.459 1.00 0.00 H

ATOM 1399 N LYS A 223 28.706 38.704 52.816 1.00 11.98 N

ATOM 1400 CA LYS A 223 27.840 39.490 53.707 1.00 14.74 C

ATOM 1401 C LYS A 223 26.372 39.497 53.291 1.00 16.10 C

ATOM 1402 O LYS A 223 25.485 39.630 54.139 1.00 17.35 O

ATOM 1403 CB LYS A 223 28.336 40.928 53.798 1.00 19.18 C

ATOM 1404 CG LYS A 223 29.656 41.097 54.506 1.00 22.16 C

ATOM 1405 CD LYS A 223 29.941 42.582 54.641 1.00 27.65 C

ATOM 1406 CE LYS A 223 31.284 42.860 55.270 1.00 33.22 C

ATOM 1407 NZ LYS A 223 31.488 44.338 55.367 1.00 38.91 N1+

ATOM 1408 H LYS A 223 29.543 39.130 52.440 1.00 0.00 H

ATOM 1409 HA LYS A 223 27.889 39.044 54.703 1.00 0.00 H

ATOM 1410 HB3 LYS A 223 27.591 41.525 54.328 1.00 0.00 H

ATOM 1411 HB2 LYS A 223 28.401 41.355 52.795 1.00 0.00 H

ATOM 1412 HG3 LYS A 223 30.456 40.608 53.959 1.00 0.00 H

ATOM 1413 HG2 LYS A 223 29.618 40.632 55.493 1.00 0.00 H

ATOM 1414 HD3 LYS A 223 29.157 43.052 55.237 1.00 0.00 H

ATOM 1415 HD2 LYS A 223 29.903 43.050 53.656 1.00 0.00 H

ATOM 1416 HE3 LYS A 223 32.084 42.408 54.694 1.00 0.00 H

ATOM 1417 HE2 LYS A 223 31.331 42.431 56.271 1.00 0.00 H

ATOM 1418 HZ1 LYS A 223 31.466 44.737 54.439 1.00 0.00 H

ATOM 1419 HZ2 LYS A 223 30.756 44.747 55.930 1.00 0.00 H

ATOM 1420 HZ3 LYS A 223 32.386 44.525 55.789 1.00 0.00 H

ATOM 1421 N ASN A 224 26.107 39.360 51.996 1.00 13.60 N

ATOM 1422 CA ASN A 224 24.732 39.386 51.502 1.00 12.36 C

ATOM 1423 C ASN A 224 24.067 38.012 51.421 1.00 13.15 C

ATOM 1424 O ASN A 224 23.021 37.863 50.778 1.00 14.36 O

ATOM 1425 CB ASN A 224 24.651 40.082 50.143 1.00 12.92 C

ATOM 1426 CG ASN A 224 25.204 39.223 49.015 1.00 12.45 C

ATOM 1427 OD1 ASN A 224 25.882 38.212 49.258 1.00 13.83 O

ATOM 1428 ND2 ASN A 224 24.895 39.606 47.777 1.00 13.29 N

ATOM 1429 H ASN A 224 26.860 39.252 51.331 1.00 0.00 H

ATOM 1430 HA ASN A 224 24.109 39.987 52.167 1.00 0.00 H

ATOM 1431 HB3 ASN A 224 25.215 41.013 50.168 1.00 0.00 H

ATOM 1432 HB2 ASN A 224 23.618 40.351 49.915 1.00 0.00 H

ATOM 1433 HD22 ASN A 224 25.208 39.054 46.983 1.00 0.00 H

ATOM 1434 HD21 ASN A 224 24.316 40.423 47.627 1.00 0.00 H

ATOM 1435 N LYS A 225 24.700 37.018 52.037 1.00 13.41 N

ATOM 1436 CA LYS A 225 24.221 35.633 52.120 1.00 13.33 C

ATOM 1437 C LYS A 225 24.479 34.794 50.870 1.00 13.77 C

ATOM 1438 O LYS A 225 24.206 33.595 50.867 1.00 13.36 O

ATOM 1439 CB LYS A 225 22.745 35.523 52.542 1.00 14.29 C

ATOM 1440 CG LYS A 225 22.374 36.327 53.787 1.00 16.82 C

ATOM 1441 CD LYS A 225 23.281 36.032 54.953 1.00 15.04 C

ATOM 1442 CE LYS A 225 22.804 36.812 56.180 1.00 22.23 C

ATOM 1443 NZ LYS A 225 23.422 36.278 57.422 1.00 21.35 N1+

ATOM 1444 H LYS A 225 25.576 37.227 52.499 1.00 0.00 H

ATOM 1445 HA LYS A 225 24.836 35.162 52.887 1.00 0.00 H

ATOM 1446 HB3 LYS A 225 22.525 34.475 52.751 1.00 0.00 H

ATOM 1447 HB2 LYS A 225 22.072 35.785 51.725 1.00 0.00 H

ATOM 1448 HG3 LYS A 225 21.342 36.089 54.050 1.00 0.00 H

ATOM 1449 HG2 LYS A 225 22.384 37.396 53.573 1.00 0.00 H

ATOM 1450 HD3 LYS A 225 24.316 36.306 54.747 1.00 0.00 H

ATOM 1451 HD2 LYS A 225 23.265 34.960 55.158 1.00 0.00 H

ATOM 1452 HE3 LYS A 225 21.722 36.732 56.293 1.00 0.00 H

ATOM 1453 HE2 LYS A 225 23.041 37.872 56.078 1.00 0.00 H

ATOM 1454 HZ1 LYS A 225 24.427 36.370 57.363 1.00 0.00 H

ATOM 1455 HZ2 LYS A 225 23.086 36.802 58.218 1.00 0.00 H

ATOM 1456 HZ3 LYS A 225 23.178 35.304 57.532 1.00 0.00 H

ATOM 1457 N GLU A 226 25.018 35.401 49.817 1.00 11.62 N

ATOM 1458 CA GLU A 226 25.445 34.601 48.668 1.00 11.00 C

ATOM 1459 C GLU A 226 26.663 33.772 49.056 1.00 11.25 C

ATOM 1460 O GLU A 226 27.326 34.057 50.050 1.00 12.46 O

ATOM 1461 CB GLU A 226 25.791 35.476 47.468 1.00 11.79 C

ATOM 1462 CG GLU A 226 24.586 36.086 46.789 1.00 12.00 C

ATOM 1463 CD GLU A 226 24.986 36.758 45.501 1.00 13.86 C

ATOM 1464 OE1 GLU A 226 25.547 37.860 45.576 1.00 14.77 O

ATOM 1465 OE2 GLU A 226 24.770 36.157 44.425 1.00 15.37 O1-

ATOM 1466 H GLU A 226 25.228 36.391 49.838 1.00 0.00 H

ATOM 1467 HA GLU A 226 24.645 33.917 48.375 1.00 0.00 H

ATOM 1468 HB3 GLU A 226 26.304 34.863 46.724 1.00 0.00 H

ATOM 1469 HB2 GLU A 226 26.509 36.247 47.751 1.00 0.00 H

ATOM 1470 HG3 GLU A 226 24.097 36.808 47.443 1.00 0.00 H

ATOM 1471 HG2 GLU A 226 23.846 35.316 46.568 1.00 0.00 H

ATOM 1472 N HIS A 227 26.932 32.727 48.288 1.00 12.13 N

ATOM 1473 CA HIS A 227 28.155 31.958 48.459 1.00 11.26 C

ATOM 1474 C HIS A 227 28.791 31.717 47.105 1.00 12.31 C

ATOM 1475 O HIS A 227 28.138 31.864 46.070 1.00 13.44 O

ATOM 1476 CB HIS A 227 27.857 30.605 49.104 1.00 10.88 C

ATOM 1477 CG HIS A 227 27.195 29.645 48.170 1.00 10.70 C

ATOM 1478 ND1 HIS A 227 27.681 28.573 47.499 1.00 13.08 N

ATOM 1479 CD2 HIS A 227 25.866 29.752 47.818 1.00 13.12 C

ATOM 1480 CE1 HIS A 227 26.647 28.058 46.754 1.00 13.62 C

ATOM 1481 NE2 HIS A 227 25.563 28.787 46.964 1.00 13.47 N

ATOM 1482 H HIS A 227 26.360 32.534 47.478 1.00 0.00 H

ATOM 1483 HA HIS A 227 28.840 32.550 49.045 1.00 0.00 H

ATOM 1484 HB3 HIS A 227 27.232 30.737 49.988 1.00 0.00 H

ATOM 1485 HB2 HIS A 227 28.778 30.145 49.458 1.00 0.00 H

ATOM 1486 HD2 HIS A 227 25.125 30.479 48.116 1.00 0.00 H

ATOM 1487 HE1 HIS A 227 26.762 27.647 45.787 1.00 0.00 H

ATOM 1488 HE2 HIS A 227 24.706 28.738 46.429 1.00 0.00 H

ATOM 1489 N CYS A 228 30.069 31.347 47.115 1.00 11.76 N

ATOM 1490 CA CYS A 228 30.678 30.716 45.953 1.00 11.05 C

ATOM 1491 C CYS A 228 31.577 29.590 46.441 1.00 11.09 C

ATOM 1492 O CYS A 228 31.573 29.257 47.622 1.00 12.87 O

ATOM 1493 CB CYS A 228 31.430 31.719 45.072 1.00 12.11 C

ATOM 1494 SG CYS A 228 32.973 32.388 45.773 1.00 11.85 S

ATOM 1495 H CYS A 228 30.550 31.271 48.002 1.00 0.00 H

ATOM 1496 HA CYS A 228 29.909 30.234 45.344 1.00 0.00 H

ATOM 1497 HB3 CYS A 228 30.778 32.558 44.832 1.00 0.00 H

ATOM 1498 HB2 CYS A 228 31.674 31.255 44.120 1.00 0.00 H

ATOM 1499 HG CYS A 228 33.611 31.217 45.858 1.00 0.00 H

ATOM 1500 N TYR A 229 32.313 28.971 45.524 1.00 12.27 N

ATOM 1501 CA TYR A 229 33.156 27.838 45.878 1.00 12.89 C

ATOM 1502 C TYR A 229 34.628 28.199 45.747 1.00 11.87 C

ATOM 1503 O TYR A 229 34.989 29.185 45.099 1.00 12.31 O

ATOM 1504 CB TYR A 229 32.806 26.623 45.004 1.00 15.49 C

ATOM 1505 CG TYR A 229 31.332 26.332 45.041 1.00 13.55 C

ATOM 1506 CD1 TYR A 229 30.717 25.996 46.227 1.00 15.47 C

ATOM 1507 CD2 TYR A 229 30.544 26.448 43.906 1.00 17.95 C

ATOM 1508 CE1 TYR A 229 29.360 25.748 46.294 1.00 15.06 C

ATOM 1509 CE2 TYR A 229 29.181 26.220 43.963 1.00 16.73 C

ATOM 1510 CZ TYR A 229 28.594 25.869 45.166 1.00 15.71 C

ATOM 1511 OH TYR A 229 27.242 25.627 45.271 1.00 15.75 O

ATOM 1512 H TYR A 229 32.288 29.275 44.561 1.00 0.00 H

ATOM 1513 HA TYR A 229 33.009 27.537 46.913 1.00 0.00 H

ATOM 1514 HB3 TYR A 229 33.349 25.740 45.342 1.00 0.00 H

ATOM 1515 HB2 TYR A 229 33.115 26.798 43.972 1.00 0.00 H

ATOM 1516 HD1 TYR A 229 31.305 25.912 47.129 1.00 0.00 H

ATOM 1517 HD2 TYR A 229 30.990 26.728 42.963 1.00 0.00 H

ATOM 1518 HE1 TYR A 229 28.910 25.480 47.239 1.00 0.00 H

ATOM 1519 HE2 TYR A 229 28.578 26.318 43.072 1.00 0.00 H

ATOM 1520 HH TYR A 229 26.969 25.413 46.150 1.00 0.00 H

ATOM 1521 N ALA A 230 35.473 27.411 46.408 1.00 13.80 N

ATOM 1522 CA ALA A 230 36.908 27.579 46.318 1.00 14.18 C

ATOM 1523 C ALA A 230 37.502 26.233 45.947 1.00 13.57 C

ATOM 1524 O ALA A 230 36.983 25.207 46.348 1.00 17.22 O

ATOM 1525 CB ALA A 230 37.465 28.036 47.669 1.00 14.70 C

ATOM 1526 H ALA A 230 35.115 26.643 46.961 1.00 0.00 H

ATOM 1527 HA ALA A 230 37.185 28.310 45.565 1.00 0.00 H

ATOM 1528 HB1 ALA A 230 38.544 28.182 47.615 1.00 0.00 H

ATOM 1529 HB2 ALA A 230 37.012 28.982 47.965 1.00 0.00 H

ATOM 1530 HB3 ALA A 230 37.254 27.313 48.457 1.00 0.00 H

ATOM 1531 N SER A 231 38.578 26.247 45.169 1.00 15.79 N

ATOM 1532 CA SER A 231 39.279 25.020 44.830 1.00 17.03 C

ATOM 1533 C SER A 231 40.071 24.515 46.023 1.00 16.73 C

ATOM 1534 O SER A 231 40.355 25.270 46.946 1.00 17.18 O

ATOM 1535 CB SER A 231 40.228 25.283 43.670 1.00 17.85 C

ATOM 1536 OG SER A 231 41.212 26.230 44.044 1.00 17.93 O

ATOM 1537 H SER A 231 38.958 27.135 44.867 1.00 0.00 H

ATOM 1538 HA SER A 231 38.560 24.257 44.522 1.00 0.00 H

ATOM 1539 HB3 SER A 231 39.682 25.660 42.804 1.00 0.00 H

ATOM 1540 HB2 SER A 231 40.725 24.365 43.354 1.00 0.00 H

ATOM 1541 HG SER A 231 40.782 27.052 44.230 1.00 0.00 H

ATOM 1542 N VAL A 232 40.436 23.237 45.996 1.00 17.14 N

ATOM 1543 CA VAL A 232 41.290 22.658 47.025 1.00 20.69 C

ATOM 1544 C VAL A 232 42.601 23.438 47.088 1.00 19.40 C

ATOM 1545 O VAL A 232 43.136 23.709 48.167 1.00 20.31 O

ATOM 1546 CB VAL A 232 41.578 21.172 46.710 1.00 22.00 C

ATOM 1547 CG1 VAL A 232 42.740 20.674 47.538 1.00 28.13 C

ATOM 1548 CG2 VAL A 232 40.334 20.327 46.950 1.00 26.12 C

ATOM 1549 H VAL A 232 40.162 22.652 45.220 1.00 0.00 H

ATOM 1550 HA VAL A 232 40.816 22.749 48.001 1.00 0.00 H

ATOM 1551 HB VAL A 232 41.855 21.071 45.659 1.00 0.00 H

ATOM 1552 HG11 VAL A 232 42.822 19.589 47.460 1.00 0.00 H

ATOM 1553 HG12 VAL A 232 43.705 21.060 47.209 1.00 0.00 H

ATOM 1554 HG13 VAL A 232 42.614 20.905 48.597 1.00 0.00 H

ATOM 1555 HG21 VAL A 232 40.517 19.281 46.701 1.00 0.00 H

ATOM 1556 HG22 VAL A 232 40.021 20.366 47.994 1.00 0.00 H

ATOM 1557 HG23 VAL A 232 39.498 20.661 46.335 1.00 0.00 H

ATOM 1558 N ARG A 233 43.091 23.810 45.915 1.00 19.61 N

ATOM 1559 CA ARG A 233 44.316 24.589 45.756 1.00 22.22 C

ATOM 1560 C ARG A 233 44.312 25.910 46.528 1.00 23.55 C

ATOM 1561 O ARG A 233 45.300 26.280 47.176 1.00 22.40 O

ATOM 1562 CB ARG A 233 44.508 24.886 44.271 1.00 23.33 C

ATOM 1563 CG ARG A 233 45.727 25.689 43.920 1.00 25.37 C

ATOM 1564 CD ARG A 233 45.920 25.655 42.426 1.00 25.99 C

ATOM 1565 NE ARG A 233 46.027 24.274 41.955 1.00 25.66 N

ATOM 1566 CZ ARG A 233 45.798 23.891 40.704 1.00 26.21 C

ATOM 1567 NH1 ARG A 233 45.454 24.789 39.792 1.00 26.87 N

ATOM 1568 NH2 ARG A 233 45.918 22.608 40.369 1.00 30.26 N1+

ATOM 1569 H ARG A 233 42.583 23.559 45.080 1.00 0.00 H

ATOM 1570 HA ARG A 233 45.152 23.984 46.113 1.00 0.00 H

ATOM 1571 HB3 ARG A 233 43.634 25.397 43.871 1.00 0.00 H

ATOM 1572 HB2 ARG A 233 44.509 23.908 43.792 1.00 0.00 H

ATOM 1573 HG3 ARG A 233 46.608 25.279 44.416 1.00 0.00 H

ATOM 1574 HG2 ARG A 233 45.632 26.724 44.249 1.00 0.00 H

ATOM 1575 HD3 ARG A 233 46.820 26.202 42.143 1.00 0.00 H

ATOM 1576 HD2 ARG A 233 45.075 26.155 41.950 1.00 0.00 H

ATOM 1577 HE ARG A 233 46.338 23.589 42.629 1.00 0.00 H

ATOM 1578 HH12 ARG A 233 45.376 25.763 40.045 1.00 0.00 H

ATOM 1579 HH11 ARG A 233 45.277 24.509 38.838 1.00 0.00 H

ATOM 1580 HH22 ARG A 233 45.746 22.310 39.419 1.00 0.00 H

ATOM 1581 HH21 ARG A 233 46.186 21.921 41.059 1.00 0.00 H

ATOM 1582 N ASN A 234 43.200 26.630 46.451 1.00 20.59 N

ATOM 1583 CA ASN A 234 43.191 28.018 46.904 1.00 18.06 C

ATOM 1584 C ASN A 234 42.391 28.330 48.155 1.00 14.98 C

ATOM 1585 O ASN A 234 42.472 29.451 48.658 1.00 17.20 O

ATOM 1586 CB ASN A 234 42.729 28.943 45.772 1.00 20.09 C

ATOM 1587 CG ASN A 234 43.801 29.146 44.721 1.00 22.92 C

ATOM 1588 OD1 ASN A 234 44.968 29.331 45.049 1.00 27.96 O

ATOM 1589 ND2 ASN A 234 43.409 29.111 43.456 1.00 31.31 N

ATOM 1590 H ASN A 234 42.406 26.279 45.932 1.00 0.00 H

ATOM 1591 HA ASN A 234 44.186 28.352 47.201 1.00 0.00 H

ATOM 1592 HB3 ASN A 234 42.494 29.939 46.146 1.00 0.00 H

ATOM 1593 HB2 ASN A 234 41.813 28.561 45.323 1.00 0.00 H

ATOM 1594 HD22 ASN A 234 44.085 29.267 42.723 1.00 0.00 H

ATOM 1595 HD21 ASN A 234 42.437 28.962 43.228 1.00 0.00 H

ATOM 1596 N VAL A 235 41.634 27.369 48.668 1.00 16.29 N

ATOM 1597 CA VAL A 235 40.696 27.670 49.746 1.00 13.63 C

ATOM 1598 C VAL A 235 41.373 28.241 50.997 1.00 20.54 C

ATOM 1599 O VAL A 235 40.872 29.188 51.601 1.00 17.17 O

ATOM 1600 CB VAL A 235 39.778 26.468 50.084 1.00 17.42 C

ATOM 1601 CG1 VAL A 235 40.592 25.275 50.600 1.00 18.96 C

ATOM 1602 CG2 VAL A 235 38.703 26.876 51.084 1.00 18.71 C

ATOM 1603 H VAL A 235 41.593 26.461 48.223 1.00 0.00 H

ATOM 1604 HA VAL A 235 40.047 28.462 49.369 1.00 0.00 H

ATOM 1605 HB VAL A 235 39.276 26.161 49.167 1.00 0.00 H

ATOM 1606 HG11 VAL A 235 39.981 24.373 50.620 1.00 0.00 H

ATOM 1607 HG12 VAL A 235 41.458 25.056 49.975 1.00 0.00 H

ATOM 1608 HG13 VAL A 235 40.931 25.438 51.620 1.00 0.00 H

ATOM 1609 HG21 VAL A 235 37.979 26.074 51.211 1.00 0.00 H

ATOM 1610 HG22 VAL A 235 39.106 27.093 52.073 1.00 0.00 H

ATOM 1611 HG23 VAL A 235 38.154 27.756 50.747 1.00 0.00 H

ATOM 1612 N LYS A 236 42.536 27.704 51.349 1.00 20.35 N

ATOM 1613 CA LYS A 236 43.208 28.144 52.567 1.00 18.29 C

ATOM 1614 C LYS A 236 43.991 29.436 52.371 1.00 20.06 C

ATOM 1615 O LYS A 236 44.480 30.019 53.339 1.00 21.10 O

ATOM 1616 CB LYS A 236 44.119 27.037 53.113 1.00 23.13 C

ATOM 1617 CG LYS A 236 43.362 25.785 53.521 1.00 29.68 C

ATOM 1618 CD LYS A 236 44.293 24.699 54.045 1.00 34.36 C

ATOM 1619 CE LYS A 236 43.489 23.515 54.573 1.00 48.61 C

ATOM 1620 NZ LYS A 236 44.348 22.468 55.196 1.00 49.49 N1+

ATOM 1621 H LYS A 236 42.931 26.947 50.810 1.00 0.00 H

ATOM 1622 HA LYS A 236 42.466 28.347 53.343 1.00 0.00 H

ATOM 1623 HB3 LYS A 236 44.669 27.406 53.980 1.00 0.00 H

ATOM 1624 HB2 LYS A 236 44.871 26.781 52.365 1.00 0.00 H

ATOM 1625 HG3 LYS A 236 42.822 25.382 52.668 1.00 0.00 H

ATOM 1626 HG2 LYS A 236 42.618 26.041 54.277 1.00 0.00 H

ATOM 1627 HD3 LYS A 236 44.910 25.105 54.848 1.00 0.00 H

ATOM 1628 HD2 LYS A 236 44.974 24.374 53.257 1.00 0.00 H

ATOM 1629 HE3 LYS A 236 42.914 23.060 53.766 1.00 0.00 H

ATOM 1630 HE2 LYS A 236 42.771 23.850 55.323 1.00 0.00 H

ATOM 1631 HZ1 LYS A 236 44.858 22.866 55.972 1.00 0.00 H

ATOM 1632 HZ2 LYS A 236 43.769 21.711 55.530 1.00 0.00 H

ATOM 1633 HZ3 LYS A 236 45.001 22.114 54.512 1.00 0.00 H

ATOM 1634 N LYS A 237 44.095 29.895 51.129 1.00 17.36 N

ATOM 1635 CA LYS A 237 44.835 31.112 50.819 1.00 19.13 C

ATOM 1636 C LYS A 237 43.931 32.342 50.768 1.00 18.54 C

ATOM 1637 O LYS A 237 44.419 33.469 50.714 1.00 18.69 O

ATOM 1638 CB LYS A 237 45.583 30.963 49.493 1.00 23.21 C

ATOM 1639 CG LYS A 237 46.571 29.793 49.472 1.00 30.71 C

ATOM 1640 CD LYS A 237 47.172 29.594 48.085 1.00 38.41 C

ATOM 1641 CE LYS A 237 48.214 28.476 48.076 1.00 41.76 C

ATOM 1642 NZ LYS A 237 48.594 28.081 46.684 1.00 40.77 N1+

ATOM 1643 H LYS A 237 43.646 29.398 50.371 1.00 0.00 H

ATOM 1644 HA LYS A 237 45.592 31.311 51.581 1.00 0.00 H

ATOM 1645 HB3 LYS A 237 46.116 31.885 49.253 1.00 0.00 H

ATOM 1646 HB2 LYS A 237 44.855 30.816 48.695 1.00 0.00 H

ATOM 1647 HG3 LYS A 237 46.077 28.868 49.772 1.00 0.00 H

ATOM 1648 HG2 LYS A 237 47.362 29.973 50.201 1.00 0.00 H

ATOM 1649 HD3 LYS A 237 47.624 30.522 47.731 1.00 0.00 H

ATOM 1650 HD2 LYS A 237 46.371 29.353 47.386 1.00 0.00 H

ATOM 1651 HE3 LYS A 237 47.818 27.590 48.574 1.00 0.00 H

ATOM 1652 HE2 LYS A 237 49.107 28.780 48.624 1.00 0.00 H

ATOM 1653 HZ1 LYS A 237 47.770 27.763 46.193 1.00 0.00 H

ATOM 1654 HZ2 LYS A 237 48.988 28.877 46.202 1.00 0.00 H

ATOM 1655 HZ3 LYS A 237 49.275 27.336 46.718 1.00 0.00 H

ATOM 1656 N ILE A 238 42.617 32.127 50.774 1.00 15.80 N

ATOM 1657 CA ILE A 238 41.683 33.246 50.700 1.00 15.31 C

ATOM 1658 C ILE A 238 41.836 34.097 51.951 1.00 16.74 C

ATOM 1659 O ILE A 238 41.930 35.323 51.864 1.00 15.29 O

ATOM 1660 CB ILE A 238 40.227 32.771 50.642 1.00 11.92 C

ATOM 1661 CG1 ILE A 238 40.002 31.895 49.409 1.00 15.42 C

ATOM 1662 CG2 ILE A 238 39.307 33.967 50.576 1.00 17.48 C

ATOM 1663 CD1 ILE A 238 38.687 31.121 49.464 1.00 14.61 C

ATOM 1664 H ILE A 238 42.256 31.184 50.819 1.00 0.00 H

ATOM 1665 HA ILE A 238 41.911 33.851 49.820 1.00 0.00 H

ATOM 1666 HB ILE A 238 40.010 32.180 51.531 1.00 0.00 H

ATOM 1667 HG13 ILE A 238 40.791 31.156 49.316 1.00 0.00 H

ATOM 1668 HG12 ILE A 238 40.035 32.493 48.503 1.00 0.00 H

ATOM 1669 HG21 ILE A 238 38.277 33.664 50.401 1.00 0.00 H

ATOM 1670 HG22 ILE A 238 39.280 34.543 51.501 1.00 0.00 H

ATOM 1671 HG23 ILE A 238 39.582 34.645 49.767 1.00 0.00 H

ATOM 1672 HD11 ILE A 238 38.646 30.391 48.661 1.00 0.00 H

ATOM 1673 HD12 ILE A 238 38.582 30.573 50.401 1.00 0.00 H

ATOM 1674 HD13 ILE A 238 37.824 31.774 49.353 1.00 0.00 H

ATOM 1675 N NMA A 238A 41.877 33.498 53.193 1.00 0.00 N

ATOM 1676 CA NMA A 238A 42.031 34.312 54.390 1.00 0.00 C

ATOM 1677 H NMA A 238A 41.813 32.494 53.281 1.00 0.00 H

ATOM 1678 1HA NMA A 238A 43.029 34.749 54.434 1.00 0.00 H

ATOM 1679 2HA NMA A 238A 41.293 35.116 54.428 1.00 0.00 H

ATOM 1680 3HA NMA A 238A 41.894 33.692 55.276 1.00 0.00 H

TER 1681 NMA A 238A

ATOM 1682 CH3 ACE B 138 16.312 34.262 39.228 1.00 0.00 C

ATOM 1683 C ACE B 138 15.825 34.039 40.660 1.00 0.00 C

ATOM 1684 O ACE B 138 15.847 32.909 41.143 1.00 0.00 O

ATOM 1685 1H ACE B 138 16.149 33.368 38.626 1.00 0.00 H

ATOM 1686 2H ACE B 138 15.778 35.090 38.763 1.00 0.00 H

ATOM 1687 3H ACE B 138 17.378 34.490 39.225 1.00 0.00 H

ATOM 1688 N SER B 139 15.362 35.101 41.410 1.00 43.53 N

ATOM 1689 CA SER B 139 14.898 34.882 42.777 1.00 38.55 C

ATOM 1690 C SER B 139 13.727 33.903 42.802 1.00 39.35 C

ATOM 1691 O SER B 139 13.533 33.168 43.776 1.00 39.51 O

ATOM 1692 CB SER B 139 14.486 36.205 43.421 1.00 49.71 C

ATOM 1693 OG SER B 139 13.361 36.761 42.763 1.00 58.32 O

ATOM 1694 H1 SER B 139 15.331 36.035 41.027 1.00 0.00 H

ATOM 1695 HA SER B 139 15.720 34.479 43.364 1.00 0.00 H

ATOM 1696 HB3 SER B 139 15.309 36.920 43.393 1.00 0.00 H

ATOM 1697 HB2 SER B 139 14.236 36.053 44.472 1.00 0.00 H

ATOM 1698 HG SER B 139 13.097 37.543 43.225 1.00 0.00 H

ATOM 1699 N VAL B 140 12.956 33.892 41.719 1.00 39.58 N

ATOM 1700 CA VAL B 140 11.777 33.040 41.613 1.00 37.35 C

ATOM 1701 C VAL B 140 12.160 31.559 41.469 1.00 37.74 C

ATOM 1702 O VAL B 140 11.338 30.665 41.694 1.00 36.48 O

ATOM 1703 CB VAL B 140 10.875 33.502 40.441 1.00 44.69 C

ATOM 1704 CG1 VAL B 140 11.556 33.242 39.103 1.00 42.42 C

ATOM 1705 CG2 VAL B 140 9.506 32.837 40.507 1.00 48.39 C

ATOM 1706 H VAL B 140 13.164 34.525 40.961 1.00 0.00 H

ATOM 1707 HA VAL B 140 11.207 33.144 42.539 1.00 0.00 H

ATOM 1708 HB VAL B 140 10.725 34.578 40.546 1.00 0.00 H

ATOM 1709 HG11 VAL B 140 11.063 33.812 38.315 1.00 0.00 H

ATOM 1710 HG12 VAL B 140 12.608 33.529 39.095 1.00 0.00 H

ATOM 1711 HG13 VAL B 140 11.476 32.197 38.816 1.00 0.00 H

ATOM 1712 HG21 VAL B 140 8.828 33.294 39.786 1.00 0.00 H

ATOM 1713 HG22 VAL B 140 9.540 31.773 40.273 1.00 0.00 H

ATOM 1714 HG23 VAL B 140 9.053 32.951 41.492 1.00 0.00 H

ATOM 1715 N GLU B 141 13.416 31.304 41.115 1.00 29.47 N

ATOM 1716 CA GLU B 141 13.914 29.938 41.003 1.00 21.28 C

ATOM 1717 C GLU B 141 15.071 29.682 41.954 1.00 21.80 C

ATOM 1718 O GLU B 141 15.977 28.921 41.647 1.00 20.94 O

ATOM 1719 CB GLU B 141 14.336 29.633 39.568 1.00 25.71 C

ATOM 1720 CG GLU B 141 13.181 29.705 38.590 1.00 22.95 C

ATOM 1721 CD GLU B 141 13.476 29.017 37.275 1.00 27.77 C

ATOM 1722 OE1 GLU B 141 12.592 29.049 36.392 1.00 26.50 O

ATOM 1723 OE2 GLU B 141 14.580 28.444 37.122 1.00 32.76 O1-

ATOM 1724 H GLU B 141 14.067 32.066 40.978 1.00 0.00 H

ATOM 1725 HA GLU B 141 13.165 29.199 41.283 1.00 0.00 H

ATOM 1726 HB3 GLU B 141 14.754 28.625 39.544 1.00 0.00 H

ATOM 1727 HB2 GLU B 141 15.133 30.308 39.251 1.00 0.00 H

ATOM 1728 HG3 GLU B 141 12.955 30.744 38.372 1.00 0.00 H

ATOM 1729 HG2 GLU B 141 12.285 29.251 39.015 1.00 0.00 H

ATOM 1730 N THR B 142 15.021 30.321 43.115 1.00 21.00 N

ATOM 1731 CA THR B 142 16.023 30.134 44.142 1.00 19.09 C

ATOM 1732 C THR B 142 15.495 29.218 45.233 1.00 14.46 C

ATOM 1733 O THR B 142 14.346 29.350 45.670 1.00 22.79 O

ATOM 1734 CB THR B 142 16.384 31.484 44.776 1.00 25.90 C

ATOM 1735 OG1 THR B 142 16.918 32.343 43.766 1.00 25.63 O

ATOM 1736 CG2 THR B 142 17.417 31.304 45.885 1.00 23.61 C

ATOM 1737 H THR B 142 14.253 30.949 43.307 1.00 0.00 H

ATOM 1738 HA THR B 142 16.937 29.713 43.725 1.00 0.00 H

ATOM 1739 HB THR B 142 15.497 31.967 45.189 1.00 0.00 H

ATOM 1740 HG1 THR B 142 17.660 31.914 43.356 1.00 0.00 H

ATOM 1741 HG21 THR B 142 17.777 32.274 46.229 1.00 0.00 H

ATOM 1742 HG22 THR B 142 17.015 30.800 46.763 1.00 0.00 H

ATOM 1743 HG23 THR B 142 18.285 30.741 45.538 1.00 0.00 H

ATOM 1744 N ILE B 143 16.314 28.264 45.662 1.00 13.80 N

ATOM 1745 CA AILE B 143 15.978 27.391 46.785 0.52 15.81 C

ATOM 1746 CA BILE B 143 15.940 27.453 46.801 0.48 15.82 C

ATOM 1747 C ILE B 143 16.977 27.619 47.898 1.00 13.98 C

ATOM 1748 O ILE B 143 18.180 27.557 47.660 1.00 14.03 O

ATOM 1749 CB AILE B 143 16.081 25.909 46.412 0.52 16.71 C

ATOM 1750 CB BILE B 143 15.714 25.976 46.422 0.48 16.79 C

ATOM 1751 CG1AILE B 143 15.044 25.544 45.361 0.52 15.25 C

ATOM 1752 CG1BILE B 143 15.374 25.147 47.659 0.48 17.21 C

ATOM 1753 CG2AILE B 143 15.889 25.036 47.639 0.52 17.89 C

ATOM 1754 CG2BILE B 143 16.916 25.407 45.696 0.48 15.62 C

ATOM 1755 CD1AILE B 143 15.225 24.142 44.805 0.52 17.42 C

ATOM 1756 CD1BILE B 143 15.363 23.675 47.386 0.48 20.26 C

ATOM 1757 H ILE B 143 17.237 28.178 45.250 1.00 0.00 H

ATOM 1758 HA AILE B 143 14.975 27.579 47.174 0.52 0.00 H

ATOM 1759 HA BILE B 143 14.995 27.841 47.183 0.48 0.00 H

ATOM 1760 HB AILE B 143 17.072 25.710 45.996 0.52 0.00 H

ATOM 1761 HB BILE B 143 14.861 25.931 45.744 0.48 0.00 H

ATOM 1762 HG13AILE B 143 15.187 26.258 44.564 0.52 0.00 H

ATOM 1763 HG13BILE B 143 14.400 25.451 48.043 0.48 0.00 H

ATOM 1764 HG12AILE B 143 14.037 25.685 45.752 0.52 0.00 H

ATOM 1765 HG12BILE B 143 16.093 25.363 48.449 0.48 0.00 H

ATOM 1766 HG21AILE B 143 15.875 23.977 47.391 0.52 0.00 H

ATOM 1767 HG21BILE B 143 16.727 24.364 45.442 0.48 0.00 H

ATOM 1768 HG22AILE B 143 16.692 25.120 48.369 0.52 0.00 H

ATOM 1769 HG22BILE B 143 17.093 25.977 44.784 0.48 0.00 H

ATOM 1770 HG23AILE B 143 14.950 25.258 48.147 0.52 0.00 H

ATOM 1771 HG23BILE B 143 17.793 25.470 46.340 0.48 0.00 H

ATOM 1772 HD11AILE B 143 14.596 23.986 43.929 0.52 0.00 H

ATOM 1773 HD11BILE B 143 15.115 23.137 48.301 0.48 0.00 H

ATOM 1774 HD12AILE B 143 16.254 23.986 44.505 0.52 0.00 H

ATOM 1775 HD12BILE B 143 14.619 23.452 46.622 0.48 0.00 H

ATOM 1776 HD13AILE B 143 14.947 23.372 45.523 0.52 0.00 H

ATOM 1777 HD13BILE B 143 16.347 23.362 47.036 0.48 0.00 H

ATOM 1778 N GLU B 144 16.482 27.871 49.103 1.00 13.87 N

ATOM 1779 CA GLU B 144 17.343 28.045 50.249 1.00 14.49 C

ATOM 1780 C GLU B 144 17.534 26.706 50.936 1.00 14.93 C

ATOM 1781 O GLU B 144 16.585 26.142 51.489 1.00 19.03 O

ATOM 1782 CB GLU B 144 16.725 29.059 51.214 1.00 14.90 C

ATOM 1783 CG GLU B 144 17.562 29.286 52.448 1.00 14.27 C

ATOM 1784 CD GLU B 144 16.906 30.210 53.464 1.00 16.60 C

ATOM 1785 OE1 GLU B 144 15.791 30.710 53.204 1.00 20.49 O

ATOM 1786 OE2 GLU B 144 17.542 30.425 54.509 1.00 17.21 O1-

ATOM 1787 H GLU B 144 15.483 27.903 49.242 1.00 0.00 H

ATOM 1788 HA GLU B 144 18.317 28.444 49.957 1.00 0.00 H

ATOM 1789 HB3 GLU B 144 15.727 28.720 51.502 1.00 0.00 H

ATOM 1790 HB2 GLU B 144 16.578 30.005 50.691 1.00 0.00 H

ATOM 1791 HG3 GLU B 144 18.499 29.743 52.137 1.00 0.00 H

ATOM 1792 HG2 GLU B 144 17.800 28.360 52.972 1.00 0.00 H

ATOM 1793 N LEU B 145 18.749 26.176 50.881 1.00 12.44 N

ATOM 1794 CA LEU B 145 19.046 24.919 51.554 1.00 12.98 C

ATOM 1795 C LEU B 145 19.439 25.231 52.987 1.00 13.63 C

ATOM 1796 O LEU B 145 20.506 25.798 53.222 1.00 17.72 O

ATOM 1797 CB LEU B 145 20.185 24.169 50.851 1.00 12.08 C

ATOM 1798 CG LEU B 145 19.974 23.932 49.349 1.00 14.70 C

ATOM 1799 CD1 LEU B 145 21.216 23.284 48.768 1.00 14.34 C

ATOM 1800 CD2 LEU B 145 18.751 23.070 49.077 1.00 13.24 C

ATOM 1801 H LEU B 145 19.499 26.656 50.399 1.00 0.00 H

ATOM 1802 HA LEU B 145 18.176 24.259 51.565 1.00 0.00 H

ATOM 1803 HB3 LEU B 145 20.343 23.213 51.344 1.00 0.00 H

ATOM 1804 HB2 LEU B 145 21.117 24.719 50.977 1.00 0.00 H

ATOM 1805 HG LEU B 145 19.836 24.889 48.844 1.00 0.00 H

ATOM 1806 HD11 LEU B 145 21.107 23.116 47.696 1.00 0.00 H

ATOM 1807 HD12 LEU B 145 22.094 23.916 48.907 1.00 0.00 H

ATOM 1808 HD13 LEU B 145 21.422 22.320 49.235 1.00 0.00 H

ATOM 1809 HD21 LEU B 145 18.592 22.951 48.005 1.00 0.00 H

ATOM 1810 HD22 LEU B 145 18.873 22.075 49.499 1.00 0.00 H

ATOM 1811 HD23 LEU B 145 17.839 23.496 49.489 1.00 0.00 H

ATOM 1812 N LYS B 146 18.578 24.857 53.931 1.00 14.37 N

ATOM 1813 CA LYS B 146 18.860 25.090 55.352 1.00 13.62 C

ATOM 1814 C LYS B 146 19.938 24.130 55.874 1.00 17.02 C

ATOM 1815 O LYS B 146 19.869 22.912 55.653 1.00 15.40 O

ATOM 1816 CB LYS B 146 17.580 24.952 56.179 1.00 13.79 C

ATOM 1817 CG LYS B 146 16.491 25.970 55.858 1.00 15.34 C

ATOM 1818 CD LYS B 146 16.815 27.367 56.382 1.00 15.73 C

ATOM 1819 CE LYS B 146 15.626 28.304 56.176 1.00 13.64 C

ATOM 1820 NZ LYS B 146 15.896 29.711 56.608 1.00 15.71 N1+

ATOM 1821 H LYS B 146 17.713 24.402 53.675 1.00 0.00 H

ATOM 1822 HA LYS B 146 19.231 26.110 55.470 1.00 0.00 H

ATOM 1823 HB3 LYS B 146 17.814 25.007 57.244 1.00 0.00 H

ATOM 1824 HB2 LYS B 146 17.170 23.952 56.023 1.00 0.00 H

ATOM 1825 HG3 LYS B 146 15.564 25.629 56.322 1.00 0.00 H

ATOM 1826 HG2 LYS B 146 16.294 26.002 54.786 1.00 0.00 H

ATOM 1827 HD3 LYS B 146 17.684 27.763 55.860 1.00 0.00 H

ATOM 1828 HD2 LYS B 146 17.072 27.325 57.441 1.00 0.00 H

ATOM 1829 HE3 LYS B 146 14.765 27.938 56.737 1.00 0.00 H

ATOM 1830 HE2 LYS B 146 15.332 28.318 55.126 1.00 0.00 H

ATOM 1831 HZ1 LYS B 146 16.607 30.101 55.994 1.00 0.00 H

ATOM 1832 HZ2 LYS B 146 15.054 30.261 56.515 1.00 0.00 H

ATOM 1833 HZ3 LYS B 146 16.219 29.730 57.564 1.00 0.00 H

ATOM 1834 N ARG B 147 20.927 24.679 56.579 1.00 15.84 N

ATOM 1835 CA ARG B 147 22.098 23.906 56.998 1.00 18.59 C

ATOM 1836 C ARG B 147 22.750 24.403 58.287 1.00 16.91 C

ATOM 1837 O ARG B 147 23.931 24.724 58.293 1.00 21.79 O

ATOM 1838 CB ARG B 147 23.176 23.978 55.909 1.00 26.89 C

ATOM 1839 CG ARG B 147 22.998 23.051 54.745 1.00 28.20 C

ATOM 1840 CD ARG B 147 24.013 23.358 53.662 1.00 21.10 C

ATOM 1841 NE ARG B 147 25.341 23.610 54.203 1.00 28.58 N

ATOM 1842 CZ ARG B 147 26.293 22.691 54.305 1.00 25.79 C

ATOM 1843 NH1 ARG B 147 26.063 21.449 53.910 1.00 32.57 N

ATOM 1844 NH2 ARG B 147 27.472 23.016 54.805 1.00 28.35 N1+

ATOM 1845 H ARG B 147 20.934 25.676 56.741 1.00 0.00 H

ATOM 1846 HA ARG B 147 21.840 22.862 57.186 1.00 0.00 H

ATOM 1847 HB3 ARG B 147 24.142 23.672 56.307 1.00 0.00 H

ATOM 1848 HB2 ARG B 147 23.277 24.988 55.529 1.00 0.00 H

ATOM 1849 HG3 ARG B 147 22.010 23.158 54.306 1.00 0.00 H

ATOM 1850 HG2 ARG B 147 23.092 22.026 55.093 1.00 0.00 H

ATOM 1851 HD3 ARG B 147 23.686 24.261 53.153 1.00 0.00 H

ATOM 1852 HD2 ARG B 147 24.014 22.591 52.896 1.00 0.00 H

ATOM 1853 HE ARG B 147 25.521 24.547 54.535 1.00 0.00 H

ATOM 1854 HH12 ARG B 147 26.789 20.750 53.975 1.00 0.00 H

ATOM 1855 HH11 ARG B 147 25.137 21.170 53.601 1.00 0.00 H

ATOM 1856 HH22 ARG B 147 27.654 23.958 55.119 1.00 0.00 H

ATOM 1857 HH21 ARG B 147 28.210 22.330 54.877 1.00 0.00 H

ATOM 1858 N GLY B 148 22.018 24.461 59.384 1.00 16.62 N

ATOM 1859 CA GLY B 148 22.651 24.889 60.616 1.00 17.38 C

ATOM 1860 C GLY B 148 22.907 26.384 60.658 1.00 17.15 C

ATOM 1861 O GLY B 148 21.966 27.163 60.545 1.00 17.71 O

ATOM 1862 H GLY B 148 21.041 24.208 59.379 1.00 0.00 H

ATOM 1863 HA3 GLY B 148 23.567 24.326 60.809 1.00 0.00 H

ATOM 1864 HA2 GLY B 148 21.976 24.644 61.436 1.00 0.00 H

ATOM 1865 N SER B 149 24.174 26.780 60.791 1.00 18.70 N

ATOM 1866 CA ASER B 149 24.506 28.197 60.956 0.59 17.93 C

ATOM 1867 CA BSER B 149 24.571 28.188 60.936 0.41 17.99 C

ATOM 1868 C SER B 149 24.311 29.053 59.703 1.00 19.29 C

ATOM 1869 O SER B 149 24.052 30.244 59.816 1.00 18.38 O

ATOM 1870 CB ASER B 149 25.936 28.369 61.473 0.59 22.33 C

ATOM 1871 CB BSER B 149 26.064 28.293 61.281 0.41 21.93 C

ATOM 1872 OG ASER B 149 26.862 27.764 60.597 0.59 19.78 O

ATOM 1873 OG BSER B 149 26.359 27.743 62.553 0.41 22.34 O

ATOM 1874 H SER B 149 24.922 26.102 60.837 1.00 0.00 H

ATOM 1875 HA ASER B 149 23.845 28.608 61.722 0.59 0.00 H

ATOM 1876 HA BSER B 149 24.006 28.611 61.766 0.41 0.00 H

ATOM 1877 HB3ASER B 149 26.036 27.904 62.455 0.59 0.00 H

ATOM 1878 HB3BSER B 149 26.368 29.339 61.259 0.41 0.00 H

ATOM 1879 HB2ASER B 149 26.193 29.422 61.597 0.59 0.00 H

ATOM 1880 HB2BSER B 149 26.649 27.778 60.519 0.41 0.00 H

ATOM 1881 HG ASER B 149 26.900 28.269 59.796 0.59 0.00 H

ATOM 1882 HG BSER B 149 27.299 27.829 62.729 0.41 0.00 H

ATOM 1883 N ASN B 150 24.438 28.456 58.519 1.00 16.02 N

ATOM 1884 CA ASN B 150 24.313 29.186 57.255 1.00 18.64 C

ATOM 1885 C ASN B 150 23.414 28.430 56.292 1.00 17.08 C

ATOM 1886 O ASN B 150 23.445 27.211 56.248 1.00 26.33 O

ATOM 1887 CB ASN B 150 25.667 29.328 56.530 1.00 19.52 C

ATOM 1888 CG ASN B 150 26.692 30.101 57.318 1.00 23.78 C

ATOM 1889 OD1 ASN B 150 27.317 29.564 58.236 1.00 27.41 O

ATOM 1890 ND2 ASN B 150 26.896 31.363 56.949 1.00 19.52 N

ATOM 1891 H ASN B 150 24.620 27.463 58.479 1.00 0.00 H

ATOM 1892 HA ASN B 150 23.883 30.180 57.399 1.00 0.00 H

ATOM 1893 HB3 ASN B 150 25.531 29.809 55.559 1.00 0.00 H

ATOM 1894 HB2 ASN B 150 26.089 28.344 56.317 1.00 0.00 H

ATOM 1895 HD22 ASN B 150 27.580 31.920 57.439 1.00 0.00 H

ATOM 1896 HD21 ASN B 150 26.381 31.754 56.174 1.00 0.00 H

ATOM 1897 N SER B 151 22.631 29.161 55.510 1.00 14.01 N

ATOM 1898 CA SER B 151 21.917 28.568 54.382 1.00 15.60 C

ATOM 1899 C SER B 151 22.829 28.616 53.166 1.00 12.76 C

ATOM 1900 O SER B 151 23.780 29.400 53.115 1.00 14.71 O

ATOM 1901 CB SER B 151 20.650 29.365 54.066 1.00 17.02 C

ATOM 1902 OG SER B 151 19.693 29.267 55.106 1.00 18.46 O

ATOM 1903 H SER B 151 22.631 30.166 55.596 1.00 0.00 H

ATOM 1904 HA SER B 151 21.640 27.536 54.598 1.00 0.00 H

ATOM 1905 HB3 SER B 151 20.203 28.962 53.160 1.00 0.00 H

ATOM 1906 HB2 SER B 151 20.872 30.417 53.881 1.00 0.00 H

ATOM 1907 HG SER B 151 18.911 29.755 54.850 1.00 0.00 H

ATOM 1908 N VAL B 152 22.535 27.752 52.200 1.00 13.80 N

ATOM 1909 CA VAL B 152 23.164 27.800 50.888 1.00 14.63 C

ATOM 1910 C VAL B 152 22.034 28.010 49.892 1.00 13.49 C

ATOM 1911 O VAL B 152 21.169 27.156 49.769 1.00 14.85 O

ATOM 1912 CB VAL B 152 23.873 26.475 50.579 1.00 16.65 C

ATOM 1913 CG1 VAL B 152 24.476 26.505 49.188 1.00 18.09 C

ATOM 1914 CG2 VAL B 152 24.958 26.198 51.639 1.00 20.55 C

ATOM 1915 H VAL B 152 21.765 27.108 52.332 1.00 0.00 H

ATOM 1916 HA VAL B 152 23.880 28.620 50.794 1.00 0.00 H

ATOM 1917 HB VAL B 152 23.158 25.653 50.623 1.00 0.00 H

ATOM 1918 HG11 VAL B 152 24.920 25.544 48.945 1.00 0.00 H

ATOM 1919 HG12 VAL B 152 23.766 26.725 48.396 1.00 0.00 H

ATOM 1920 HG13 VAL B 152 25.272 27.245 49.142 1.00 0.00 H

ATOM 1921 HG21 VAL B 152 25.425 25.226 51.474 1.00 0.00 H

ATOM 1922 HG22 VAL B 152 25.747 26.949 51.592 1.00 0.00 H

ATOM 1923 HG23 VAL B 152 24.573 26.208 52.657 1.00 0.00 H

ATOM 1924 N TYR B 153 22.030 29.158 49.207 1.00 12.40 N

ATOM 1925 CA TYR B 153 20.981 29.474 48.239 1.00 10.32 C

ATOM 1926 C TYR B 153 21.461 29.072 46.856 1.00 13.40 C

ATOM 1927 O TYR B 153 22.496 29.555 46.393 1.00 15.15 O

ATOM 1928 CB TYR B 153 20.688 30.978 48.224 1.00 12.02 C

ATOM 1929 CG TYR B 153 20.079 31.559 49.493 1.00 13.90 C

ATOM 1930 CD1 TYR B 153 18.718 31.833 49.561 1.00 17.31 C

ATOM 1931 CD2 TYR B 153 20.873 31.889 50.583 1.00 14.15 C

ATOM 1932 CE1 TYR B 153 18.160 32.393 50.693 1.00 17.36 C

ATOM 1933 CE2 TYR B 153 20.315 32.446 51.730 1.00 14.77 C

ATOM 1934 CZ TYR B 153 18.966 32.698 51.762 1.00 17.24 C

ATOM 1935 OH TYR B 153 18.399 33.257 52.885 1.00 19.23 O

ATOM 1936 H TYR B 153 22.769 29.831 49.348 1.00 0.00 H

ATOM 1937 HA TYR B 153 20.053 28.962 48.483 1.00 0.00 H

ATOM 1938 HB3 TYR B 153 20.014 31.209 47.397 1.00 0.00 H

ATOM 1939 HB2 TYR B 153 21.602 31.537 48.014 1.00 0.00 H

ATOM 1940 HD1 TYR B 153 18.078 31.598 48.724 1.00 0.00 H

ATOM 1941 HD2 TYR B 153 21.938 31.707 50.551 1.00 0.00 H

ATOM 1942 HE1 TYR B 153 17.098 32.588 50.731 1.00 0.00 H

ATOM 1943 HE2 TYR B 153 20.939 32.687 52.578 1.00 0.00 H

ATOM 1944 HH TYR B 153 17.453 33.246 52.866 1.00 0.00 H

ATOM 1945 N VAL B 154 20.707 28.192 46.201 1.00 12.50 N

ATOM 1946 CA VAL B 154 21.063 27.748 44.852 1.00 13.21 C

ATOM 1947 C VAL B 154 19.887 27.951 43.898 1.00 14.85 C

ATOM 1948 O VAL B 154 18.748 28.114 44.331 1.00 17.19 O

ATOM 1949 CB VAL B 154 21.481 26.272 44.852 1.00 19.35 C

ATOM 1950 CG1 VAL B 154 22.630 26.047 45.830 1.00 20.79 C

ATOM 1951 CG2 VAL B 154 20.312 25.407 45.240 1.00 17.23 C

ATOM 1952 H VAL B 154 19.855 27.840 46.618 1.00 0.00 H

ATOM 1953 HA VAL B 154 21.889 28.327 44.434 1.00 0.00 H

ATOM 1954 HB VAL B 154 21.820 25.988 43.854 1.00 0.00 H

ATOM 1955 HG11 VAL B 154 23.037 25.041 45.722 1.00 0.00 H

ATOM 1956 HG12 VAL B 154 23.447 26.749 45.660 1.00 0.00 H

ATOM 1957 HG13 VAL B 154 22.298 26.147 46.863 1.00 0.00 H

ATOM 1958 HG21 VAL B 154 20.632 24.370 45.343 1.00 0.00 H

ATOM 1959 HG22 VAL B 154 19.869 25.693 46.195 1.00 0.00 H

ATOM 1960 HG23 VAL B 154 19.521 25.393 44.490 1.00 0.00 H

ATOM 1961 N GLN B 155 20.169 27.956 42.600 1.00 13.75 N

ATOM 1962 CA GLN B 155 19.117 28.029 41.600 1.00 14.74 C

ATOM 1963 C GLN B 155 18.576 26.643 41.269 1.00 14.71 C

ATOM 1964 O GLN B 155 19.308 25.663 41.346 1.00 15.71 O

ATOM 1965 CB GLN B 155 19.634 28.713 40.332 1.00 17.96 C

ATOM 1966 CG GLN B 155 20.013 30.168 40.569 1.00 18.22 C

ATOM 1967 CD GLN B 155 18.869 30.960 41.152 1.00 25.04 C

ATOM 1968 OE1 GLN B 155 18.854 31.268 42.351 1.00 27.43 O

ATOM 1969 NE2 GLN B 155 17.890 31.285 40.315 1.00 24.25 N

ATOM 1970 H GLN B 155 21.119 27.798 42.275 1.00 0.00 H

ATOM 1971 HA GLN B 155 18.287 28.594 42.005 1.00 0.00 H

ATOM 1972 HB3 GLN B 155 18.863 28.676 39.560 1.00 0.00 H

ATOM 1973 HB2 GLN B 155 20.481 28.176 39.910 1.00 0.00 H

ATOM 1974 HG3 GLN B 155 20.329 30.627 39.632 1.00 0.00 H

ATOM 1975 HG2 GLN B 155 20.870 30.231 41.241 1.00 0.00 H

ATOM 1976 HE22 GLN B 155 17.096 31.814 40.660 1.00 0.00 H

ATOM 1977 HE21 GLN B 155 17.932 31.000 39.348 1.00 0.00 H

ATOM 1978 N TYR B 156 17.297 26.573 40.899 1.00 15.64 N

ATOM 1979 CA TYR B 156 16.666 25.286 40.563 1.00 16.40 C

ATOM 1980 C TYR B 156 17.534 24.438 39.641 1.00 16.98 C

ATOM 1981 O TYR B 156 17.745 23.252 39.887 1.00 16.87 O

ATOM 1982 CB TYR B 156 15.322 25.490 39.858 1.00 19.05 C

ATOM 1983 CG TYR B 156 14.155 25.955 40.702 1.00 17.99 C

ATOM 1984 CD1 TYR B 156 14.265 26.114 42.072 1.00 21.63 C

ATOM 1985 CD2 TYR B 156 12.933 26.240 40.105 1.00 21.73 C

ATOM 1986 CE1 TYR B 156 13.176 26.544 42.829 1.00 24.44 C

ATOM 1987 CE2 TYR B 156 11.854 26.668 40.850 1.00 24.86 C

ATOM 1988 CZ TYR B 156 11.982 26.817 42.204 1.00 25.85 C

ATOM 1989 OH TYR B 156 10.903 27.247 42.936 1.00 28.27 O

ATOM 1990 H TYR B 156 16.729 27.412 40.895 1.00 0.00 H

ATOM 1991 HA TYR B 156 16.539 24.700 41.473 1.00 0.00 H

ATOM 1992 HB3 TYR B 156 15.000 24.550 39.411 1.00 0.00 H

ATOM 1993 HB2 TYR B 156 15.438 26.190 39.028 1.00 0.00 H

ATOM 1994 HD1 TYR B 156 15.204 25.931 42.559 1.00 0.00 H

ATOM 1995 HD2 TYR B 156 12.823 26.131 39.038 1.00 0.00 H

ATOM 1996 HE1 TYR B 156 13.238 26.702 43.891 1.00 0.00 H

ATOM 1997 HE2 TYR B 156 10.917 26.887 40.358 1.00 0.00 H

ATOM 1998 HH TYR B 156 10.138 27.414 42.407 1.00 0.00 H

ATOM 1999 N ASP B 157 18.037 25.048 38.576 1.00 16.30 N

ATOM 2000 CA ASP B 157 18.714 24.259 37.559 1.00 19.30 C

ATOM 2001 C ASP B 157 20.190 24.020 37.856 1.00 15.40 C

ATOM 2002 O ASP B 157 20.890 23.373 37.084 1.00 16.69 O

ATOM 2003 CB ASP B 157 18.468 24.821 36.160 1.00 16.98 C

ATOM 2004 CG ASP B 157 17.048 24.553 35.664 1.00 19.30 C

ATOM 2005 OD1 ASP B 157 16.259 23.897 36.378 1.00 17.37 O

ATOM 2006 OD2 ASP B 157 16.719 24.986 34.536 1.00 18.76 O1-

ATOM 2007 H ASP B 157 17.851 26.026 38.406 1.00 0.00 H

ATOM 2008 HA ASP B 157 18.332 23.238 37.542 1.00 0.00 H

ATOM 2009 HB3 ASP B 157 19.143 24.369 35.432 1.00 0.00 H

ATOM 2010 HB2 ASP B 157 18.660 25.895 36.138 1.00 0.00 H

ATOM 2011 N ASP B 158 20.652 24.509 39.005 1.00 16.26 N

ATOM 2012 CA ASP B 158 21.922 24.060 39.563 1.00 16.66 C

ATOM 2013 C ASP B 158 21.791 22.628 40.103 1.00 14.43 C

ATOM 2014 O ASP B 158 22.793 21.948 40.345 1.00 17.07 O

ATOM 2015 CB ASP B 158 22.336 24.942 40.751 1.00 18.44 C

ATOM 2016 CG ASP B 158 22.658 26.364 40.366 1.00 26.73 C

ATOM 2017 OD1 ASP B 158 23.141 26.596 39.248 1.00 24.54 O

ATOM 2018 OD2 ASP B 158 22.451 27.257 41.218 1.00 26.81 O1-

ATOM 2019 H ASP B 158 20.038 25.049 39.600 1.00 0.00 H

ATOM 2020 HA ASP B 158 22.703 24.068 38.800 1.00 0.00 H

ATOM 2021 HB3 ASP B 158 23.250 24.545 41.196 1.00 0.00 H

ATOM 2022 HB2 ASP B 158 21.601 24.921 41.553 1.00 0.00 H

ATOM 2023 N ILE B 159 20.558 22.180 40.328 1.00 13.75 N

ATOM 2024 CA ILE B 159 20.338 20.893 40.972 1.00 14.07 C

ATOM 2025 C ILE B 159 20.072 19.807 39.939 1.00 12.61 C

ATOM 2026 O ILE B 159 19.163 19.937 39.115 1.00 14.80 O

ATOM 2027 CB ILE B 159 19.147 20.965 41.940 1.00 14.22 C

ATOM 2028 CG1 ILE B 159 19.434 22.008 43.024 1.00 17.93 C

ATOM 2029 CG2 ILE B 159 18.886 19.617 42.588 1.00 15.56 C

ATOM 2030 CD1 ILE B 159 18.213 22.569 43.636 1.00 25.44 C

ATOM 2031 H ILE B 159 19.758 22.763 40.120 1.00 0.00 H

ATOM 2032 HA ILE B 159 21.205 20.598 41.567 1.00 0.00 H

ATOM 2033 HB ILE B 159 18.254 21.261 41.389 1.00 0.00 H

ATOM 2034 HG13 ILE B 159 20.024 22.851 42.665 1.00 0.00 H

ATOM 2035 HG12 ILE B 159 20.049 21.552 43.801 1.00 0.00 H

ATOM 2036 HG21 ILE B 159 18.177 19.698 43.409 1.00 0.00 H

ATOM 2037 HG22 ILE B 159 18.450 18.901 41.892 1.00 0.00 H

ATOM 2038 HG23 ILE B 159 19.798 19.179 42.997 1.00 0.00 H

ATOM 2039 HD11 ILE B 159 18.374 22.846 44.678 1.00 0.00 H

ATOM 2040 HD12 ILE B 159 17.917 23.466 43.091 1.00 0.00 H

ATOM 2041 HD13 ILE B 159 17.376 21.881 43.567 1.00 0.00 H

ATOM 2042 N MET B 160 20.861 18.737 39.987 1.00 12.31 N

ATOM 2043 CA MET B 160 20.591 17.557 39.165 1.00 13.64 C

ATOM 2044 C MET B 160 19.443 16.778 39.785 1.00 12.32 C

ATOM 2045 O MET B 160 18.436 16.499 39.133 1.00 11.86 O

ATOM 2046 CB MET B 160 21.826 16.649 39.075 1.00 12.05 C

ATOM 2047 CG MET B 160 23.119 17.358 38.576 1.00 12.06 C

ATOM 2048 SD MET B 160 23.013 17.890 36.859 1.00 15.73 S

ATOM 2049 CE MET B 160 23.137 19.659 37.072 1.00 18.33 C

ATOM 2050 H MET B 160 21.596 18.680 40.683 1.00 0.00 H

ATOM 2051 HA MET B 160 20.311 17.859 38.154 1.00 0.00 H

ATOM 2052 HB3 MET B 160 21.615 15.801 38.423 1.00 0.00 H

ATOM 2053 HB2 MET B 160 22.055 16.219 40.051 1.00 0.00 H

ATOM 2054 HG3 MET B 160 23.920 16.635 38.661 1.00 0.00 H

ATOM 2055 HG2 MET B 160 23.396 18.192 39.220 1.00 0.00 H

ATOM 2056 HE1 MET B 160 23.069 20.159 36.106 1.00 0.00 H

ATOM 2057 HE2 MET B 160 22.333 20.027 37.709 1.00 0.00 H

ATOM 2058 HE3 MET B 160 24.091 19.923 37.529 1.00 0.00 H

ATOM 2059 N PHE B 161 19.611 16.393 41.049 1.00 12.25 N

ATOM 2060 CA PHE B 161 18.552 15.704 41.777 1.00 11.43 C

ATOM 2061 C PHE B 161 18.787 15.844 43.277 1.00 13.16 C

ATOM 2062 O PHE B 161 19.864 16.239 43.692 1.00 11.88 O

ATOM 2063 CB PHE B 161 18.432 14.217 41.350 1.00 13.10 C

ATOM 2064 CG PHE B 161 19.672 13.380 41.603 1.00 12.06 C

ATOM 2065 CD1 PHE B 161 20.717 13.371 40.690 1.00 12.86 C

ATOM 2066 CD2 PHE B 161 19.778 12.591 42.740 1.00 16.19 C

ATOM 2067 CE1 PHE B 161 21.860 12.594 40.912 1.00 15.11 C

ATOM 2068 CE2 PHE B 161 20.907 11.807 42.963 1.00 14.04 C

ATOM 2069 CZ PHE B 161 21.950 11.815 42.051 1.00 13.35 C

ATOM 2070 H PHE B 161 20.456 16.636 41.548 1.00 0.00 H

ATOM 2071 HA PHE B 161 17.606 16.208 41.567 1.00 0.00 H

ATOM 2072 HB3 PHE B 161 18.191 14.150 40.291 1.00 0.00 H

ATOM 2073 HB2 PHE B 161 17.595 13.743 41.863 1.00 0.00 H

ATOM 2074 HD1 PHE B 161 20.655 13.970 39.794 1.00 0.00 H

ATOM 2075 HD2 PHE B 161 18.975 12.577 43.461 1.00 0.00 H

ATOM 2076 HE1 PHE B 161 22.665 12.603 40.193 1.00 0.00 H

ATOM 2077 HE2 PHE B 161 20.975 11.194 43.850 1.00 0.00 H

ATOM 2078 HZ PHE B 161 22.828 11.209 42.223 1.00 0.00 H

ATOM 2079 N PHE B 162 17.760 15.548 44.060 1.00 11.00 N

ATOM 2080 CA PHE B 162 17.862 15.443 45.511 1.00 13.02 C

ATOM 2081 C PHE B 162 17.814 13.972 45.904 1.00 12.90 C

ATOM 2082 O PHE B 162 17.096 13.188 45.278 1.00 14.73 O

ATOM 2083 CB PHE B 162 16.698 16.165 46.203 1.00 12.25 C

ATOM 2084 CG PHE B 162 16.609 17.637 45.919 1.00 12.61 C

ATOM 2085 CD1 PHE B 162 17.401 18.548 46.608 1.00 14.70 C

ATOM 2086 CD2 PHE B 162 15.683 18.119 45.012 1.00 14.71 C

ATOM 2087 CE1 PHE B 162 17.294 19.922 46.373 1.00 13.50 C

ATOM 2088 CE2 PHE B 162 15.572 19.489 44.772 1.00 15.39 C

ATOM 2089 CZ PHE B 162 16.373 20.388 45.468 1.00 15.10 C

ATOM 2090 H PHE B 162 16.886 15.268 43.630 1.00 0.00 H

ATOM 2091 HA PHE B 162 18.788 15.890 45.863 1.00 0.00 H

ATOM 2092 HB3 PHE B 162 16.784 16.046 47.285 1.00 0.00 H

ATOM 2093 HB2 PHE B 162 15.753 15.694 45.937 1.00 0.00 H

ATOM 2094 HD1 PHE B 162 18.121 18.188 47.327 1.00 0.00 H

ATOM 2095 HD2 PHE B 162 15.043 17.435 44.474 1.00 0.00 H

ATOM 2096 HE1 PHE B 162 17.916 20.615 46.920 1.00 0.00 H

ATOM 2097 HE2 PHE B 162 14.841 19.855 44.067 1.00 0.00 H

ATOM 2098 HZ PHE B 162 16.249 21.449 45.326 1.00 0.00 H

ATOM 2099 N GLU B 163 18.543 13.604 46.961 1.00 14.21 N

ATOM 2100 CA GLU B 163 18.589 12.220 47.437 1.00 12.37 C

ATOM 2101 C GLU B 163 18.400 12.220 48.943 1.00 15.71 C

ATOM 2102 O GLU B 163 18.922 13.082 49.628 1.00 17.26 O

ATOM 2103 CB GLU B 163 19.950 11.614 47.099 1.00 15.41 C

ATOM 2104 CG GLU B 163 20.180 10.216 47.618 1.00 16.40 C

ATOM 2105 CD GLU B 163 21.566 9.697 47.282 1.00 20.56 C

ATOM 2106 OE1 GLU B 163 22.462 10.508 46.946 1.00 20.97 O

ATOM 2107 OE2 GLU B 163 21.765 8.468 47.363 1.00 29.03 O1-

ATOM 2108 H GLU B 163 19.118 14.290 47.434 1.00 0.00 H

ATOM 2109 HA GLU B 163 17.838 11.635 46.945 1.00 0.00 H

ATOM 2110 HB3 GLU B 163 20.728 12.278 47.475 1.00 0.00 H

ATOM 2111 HB2 GLU B 163 20.063 11.612 46.014 1.00 0.00 H

ATOM 2112 HG3 GLU B 163 19.416 9.527 47.258 1.00 0.00 H

ATOM 2113 HG2 GLU B 163 20.144 10.204 48.700 1.00 0.00 H

ATOM 2114 N SER B 164 17.636 11.271 49.461 1.00 17.15 N

ATOM 2115 CA SER B 164 17.568 11.128 50.903 1.00 19.46 C

ATOM 2116 C SER B 164 18.796 10.351 51.340 1.00 27.51 C

ATOM 2117 O SER B 164 19.144 9.337 50.740 1.00 31.11 O

ATOM 2118 CB SER B 164 16.285 10.416 51.325 1.00 26.22 C

ATOM 2119 OG SER B 164 16.144 9.186 50.652 1.00 25.12 O

ATOM 2120 H SER B 164 17.194 10.578 48.871 1.00 0.00 H

ATOM 2121 HA SER B 164 17.585 12.092 51.403 1.00 0.00 H

ATOM 2122 HB3 SER B 164 15.418 11.039 51.107 1.00 0.00 H

ATOM 2123 HB2 SER B 164 16.279 10.237 52.401 1.00 0.00 H

ATOM 2124 HG SER B 164 16.907 8.658 50.840 1.00 0.00 H

ATOM 2125 N SER B 165 19.469 10.839 52.374 1.00 31.79 N

ATOM 2126 CA SER B 165 20.627 10.128 52.894 1.00 39.59 C

ATOM 2127 C SER B 165 20.165 8.907 53.675 1.00 44.58 C

ATOM 2128 O SER B 165 19.226 8.987 54.464 1.00 36.80 O

ATOM 2129 CB SER B 165 21.468 11.031 53.792 1.00 42.68 C

ATOM 2130 OG SER B 165 22.577 10.315 54.305 1.00 42.93 O

ATOM 2131 H SER B 165 19.160 11.677 52.848 1.00 0.00 H

ATOM 2132 HA SER B 165 21.261 9.813 52.061 1.00 0.00 H

ATOM 2133 HB3 SER B 165 20.888 11.397 54.636 1.00 0.00 H

ATOM 2134 HB2 SER B 165 21.825 11.905 53.247 1.00 0.00 H

ATOM 2135 HG SER B 165 23.186 10.163 53.597 1.00 0.00 H

ATOM 2136 N THR B 166 20.814 7.772 53.441 1.00 49.15 N

ATOM 2137 CA THR B 166 20.541 6.578 54.229 1.00 49.16 C

ATOM 2138 C THR B 166 21.408 6.628 55.484 1.00 48.99 C

ATOM 2139 O THR B 166 21.208 5.864 56.429 1.00 54.58 O

ATOM 2140 CB THR B 166 20.831 5.288 53.433 1.00 57.47 C

ATOM 2141 OG1 THR B 166 22.233 4.993 53.475 1.00 64.79 O

ATOM 2142 CG2 THR B 166 20.385 5.445 51.981 1.00 53.74 C

ATOM 2143 H THR B 166 21.547 7.735 52.747 1.00 0.00 H

ATOM 2144 HA THR B 166 19.491 6.544 54.529 1.00 0.00 H

ATOM 2145 HB THR B 166 20.300 4.452 53.890 1.00 0.00 H

ATOM 2146 HG1 THR B 166 22.470 4.795 54.369 1.00 0.00 H

ATOM 2147 HG21 THR B 166 20.469 4.494 51.454 1.00 0.00 H

ATOM 2148 HG22 THR B 166 19.342 5.759 51.918 1.00 0.00 H

ATOM 2149 HG23 THR B 166 20.990 6.164 51.427 1.00 0.00 H

ATOM 2150 N LYS B 167 22.361 7.556 55.479 1.00 47.02 N

ATOM 2151 CA LYS B 167 23.311 7.728 56.570 1.00 44.40 C

ATOM 2152 C LYS B 167 22.782 8.657 57.659 1.00 45.45 C

ATOM 2153 O LYS B 167 23.187 8.559 58.819 1.00 46.61 O

ATOM 2154 CB LYS B 167 24.635 8.272 56.032 1.00 44.37 C

ATOM 2155 CG LYS B 167 25.398 7.299 55.152 1.00 48.38 C

ATOM 2156 CD LYS B 167 25.959 6.149 55.969 1.00 51.90 C

ATOM 2157 CE LYS B 167 26.838 5.240 55.122 1.00 59.30 C

ATOM 2158 NZ LYS B 167 27.619 4.292 55.967 1.00 52.77 N1+

ATOM 2159 H LYS B 167 22.432 8.179 54.687 1.00 0.00 H

ATOM 2160 HA LYS B 167 23.489 6.771 57.060 1.00 0.00 H

ATOM 2161 HB3 LYS B 167 25.281 8.564 56.862 1.00 0.00 H

ATOM 2162 HB2 LYS B 167 24.471 9.192 55.476 1.00 0.00 H

ATOM 2163 HG3 LYS B 167 26.218 7.836 54.675 1.00 0.00 H

ATOM 2164 HG2 LYS B 167 24.766 6.926 54.345 1.00 0.00 H

ATOM 2165 HD3 LYS B 167 25.164 5.551 56.413 1.00 0.00 H

ATOM 2166 HD2 LYS B 167 26.548 6.552 56.795 1.00 0.00 H

ATOM 2167 HE3 LYS B 167 27.549 5.830 54.543 1.00 0.00 H

ATOM 2168 HE2 LYS B 167 26.233 4.678 54.409 1.00 0.00 H

ATOM 2169 HZ1 LYS B 167 26.985 3.711 56.497 1.00 0.00 H

ATOM 2170 HZ2 LYS B 167 28.194 3.708 55.376 1.00 0.00 H

ATOM 2171 HZ3 LYS B 167 28.209 4.811 56.601 1.00 0.00 H

ATOM 2172 N SER B 168 21.888 9.566 57.282 1.00 44.58 N

ATOM 2173 CA SER B 168 21.275 10.473 58.244 1.00 41.14 C

ATOM 2174 C SER B 168 19.940 10.965 57.719 1.00 40.49 C

ATOM 2175 O SER B 168 19.508 10.572 56.639 1.00 34.58 O

ATOM 2176 CB SER B 168 22.188 11.667 58.535 1.00 38.70 C

ATOM 2177 OG SER B 168 22.054 12.665 57.538 1.00 38.25 O

ATOM 2178 H SER B 168 21.583 9.610 56.319 1.00 0.00 H

ATOM 2179 HA SER B 168 21.074 9.935 59.173 1.00 0.00 H

ATOM 2180 HB3 SER B 168 23.234 11.372 58.618 1.00 0.00 H

ATOM 2181 HB2 SER B 168 21.920 12.117 59.492 1.00 0.00 H

ATOM 2182 HG SER B 168 22.369 12.312 56.718 1.00 0.00 H

ATOM 2183 N HIS B 169 19.299 11.836 58.488 1.00 31.52 N

ATOM 2184 CA HIS B 169 18.006 12.408 58.128 1.00 33.08 C

ATOM 2185 C HIS B 169 18.161 13.601 57.188 1.00 32.10 C

ATOM 2186 O HIS B 169 17.177 14.182 56.727 1.00 29.57 O

ATOM 2187 CB HIS B 169 17.273 12.859 59.391 1.00 35.27 C

ATOM 2188 CG HIS B 169 18.073 13.790 60.249 1.00 36.09 C

ATOM 2189 ND1 HIS B 169 19.240 13.406 60.878 1.00 44.68 N

ATOM 2190 CD2 HIS B 169 17.874 15.086 60.586 1.00 36.86 C

ATOM 2191 CE1 HIS B 169 19.729 14.428 61.559 1.00 38.49 C

ATOM 2192 NE2 HIS B 169 18.917 15.458 61.403 1.00 39.18 N

ATOM 2193 H HIS B 169 19.708 12.126 59.367 1.00 0.00 H

ATOM 2194 HA HIS B 169 17.389 11.662 57.622 1.00 0.00 H

ATOM 2195 HB3 HIS B 169 17.018 11.987 59.994 1.00 0.00 H

ATOM 2196 HB2 HIS B 169 16.323 13.334 59.140 1.00 0.00 H

ATOM 2197 HD2 HIS B 169 17.085 15.772 60.315 1.00 0.00 H

ATOM 2198 HE1 HIS B 169 20.633 14.419 62.149 1.00 0.00 H

ATOM 2199 HE2 HIS B 169 19.044 16.368 61.823 1.00 0.00 H

ATOM 2200 N ARG B 170 19.400 13.985 56.922 1.00 29.99 N

ATOM 2201 CA ARG B 170 19.632 15.081 55.996 1.00 20.10 C

ATOM 2202 C ARG B 170 19.349 14.597 54.571 1.00 26.49 C

ATOM 2203 O ARG B 170 19.383 13.396 54.302 1.00 30.94 O

ATOM 2204 CB ARG B 170 21.066 15.592 56.131 1.00 26.78 C

ATOM 2205 CG ARG B 170 21.382 16.174 57.525 1.00 32.07 C

ATOM 2206 CD ARG B 170 22.747 16.863 57.565 1.00 36.35 C

ATOM 2207 NE ARG B 170 23.853 15.942 57.306 1.00 40.19 N

ATOM 2208 CZ ARG B 170 24.416 15.171 58.234 1.00 46.21 C

ATOM 2209 NH1 ARG B 170 23.974 15.207 59.485 1.00 41.14 N

ATOM 2210 NH2 ARG B 170 25.415 14.357 57.909 1.00 45.56 N1+

ATOM 2211 H ARG B 170 20.190 13.480 57.303 1.00 0.00 H

ATOM 2212 HA ARG B 170 18.958 15.911 56.224 1.00 0.00 H

ATOM 2213 HB3 ARG B 170 21.244 16.365 55.382 1.00 0.00 H

ATOM 2214 HB2 ARG B 170 21.769 14.793 55.893 1.00 0.00 H

ATOM 2215 HG3 ARG B 170 21.334 15.393 58.284 1.00 0.00 H

ATOM 2216 HG2 ARG B 170 20.616 16.904 57.791 1.00 0.00 H

ATOM 2217 HD3 ARG B 170 22.893 17.371 58.518 1.00 0.00 H

ATOM 2218 HD2 ARG B 170 22.780 17.646 56.806 1.00 0.00 H

ATOM 2219 HE ARG B 170 24.159 15.801 56.342 1.00 0.00 H

ATOM 2220 HH12 ARG B 170 24.391 14.622 60.194 1.00 0.00 H

ATOM 2221 HH11 ARG B 170 23.204 15.812 59.729 1.00 0.00 H

ATOM 2222 HH22 ARG B 170 25.858 13.759 58.590 1.00 0.00 H

ATOM 2223 HH21 ARG B 170 25.691 14.311 56.932 1.00 0.00 H

ATOM 2224 N LEU B 171 19.035 15.531 53.676 1.00 17.23 N

ATOM 2225 CA LEU B 171 18.966 15.238 52.241 1.00 15.67 C

ATOM 2226 C LEU B 171 20.268 15.668 51.585 1.00 15.44 C

ATOM 2227 O LEU B 171 21.049 16.428 52.160 1.00 16.68 O

ATOM 2228 CB LEU B 171 17.814 15.998 51.589 1.00 15.49 C

ATOM 2229 CG LEU B 171 16.401 15.697 52.086 1.00 18.70 C

ATOM 2230 CD1 LEU B 171 15.414 16.517 51.295 1.00 19.03 C

ATOM 2231 CD2 LEU B 171 16.076 14.210 51.999 1.00 19.46 C

ATOM 2232 H LEU B 171 19.020 16.502 53.962 1.00 0.00 H

ATOM 2233 HA LEU B 171 18.840 14.172 52.052 1.00 0.00 H

ATOM 2234 HB3 LEU B 171 17.837 15.818 50.512 1.00 0.00 H

ATOM 2235 HB2 LEU B 171 17.991 17.068 51.710 1.00 0.00 H

ATOM 2236 HG LEU B 171 16.323 15.994 53.133 1.00 0.00 H

ATOM 2237 HD11 LEU B 171 14.398 16.237 51.538 1.00 0.00 H

ATOM 2238 HD12 LEU B 171 15.532 17.582 51.494 1.00 0.00 H

ATOM 2239 HD13 LEU B 171 15.540 16.364 50.224 1.00 0.00 H

ATOM 2240 HD21 LEU B 171 15.038 14.022 52.276 1.00 0.00 H

ATOM 2241 HD22 LEU B 171 16.218 13.834 50.986 1.00 0.00 H

ATOM 2242 HD23 LEU B 171 16.688 13.633 52.691 1.00 0.00 H

ATOM 2243 N ILE B 172 20.489 15.197 50.360 1.00 15.49 N

ATOM 2244 CA ILE B 172 21.680 15.561 49.608 1.00 12.32 C

ATOM 2245 C ILE B 172 21.260 16.193 48.291 1.00 13.70 C

ATOM 2246 O ILE B 172 20.521 15.588 47.525 1.00 15.20 O

ATOM 2247 CB ILE B 172 22.541 14.320 49.319 1.00 12.86 C

ATOM 2248 CG1 ILE B 172 22.848 13.584 50.630 1.00 20.24 C

ATOM 2249 CG2 ILE B 172 23.802 14.704 48.564 1.00 15.10 C

ATOM 2250 CD1 ILE B 172 23.382 12.186 50.430 1.00 25.31 C

ATOM 2251 H ILE B 172 19.831 14.549 49.942 1.00 0.00 H

ATOM 2252 HA ILE B 172 22.306 16.278 50.140 1.00 0.00 H

ATOM 2253 HB ILE B 172 21.967 13.638 48.693 1.00 0.00 H

ATOM 2254 HG13 ILE B 172 21.984 13.481 51.283 1.00 0.00 H

ATOM 2255 HG12 ILE B 172 23.598 14.102 51.181 1.00 0.00 H

ATOM 2256 HG21 ILE B 172 24.415 13.828 48.378 1.00 0.00 H

ATOM 2257 HG22 ILE B 172 23.608 15.146 47.587 1.00 0.00 H

ATOM 2258 HG23 ILE B 172 24.395 15.411 49.145 1.00 0.00 H

ATOM 2259 HD11 ILE B 172 23.554 11.699 51.390 1.00 0.00 H

ATOM 2260 HD12 ILE B 172 22.675 11.571 49.872 1.00 0.00 H

ATOM 2261 HD13 ILE B 172 24.330 12.179 49.893 1.00 0.00 H

ATOM 2262 N ALA B 173 21.690 17.430 48.066 1.00 11.93 N

ATOM 2263 CA ALA B 173 21.492 18.094 46.785 1.00 12.36 C

ATOM 2264 C ALA B 173 22.683 17.779 45.908 1.00 13.21 C

ATOM 2265 O ALA B 173 23.812 18.072 46.267 1.00 13.89 O

ATOM 2266 CB ALA B 173 21.367 19.597 46.980 1.00 13.42 C

ATOM 2267 H ALA B 173 22.273 17.891 48.755 1.00 0.00 H

ATOM 2268 HA ALA B 173 20.575 17.752 46.300 1.00 0.00 H

ATOM 2269 HB1 ALA B 173 21.276 20.112 46.023 1.00 0.00 H

ATOM 2270 HB2 ALA B 173 20.481 19.850 47.560 1.00 0.00 H

ATOM 2271 HB3 ALA B 173 22.230 20.015 47.500 1.00 0.00 H

ATOM 2272 N HIS B 174 22.426 17.150 44.766 1.00 11.99 N

ATOM 2273 CA HIS B 174 23.479 16.852 43.813 1.00 12.67 C

ATOM 2274 C HIS B 174 23.567 17.983 42.808 1.00 13.21 C

ATOM 2275 O HIS B 174 22.667 18.154 41.980 1.00 14.52 O

ATOM 2276 CB HIS B 174 23.170 15.545 43.092 1.00 13.91 C

ATOM 2277 CG HIS B 174 23.293 14.344 43.967 1.00 13.69 C

ATOM 2278 ND1 HIS B 174 24.296 13.412 43.807 1.00 14.79 N1+

ATOM 2279 CD2 HIS B 174 22.549 13.925 45.016 1.00 14.15 C

ATOM 2280 CE1 HIS B 174 24.166 12.472 44.727 1.00 15.05 C

ATOM 2281 NE2 HIS B 174 23.116 12.759 45.475 1.00 16.77 N

ATOM 2282 H HIS B 174 21.472 16.923 44.514 1.00 0.00 H

ATOM 2283 HA HIS B 174 24.450 16.734 44.299 1.00 0.00 H

ATOM 2284 HB3 HIS B 174 23.835 15.423 42.244 1.00 0.00 H

ATOM 2285 HB2 HIS B 174 22.165 15.556 42.671 1.00 0.00 H

ATOM 2286 HD1 HIS B 174 24.993 13.397 43.059 1.00 0.00 H

ATOM 2287 HD2 HIS B 174 21.667 14.338 45.482 1.00 0.00 H

ATOM 2288 HE1 HIS B 174 24.789 11.595 44.826 1.00 0.00 H

ATOM 2289 HE2 HIS B 174 22.767 12.154 46.218 1.00 0.00 H

ATOM 2290 N LEU B 175 24.632 18.770 42.894 1.00 12.51 N

ATOM 2291 CA LEU B 175 24.835 19.881 41.983 1.00 14.62 C

ATOM 2292 C LEU B 175 25.798 19.435 40.894 1.00 17.38 C

ATOM 2293 O LEU B 175 26.250 18.297 40.899 1.00 17.67 O

ATOM 2294 CB LEU B 175 25.391 21.102 42.725 1.00 15.22 C

ATOM 2295 CG LEU B 175 24.616 21.520 43.977 1.00 16.21 C

ATOM 2296 CD1 LEU B 175 25.164 22.834 44.550 1.00 20.60 C

ATOM 2297 CD2 LEU B 175 23.129 21.633 43.716 1.00 19.43 C

ATOM 2298 H LEU B 175 25.359 18.574 43.571 1.00 0.00 H

ATOM 2299 HA LEU B 175 23.907 20.160 41.493 1.00 0.00 H

ATOM 2300 HB3 LEU B 175 25.411 21.950 42.038 1.00 0.00 H

ATOM 2301 HB2 LEU B 175 26.431 20.926 43.006 1.00 0.00 H

ATOM 2302 HG LEU B 175 24.757 20.752 44.740 1.00 0.00 H

ATOM 2303 HD11 LEU B 175 24.638 23.111 45.463 1.00 0.00 H

ATOM 2304 HD12 LEU B 175 26.223 22.746 44.795 1.00 0.00 H

ATOM 2305 HD13 LEU B 175 25.052 23.656 43.842 1.00 0.00 H

ATOM 2306 HD21 LEU B 175 22.606 21.963 44.614 1.00 0.00 H

ATOM 2307 HD22 LEU B 175 22.916 22.364 42.937 1.00 0.00 H

ATOM 2308 HD23 LEU B 175 22.675 20.686 43.431 1.00 0.00 H

ATOM 2309 N ASP B 176 26.119 20.337 39.973 1.00 19.20 N

ATOM 2310 CA AASP B 176 26.961 19.975 38.845 0.62 21.72 C

ATOM 2311 CA BASP B 176 26.993 20.024 38.843 0.38 21.74 C

ATOM 2312 C ASP B 176 28.274 19.316 39.260 1.00 21.27 C

ATOM 2313 O ASP B 176 28.583 18.226 38.783 1.00 22.75 O

ATOM 2314 CB AASP B 176 27.226 21.179 37.949 0.62 22.19 C

ATOM 2315 CB BASP B 176 27.355 21.296 38.074 0.38 22.31 C

ATOM 2316 CG AASP B 176 27.934 20.797 36.672 0.62 28.21 C

ATOM 2317 CG BASP B 176 26.260 21.739 37.127 0.38 23.54 C

ATOM 2318 OD1AASP B 176 27.315 20.100 35.837 0.62 26.19 O

ATOM 2319 OD1BASP B 176 25.985 21.004 36.156 0.38 28.96 O

ATOM 2320 OD2AASP B 176 29.109 21.188 36.509 0.62 33.68 O1-

ATOM 2321 OD2BASP B 176 25.686 22.827 37.347 0.38 29.29 O1-

ATOM 2322 H ASP B 176 25.717 21.263 40.007 1.00 0.00 H

ATOM 2323 HA AASP B 176 26.394 19.242 38.266 0.62 0.00 H

ATOM 2324 HA BASP B 176 26.448 19.365 38.167 0.38 0.00 H

ATOM 2325 HB3AASP B 176 27.803 21.940 38.477 0.62 0.00 H

ATOM 2326 HB3BASP B 176 28.274 21.130 37.512 0.38 0.00 H

ATOM 2327 HB2AASP B 176 26.281 21.649 37.674 0.62 0.00 H

ATOM 2328 HB2BASP B 176 27.569 22.098 38.781 0.38 0.00 H

ATOM 2329 N ASN B 177 29.036 19.949 40.156 1.00 21.03 N

ATOM 2330 CA ASN B 177 30.325 19.370 40.542 1.00 26.18 C

ATOM 2331 C ASN B 177 30.503 19.035 42.019 1.00 19.27 C

ATOM 2332 O ASN B 177 31.610 18.743 42.464 1.00 25.34 O

ATOM 2333 CB ASN B 177 31.487 20.251 40.061 1.00 31.07 C

ATOM 2334 CG ASN B 177 32.795 19.475 39.942 1.00 44.20 C

ATOM 2335 OD1 ASN B 177 32.813 18.476 39.064 1.00 43.80 O

ATOM 2336 ND2 ASN B 177 33.768 19.759 40.645 1.00 44.30 N

ATOM 2337 H ASN B 177 28.754 20.843 40.530 1.00 0.00 H

ATOM 2338 HA ASN B 177 30.466 18.391 40.084 1.00 0.00 H

ATOM 2339 HB3 ASN B 177 31.608 21.126 40.702 1.00 0.00 H

ATOM 2340 HB2 ASN B 177 31.263 20.637 39.065 1.00 0.00 H

ATOM 2341 HD22 ASN B 177 34.616 19.219 40.555 1.00 0.00 H

ATOM 2342 HD21 ASN B 177 33.706 20.508 41.317 1.00 0.00 H

ATOM 2343 N ARG B 178 29.417 19.047 42.782 1.00 18.01 N

ATOM 2344 CA ARG B 178 29.513 18.707 44.192 1.00 19.07 C

ATOM 2345 C ARG B 178 28.161 18.332 44.767 1.00 18.32 C

ATOM 2346 O ARG B 178 27.121 18.579 44.157 1.00 17.05 O

ATOM 2347 CB ARG B 178 30.076 19.889 44.978 1.00 16.50 C

ATOM 2348 CG ARG B 178 29.195 21.108 44.915 1.00 18.17 C

ATOM 2349 CD ARG B 178 29.606 22.128 45.956 1.00 19.50 C

ATOM 2350 NE ARG B 178 31.005 22.539 45.843 1.00 21.41 N

ATOM 2351 CZ ARG B 178 31.755 22.902 46.883 1.00 22.36 C

ATOM 2352 NH1 ARG B 178 33.013 23.279 46.700 1.00 24.67 N

ATOM 2353 NH2 ARG B 178 31.243 22.884 48.109 1.00 24.57 N1+

ATOM 2354 H ARG B 178 28.508 19.239 42.384 1.00 0.00 H

ATOM 2355 HA ARG B 178 30.163 17.838 44.320 1.00 0.00 H

ATOM 2356 HB3 ARG B 178 31.079 20.134 44.629 1.00 0.00 H

ATOM 2357 HB2 ARG B 178 30.206 19.590 46.020 1.00 0.00 H

ATOM 2358 HG3 ARG B 178 28.144 20.892 45.099 1.00 0.00 H

ATOM 2359 HG2 ARG B 178 29.238 21.555 43.921 1.00 0.00 H

ATOM 2360 HD3 ARG B 178 29.401 21.696 46.935 1.00 0.00 H

ATOM 2361 HD2 ARG B 178 28.973 23.010 45.873 1.00 0.00 H

ATOM 2362 HE ARG B 178 31.384 22.603 44.909 1.00 0.00 H

ATOM 2363 HH12 ARG B 178 33.569 23.559 47.497 1.00 0.00 H

ATOM 2364 HH11 ARG B 178 33.409 23.313 45.774 1.00 0.00 H

ATOM 2365 HH22 ARG B 178 31.859 23.039 48.904 1.00 0.00 H

ATOM 2366 HH21 ARG B 178 30.287 22.604 48.266 1.00 0.00 H

ATOM 2367 N GLN B 179 28.192 17.715 45.941 1.00 15.02 N

ATOM 2368 CA GLN B 179 26.996 17.428 46.714 1.00 17.02 C

ATOM 2369 C GLN B 179 26.972 18.322 47.940 1.00 18.95 C

ATOM 2370 O GLN B 179 28.024 18.648 48.492 1.00 20.45 O

ATOM 2371 CB GLN B 179 26.988 15.963 47.139 1.00 18.58 C

ATOM 2372 CG GLN B 179 26.926 15.027 45.956 1.00 19.47 C

ATOM 2373 CD GLN B 179 27.048 13.574 46.349 1.00 26.60 C

ATOM 2374 OE1 GLN B 179 26.616 13.164 47.428 1.00 32.46 O

ATOM 2375 NE2 GLN B 179 27.639 12.779 45.467 1.00 32.63 N

ATOM 2376 H GLN B 179 29.083 17.536 46.381 1.00 0.00 H

ATOM 2377 HA GLN B 179 26.109 17.607 46.119 1.00 0.00 H

ATOM 2378 HB3 GLN B 179 26.138 15.783 47.792 1.00 0.00 H

ATOM 2379 HB2 GLN B 179 27.878 15.748 47.734 1.00 0.00 H

ATOM 2380 HG3 GLN B 179 27.716 15.257 45.240 1.00 0.00 H

ATOM 2381 HG2 GLN B 179 25.982 15.163 45.428 1.00 0.00 H

ATOM 2382 HE22 GLN B 179 27.751 11.798 45.678 1.00 0.00 H

ATOM 2383 HE21 GLN B 179 27.963 13.150 44.585 1.00 0.00 H

ATOM 2384 N ILE B 180 25.776 18.734 48.350 1.00 14.30 N

ATOM 2385 CA ILE B 180 25.606 19.549 49.552 1.00 16.00 C

ATOM 2386 C ILE B 180 24.504 18.947 50.393 1.00 15.59 C

ATOM 2387 O ILE B 180 23.375 18.813 49.919 1.00 15.53 O

ATOM 2388 CB ILE B 180 25.209 21.002 49.191 1.00 12.63 C

ATOM 2389 CG1 ILE B 180 26.344 21.684 48.419 1.00 19.28 C

ATOM 2390 CG2 ILE B 180 24.816 21.785 50.474 1.00 17.53 C

ATOM 2391 CD1 ILE B 180 26.128 23.162 48.155 1.00 24.41 C

ATOM 2392 H ILE B 180 24.949 18.456 47.836 1.00 0.00 H

ATOM 2393 HA ILE B 180 26.512 19.573 50.161 1.00 0.00 H

ATOM 2394 HB ILE B 180 24.334 20.986 48.539 1.00 0.00 H

ATOM 2395 HG13 ILE B 180 26.470 21.189 47.455 1.00 0.00 H

ATOM 2396 HG12 ILE B 180 27.289 21.558 48.949 1.00 0.00 H

ATOM 2397 HG21 ILE B 180 24.587 22.828 50.258 1.00 0.00 H

ATOM 2398 HG22 ILE B 180 23.911 21.405 50.947 1.00 0.00 H

ATOM 2399 HG23 ILE B 180 25.624 21.774 51.204 1.00 0.00 H

ATOM 2400 HD11 ILE B 180 26.840 23.527 47.414 1.00 0.00 H

ATOM 2401 HD12 ILE B 180 25.124 23.361 47.780 1.00 0.00 H

ATOM 2402 HD13 ILE B 180 26.286 23.747 49.060 1.00 0.00 H

ATOM 2403 N GLU B 181 24.817 18.570 51.636 1.00 15.94 N

ATOM 2404 CA GLU B 181 23.805 18.014 52.528 1.00 14.59 C

ATOM 2405 C GLU B 181 23.061 19.132 53.226 1.00 17.72 C

ATOM 2406 O GLU B 181 23.649 20.161 53.544 1.00 17.61 O

ATOM 2407 CB GLU B 181 24.435 17.079 53.567 1.00 21.06 C

ATOM 2408 CG GLU B 181 25.071 15.831 52.961 1.00 29.35 C

ATOM 2409 CD GLU B 181 25.346 14.740 53.992 1.00 40.81 C

ATOM 2410 OE1 GLU B 181 24.982 14.915 55.177 1.00 40.03 O

ATOM 2411 OE2 GLU B 181 25.927 13.702 53.607 1.00 45.99 O1-

ATOM 2412 H GLU B 181 25.761 18.677 51.978 1.00 0.00 H

ATOM 2413 HA GLU B 181 23.120 17.429 51.931 1.00 0.00 H

ATOM 2414 HB3 GLU B 181 23.641 16.783 54.256 1.00 0.00 H

ATOM 2415 HB2 GLU B 181 25.173 17.615 54.166 1.00 0.00 H

ATOM 2416 HG3 GLU B 181 26.005 16.088 52.460 1.00 0.00 H

ATOM 2417 HG2 GLU B 181 24.406 15.489 52.201 1.00 0.00 H

ATOM 2418 N PHE B 182 21.770 18.937 53.445 1.00 14.18 N

ATOM 2419 CA PHE B 182 20.941 19.987 54.040 1.00 15.29 C

ATOM 2420 C PHE B 182 19.704 19.374 54.685 1.00 15.11 C

ATOM 2421 O PHE B 182 19.490 18.162 54.613 1.00 15.88 O

ATOM 2422 CB PHE B 182 20.539 21.027 52.979 1.00 13.30 C

ATOM 2423 CG PHE B 182 19.571 20.494 51.958 1.00 13.50 C

ATOM 2424 CD1 PHE B 182 18.213 20.783 52.044 1.00 13.73 C

ATOM 2425 CD2 PHE B 182 20.014 19.682 50.926 1.00 13.71 C

ATOM 2426 CE1 PHE B 182 17.313 20.275 51.123 1.00 13.85 C

ATOM 2427 CE2 PHE B 182 19.116 19.178 49.984 1.00 12.59 C

ATOM 2428 CZ PHE B 182 17.767 19.470 50.083 1.00 13.87 C

ATOM 2429 H PHE B 182 21.329 18.071 53.160 1.00 0.00 H

ATOM 2430 HA PHE B 182 21.488 20.475 54.847 1.00 0.00 H

ATOM 2431 HB3 PHE B 182 21.423 21.400 52.461 1.00 0.00 H

ATOM 2432 HB2 PHE B 182 20.092 21.904 53.439 1.00 0.00 H

ATOM 2433 HD1 PHE B 182 17.849 21.418 52.838 1.00 0.00 H

ATOM 2434 HD2 PHE B 182 21.061 19.437 50.838 1.00 0.00 H

ATOM 2435 HE1 PHE B 182 16.262 20.509 51.207 1.00 0.00 H

ATOM 2436 HE2 PHE B 182 19.469 18.539 49.191 1.00 0.00 H

ATOM 2437 HZ PHE B 182 17.077 19.068 49.360 1.00 0.00 H

ATOM 2438 N TYR B 183 18.887 20.218 55.301 1.00 13.68 N

ATOM 2439 CA TYR B 183 17.685 19.754 55.983 1.00 14.67 C

ATOM 2440 C TYR B 183 16.448 20.086 55.179 1.00 18.12 C

ATOM 2441 O TYR B 183 16.172 21.250 54.906 1.00 18.22 O

ATOM 2442 CB TYR B 183 17.580 20.421 57.356 1.00 16.42 C

ATOM 2443 CG TYR B 183 18.617 19.944 58.334 1.00 17.57 C

ATOM 2444 CD1 TYR B 183 19.926 20.389 58.259 1.00 18.09 C

ATOM 2445 CD2 TYR B 183 18.278 19.048 59.335 1.00 23.27 C

ATOM 2446 CE1 TYR B 183 20.886 19.953 59.154 1.00 22.69 C

ATOM 2447 CE2 TYR B 183 19.226 18.601 60.237 1.00 24.65 C

ATOM 2448 CZ TYR B 183 20.526 19.056 60.138 1.00 26.71 C

ATOM 2449 OH TYR B 183 21.469 18.617 61.037 1.00 32.21 O

ATOM 2450 H TYR B 183 19.106 21.206 55.328 1.00 0.00 H

ATOM 2451 HA TYR B 183 17.701 18.675 56.158 1.00 0.00 H

ATOM 2452 HB3 TYR B 183 16.594 20.241 57.788 1.00 0.00 H

ATOM 2453 HB2 TYR B 183 17.666 21.505 57.262 1.00 0.00 H

ATOM 2454 HD1 TYR B 183 20.213 21.093 57.494 1.00 0.00 H

ATOM 2455 HD2 TYR B 183 17.261 18.693 59.422 1.00 0.00 H

ATOM 2456 HE1 TYR B 183 21.901 20.315 59.077 1.00 0.00 H

ATOM 2457 HE2 TYR B 183 18.939 17.938 61.033 1.00 0.00 H

ATOM 2458 HH TYR B 183 22.308 19.040 60.933 1.00 0.00 H

ATOM 2459 N GLY B 184 15.678 19.068 54.821 1.00 16.22 N

ATOM 2460 CA GLY B 184 14.475 19.301 54.046 1.00 16.47 C

ATOM 2461 C GLY B 184 13.723 18.006 53.887 1.00 19.69 C

ATOM 2462 O GLY B 184 14.069 16.998 54.506 1.00 20.68 O

ATOM 2463 H GLY B 184 15.914 18.118 55.073 1.00 0.00 H

ATOM 2464 HA3 GLY B 184 14.734 19.696 53.062 1.00 0.00 H

ATOM 2465 HA2 GLY B 184 13.828 20.026 54.543 1.00 0.00 H

ATOM 2466 N ASN B 185 12.684 18.025 53.067 1.00 20.72 N

ATOM 2467 CA ASN B 185 12.019 16.774 52.738 1.00 20.89 C

ATOM 2468 C ASN B 185 11.669 16.674 51.266 1.00 16.81 C

ATOM 2469 O ASN B 185 11.322 17.669 50.623 1.00 18.32 O

ATOM 2470 CB ASN B 185 10.796 16.529 53.620 1.00 25.66 C

ATOM 2471 CG ASN B 185 9.655 17.446 53.297 1.00 23.54 C

ATOM 2472 OD1 ASN B 185 8.705 16.959 52.501 1.00 33.69 O

ATOM 2473 ND2 ASN B 185 9.611 18.582 53.772 1.00 19.18 N

ATOM 2474 H ASN B 185 12.420 18.867 52.574 1.00 0.00 H

ATOM 2475 HA ASN B 185 12.681 15.926 52.926 1.00 0.00 H

ATOM 2476 HB3 ASN B 185 11.060 16.628 54.674 1.00 0.00 H

ATOM 2477 HB2 ASN B 185 10.454 15.500 53.498 1.00 0.00 H

ATOM 2478 HD22 ASN B 185 8.808 19.174 53.603 1.00 0.00 H

ATOM 2479 HD21 ASN B 185 10.352 18.911 54.373 1.00 0.00 H

ATOM 2480 N LEU B 186 11.770 15.457 50.749 1.00 18.11 N

ATOM 2481 CA LEU B 186 11.606 15.191 49.321 1.00 17.41 C

ATOM 2482 C LEU B 186 10.239 15.606 48.789 1.00 18.42 C

ATOM 2483 O LEU B 186 10.145 16.164 47.700 1.00 16.47 O

ATOM 2484 CB LEU B 186 11.853 13.709 49.026 1.00 17.09 C

ATOM 2485 CG LEU B 186 13.287 13.211 49.200 1.00 17.28 C

ATOM 2486 CD1 LEU B 186 13.380 11.736 48.873 1.00 18.96 C

ATOM 2487 CD2 LEU B 186 14.266 14.014 48.335 1.00 17.10 C

ATOM 2488 H LEU B 186 12.052 14.691 51.343 1.00 0.00 H

ATOM 2489 HA LEU B 186 12.326 15.804 48.780 1.00 0.00 H

ATOM 2490 HB3 LEU B 186 11.541 13.489 48.005 1.00 0.00 H

ATOM 2491 HB2 LEU B 186 11.195 13.108 49.657 1.00 0.00 H

ATOM 2492 HG LEU B 186 13.579 13.338 50.243 1.00 0.00 H

ATOM 2493 HD11 LEU B 186 14.398 11.366 48.996 1.00 0.00 H

ATOM 2494 HD12 LEU B 186 12.737 11.145 49.525 1.00 0.00 H

ATOM 2495 HD13 LEU B 186 13.084 11.535 47.843 1.00 0.00 H

ATOM 2496 HD21 LEU B 186 15.271 13.594 48.402 1.00 0.00 H

ATOM 2497 HD22 LEU B 186 13.988 14.004 47.283 1.00 0.00 H

ATOM 2498 HD23 LEU B 186 14.351 15.051 48.654 1.00 0.00 H

ATOM 2499 N LYS B 187 9.179 15.348 49.552 1.00 18.82 N

ATOM 2500 CA LYS B 187 7.841 15.672 49.069 1.00 17.53 C

ATOM 2501 C LYS B 187 7.673 17.164 48.805 1.00 16.93 C

ATOM 2502 O LYS B 187 7.180 17.559 47.746 1.00 20.20 O

ATOM 2503 CB LYS B 187 6.769 15.169 50.041 1.00 24.71 C

ATOM 2504 CG LYS B 187 5.351 15.328 49.508 1.00 28.31 C

ATOM 2505 CD LYS B 187 4.366 14.503 50.316 1.00 32.96 C

ATOM 2506 CE LYS B 187 2.988 14.513 49.677 1.00 43.20 C

ATOM 2507 NZ LYS B 187 2.005 13.709 50.461 1.00 47.24 N1+

ATOM 2508 H LYS B 187 9.292 14.930 50.464 1.00 0.00 H

ATOM 2509 HA LYS B 187 7.702 15.146 48.122 1.00 0.00 H

ATOM 2510 HB3 LYS B 187 6.857 15.665 51.008 1.00 0.00 H

ATOM 2511 HB2 LYS B 187 6.961 14.112 50.230 1.00 0.00 H

ATOM 2512 HG3 LYS B 187 5.318 15.007 48.466 1.00 0.00 H

ATOM 2513 HG2 LYS B 187 5.056 16.377 49.523 1.00 0.00 H

ATOM 2514 HD3 LYS B 187 4.313 14.889 51.335 1.00 0.00 H

ATOM 2515 HD2 LYS B 187 4.716 13.472 50.388 1.00 0.00 H

ATOM 2516 HE3 LYS B 187 3.033 14.115 48.663 1.00 0.00 H

ATOM 2517 HE2 LYS B 187 2.612 15.533 49.606 1.00 0.00 H

ATOM 2518 HZ1 LYS B 187 2.317 12.750 50.515 1.00 0.00 H

ATOM 2519 HZ2 LYS B 187 1.925 14.088 51.394 1.00 0.00 H

ATOM 2520 HZ3 LYS B 187 1.103 13.742 50.008 1.00 0.00 H

ATOM 2521 N GLU B 188 8.097 18.004 49.750 1.00 17.56 N

ATOM 2522 CA GLU B 188 7.986 19.445 49.552 1.00 16.29 C

ATOM 2523 C GLU B 188 8.814 19.902 48.367 1.00 17.30 C

ATOM 2524 O GLU B 188 8.368 20.723 47.569 1.00 18.60 O

ATOM 2525 CB GLU B 188 8.417 20.217 50.807 1.00 21.86 C

ATOM 2526 CG GLU B 188 7.463 20.083 51.974 1.00 33.59 C

ATOM 2527 CD GLU B 188 7.911 20.894 53.179 1.00 44.52 C

ATOM 2528 OE1 GLU B 188 8.749 21.803 53.000 1.00 45.35 O

ATOM 2529 OE2 GLU B 188 7.436 20.613 54.301 1.00 45.46 O1-

ATOM 2530 H GLU B 188 8.489 17.653 50.615 1.00 0.00 H

ATOM 2531 HA GLU B 188 6.944 19.691 49.342 1.00 0.00 H

ATOM 2532 HB3 GLU B 188 8.503 21.274 50.546 1.00 0.00 H

ATOM 2533 HB2 GLU B 188 9.418 19.898 51.106 1.00 0.00 H

ATOM 2534 HG3 GLU B 188 7.315 19.044 52.258 1.00 0.00 H

ATOM 2535 HG2 GLU B 188 6.480 20.452 51.679 1.00 0.00 H

ATOM 2536 N LEU B 189 10.022 19.359 48.247 1.00 18.38 N

ATOM 2537 CA LEU B 189 10.879 19.719 47.127 1.00 16.49 C

ATOM 2538 C LEU B 189 10.241 19.350 45.795 1.00 16.57 C

ATOM 2539 O LEU B 189 10.334 20.109 44.817 1.00 19.10 O

ATOM 2540 CB LEU B 189 12.249 19.050 47.260 1.00 15.27 C

ATOM 2541 CG LEU B 189 13.082 19.533 48.448 1.00 18.15 C

ATOM 2542 CD1 LEU B 189 14.340 18.683 48.592 1.00 17.40 C

ATOM 2543 CD2 LEU B 189 13.441 20.967 48.232 1.00 20.16 C

ATOM 2544 H LEU B 189 10.353 18.693 48.932 1.00 0.00 H

ATOM 2545 HA LEU B 189 11.001 20.800 47.116 1.00 0.00 H

ATOM 2546 HB3 LEU B 189 12.822 19.196 46.342 1.00 0.00 H

ATOM 2547 HB2 LEU B 189 12.109 17.971 47.341 1.00 0.00 H

ATOM 2548 HG LEU B 189 12.509 19.457 49.370 1.00 0.00 H

ATOM 2549 HD11 LEU B 189 14.873 18.950 49.503 1.00 0.00 H

ATOM 2550 HD12 LEU B 189 14.100 17.622 48.653 1.00 0.00 H

ATOM 2551 HD13 LEU B 189 15.005 18.832 47.749 1.00 0.00 H

ATOM 2552 HD21 LEU B 189 14.166 21.298 48.976 1.00 0.00 H

ATOM 2553 HD22 LEU B 189 13.888 21.136 47.251 1.00 0.00 H

ATOM 2554 HD23 LEU B 189 12.583 21.631 48.333 1.00 0.00 H

ATOM 2555 N SER B 190 9.584 18.196 45.751 1.00 16.91 N

ATOM 2556 CA SER B 190 8.948 17.751 44.520 1.00 19.53 C

ATOM 2557 C SER B 190 7.780 18.661 44.154 1.00 20.69 C

ATOM 2558 O SER B 190 7.357 18.708 43.001 1.00 24.45 O

ATOM 2559 CB SER B 190 8.483 16.296 44.628 1.00 18.04 C

ATOM 2560 OG SER B 190 7.328 16.170 45.443 1.00 20.98 O

ATOM 2561 H SER B 190 9.553 17.599 46.568 1.00 0.00 H

ATOM 2562 HA SER B 190 9.683 17.801 43.715 1.00 0.00 H

ATOM 2563 HB3 SER B 190 9.277 15.671 45.035 1.00 0.00 H

ATOM 2564 HB2 SER B 190 8.249 15.900 43.639 1.00 0.00 H

ATOM 2565 HG SER B 190 7.479 16.613 46.268 1.00 0.00 H

ATOM 2566 N GLN B 191 7.271 19.397 45.135 1.00 19.81 N

ATOM 2567 CA GLN B 191 6.117 20.262 44.919 1.00 19.58 C

ATOM 2568 C GLN B 191 6.500 21.681 44.478 1.00 22.44 C

ATOM 2569 O GLN B 191 5.636 22.480 44.110 1.00 21.92 O

ATOM 2570 CB GLN B 191 5.235 20.278 46.175 1.00 19.65 C

ATOM 2571 CG GLN B 191 4.536 18.946 46.431 1.00 23.61 C

ATOM 2572 CD GLN B 191 3.982 18.806 47.842 1.00 27.31 C

ATOM 2573 OE1 GLN B 191 4.309 19.587 48.743 1.00 26.36 O

ATOM 2574 NE2 GLN B 191 3.132 17.802 48.039 1.00 34.48 N

ATOM 2575 H GLN B 191 7.665 19.343 46.064 1.00 0.00 H

ATOM 2576 HA GLN B 191 5.492 19.866 44.116 1.00 0.00 H

ATOM 2577 HB3 GLN B 191 4.471 21.053 46.092 1.00 0.00 H

ATOM 2578 HB2 GLN B 191 5.853 20.564 47.026 1.00 0.00 H

ATOM 2579 HG3 GLN B 191 5.213 18.111 46.257 1.00 0.00 H

ATOM 2580 HG2 GLN B 191 3.724 18.824 45.712 1.00 0.00 H

ATOM 2581 HE22 GLN B 191 2.718 17.680 48.951 1.00 0.00 H

ATOM 2582 HE21 GLN B 191 2.884 17.194 47.272 1.00 0.00 H

ATOM 2583 N LEU B 192 7.794 21.980 44.470 1.00 21.29 N

ATOM 2584 CA LEU B 192 8.259 23.338 44.184 1.00 19.14 C

ATOM 2585 C LEU B 192 7.995 23.821 42.770 1.00 19.78 C

ATOM 2586 O LEU B 192 7.755 25.002 42.555 1.00 20.30 O

ATOM 2587 CB LEU B 192 9.761 23.471 44.449 1.00 21.16 C

ATOM 2588 CG LEU B 192 10.273 23.595 45.882 1.00 29.29 C

ATOM 2589 CD1 LEU B 192 11.789 23.678 45.858 1.00 29.58 C

ATOM 2590 CD2 LEU B 192 9.694 24.816 46.575 1.00 26.51 C

ATOM 2591 H LEU B 192 8.472 21.284 44.750 1.00 0.00 H

ATOM 2592 HA LEU B 192 7.718 24.022 44.838 1.00 0.00 H

ATOM 2593 HB3 LEU B 192 10.138 24.351 43.922 1.00 0.00 H

ATOM 2594 HB2 LEU B 192 10.268 22.634 43.966 1.00 0.00 H

ATOM 2595 HG LEU B 192 9.975 22.710 46.442 1.00 0.00 H

ATOM 2596 HD11 LEU B 192 12.200 23.781 46.863 1.00 0.00 H

ATOM 2597 HD12 LEU B 192 12.226 22.780 45.420 1.00 0.00 H

ATOM 2598 HD13 LEU B 192 12.125 24.531 45.269 1.00 0.00 H

ATOM 2599 HD21 LEU B 192 10.142 24.953 47.560 1.00 0.00 H

ATOM 2600 HD22 LEU B 192 9.875 25.726 46.001 1.00 0.00 H

ATOM 2601 HD23 LEU B 192 8.620 24.723 46.732 1.00 0.00 H

ATOM 2602 N ASP B 193 8.088 22.918 41.799 1.00 18.39 N

ATOM 2603 CA ASP B 193 8.012 23.301 40.402 1.00 22.01 C

ATOM 2604 C ASP B 193 7.731 22.038 39.605 1.00 17.43 C

ATOM 2605 O ASP B 193 8.041 20.939 40.063 1.00 19.83 O

ATOM 2606 CB ASP B 193 9.339 23.928 39.961 1.00 21.14 C

ATOM 2607 CG ASP B 193 9.284 24.511 38.557 1.00 24.39 C

ATOM 2608 OD1 ASP B 193 9.595 23.795 37.586 1.00 21.61 O

ATOM 2609 OD2 ASP B 193 8.943 25.701 38.420 1.00 27.86 O1-

ATOM 2610 H ASP B 193 8.262 21.948 42.020 1.00 0.00 H

ATOM 2611 HA ASP B 193 7.185 23.999 40.252 1.00 0.00 H

ATOM 2612 HB3 ASP B 193 10.147 23.198 39.999 1.00 0.00 H

ATOM 2613 HB2 ASP B 193 9.632 24.731 40.637 1.00 0.00 H

ATOM 2614 N ASP B 194 7.146 22.194 38.422 1.00 22.03 N

ATOM 2615 CA ASP B 194 6.808 21.047 37.582 1.00 21.53 C

ATOM 2616 C ASP B 194 8.041 20.269 37.130 1.00 22.92 C

ATOM 2617 O ASP B 194 7.939 19.100 36.761 1.00 22.56 O

ATOM 2618 CB ASP B 194 6.012 21.494 36.345 1.00 21.45 C

ATOM 2619 CG ASP B 194 4.586 21.886 36.675 1.00 29.47 C

ATOM 2620 OD1 ASP B 194 4.111 21.544 37.780 1.00 30.17 O

ATOM 2621 OD2 ASP B 194 3.936 22.526 35.817 1.00 34.55 O1-

ATOM 2622 H ASP B 194 6.918 23.120 38.090 1.00 0.00 H

ATOM 2623 HA ASP B 194 6.222 20.338 38.171 1.00 0.00 H

ATOM 2624 HB3 ASP B 194 5.952 20.688 35.612 1.00 0.00 H

ATOM 2625 HB2 ASP B 194 6.510 22.328 35.849 1.00 0.00 H

ATOM 2626 N ARG B 195 9.201 20.916 37.134 1.00 17.84 N

ATOM 2627 CA ARG B 195 10.418 20.248 36.671 1.00 16.73 C

ATOM 2628 C ARG B 195 10.916 19.199 37.662 1.00 18.50 C

ATOM 2629 O ARG B 195 11.636 18.261 37.293 1.00 16.09 O

ATOM 2630 CB ARG B 195 11.516 21.276 36.390 1.00 15.79 C

ATOM 2631 CG ARG B 195 12.279 21.745 37.629 1.00 16.25 C

ATOM 2632 CD ARG B 195 13.095 22.982 37.307 1.00 19.11 C

ATOM 2633 NE ARG B 195 12.231 24.139 37.073 1.00 20.79 N

ATOM 2634 CZ ARG B 195 12.666 25.322 36.670 1.00 23.76 C

ATOM 2635 NH1 ARG B 195 13.961 25.511 36.444 1.00 19.20 N

ATOM 2636 NH2 ARG B 195 11.798 26.317 36.489 1.00 23.19 N1+

ATOM 2637 H ARG B 195 9.244 21.892 37.407 1.00 0.00 H

ATOM 2638 HA ARG B 195 10.199 19.747 35.726 1.00 0.00 H

ATOM 2639 HB3 ARG B 195 11.076 22.117 35.853 1.00 0.00 H

ATOM 2640 HB2 ARG B 195 12.238 20.840 35.697 1.00 0.00 H

ATOM 2641 HG3 ARG B 195 12.939 20.968 38.015 1.00 0.00 H

ATOM 2642 HG2 ARG B 195 11.584 21.983 38.435 1.00 0.00 H

ATOM 2643 HD3 ARG B 195 13.727 22.798 36.439 1.00 0.00 H

ATOM 2644 HD2 ARG B 195 13.756 23.203 38.142 1.00 0.00 H

ATOM 2645 HE ARG B 195 11.232 24.008 37.237 1.00 0.00 H

ATOM 2646 HH12 ARG B 195 14.299 26.441 36.223 1.00 0.00 H

ATOM 2647 HH11 ARG B 195 14.636 24.755 36.525 1.00 0.00 H

ATOM 2648 HH22 ARG B 195 12.121 27.253 36.251 1.00 0.00 H

ATOM 2649 HH21 ARG B 195 10.816 26.171 36.684 1.00 0.00 H

ATOM 2650 N PHE B 196 10.538 19.345 38.927 1.00 17.69 N

ATOM 2651 CA PHE B 196 10.962 18.390 39.935 1.00 15.29 C

ATOM 2652 C PHE B 196 9.984 17.231 39.992 1.00 22.10 C

ATOM 2653 O PHE B 196 8.770 17.428 39.918 1.00 23.70 O

ATOM 2654 CB PHE B 196 11.103 19.061 41.303 1.00 19.22 C

ATOM 2655 CG PHE B 196 12.181 20.107 41.349 1.00 15.52 C

ATOM 2656 CD1 PHE B 196 13.522 19.749 41.252 1.00 14.06 C

ATOM 2657 CD2 PHE B 196 11.857 21.443 41.506 1.00 16.84 C

ATOM 2658 CE1 PHE B 196 14.517 20.713 41.292 1.00 13.65 C

ATOM 2659 CE2 PHE B 196 12.847 22.412 41.546 1.00 18.57 C

ATOM 2660 CZ PHE B 196 14.182 22.040 41.430 1.00 16.00 C

ATOM 2661 H PHE B 196 9.916 20.097 39.192 1.00 0.00 H

ATOM 2662 HA PHE B 196 11.953 18.063 39.687 1.00 0.00 H

ATOM 2663 HB3 PHE B 196 11.347 18.314 42.059 1.00 0.00 H

ATOM 2664 HB2 PHE B 196 10.150 19.496 41.611 1.00 0.00 H

ATOM 2665 HD1 PHE B 196 13.798 18.710 41.142 1.00 0.00 H

ATOM 2666 HD2 PHE B 196 10.823 21.738 41.604 1.00 0.00 H

ATOM 2667 HE1 PHE B 196 15.555 20.426 41.216 1.00 0.00 H

ATOM 2668 HE2 PHE B 196 12.581 23.452 41.666 1.00 0.00 H

ATOM 2669 HZ PHE B 196 14.957 22.791 41.460 1.00 0.00 H

ATOM 2670 N PHE B 197 10.510 16.018 40.106 1.00 15.85 N

ATOM 2671 CA PHE B 197 9.659 14.835 40.052 1.00 16.05 C

ATOM 2672 C PHE B 197 10.167 13.739 40.978 1.00 14.81 C

ATOM 2673 O PHE B 197 11.340 13.373 40.941 1.00 16.78 O

ATOM 2674 CB PHE B 197 9.546 14.317 38.607 1.00 14.53 C

ATOM 2675 CG PHE B 197 8.726 13.062 38.488 1.00 17.65 C

ATOM 2676 CD1 PHE B 197 7.382 13.067 38.832 1.00 22.06 C

ATOM 2677 CD2 PHE B 197 9.300 11.873 38.073 1.00 16.60 C

ATOM 2678 CE1 PHE B 197 6.630 11.908 38.752 1.00 21.51 C

ATOM 2679 CE2 PHE B 197 8.551 10.721 37.989 1.00 20.51 C

ATOM 2680 CZ PHE B 197 7.216 10.738 38.326 1.00 21.21 C

ATOM 2681 H PHE B 197 11.515 15.901 40.135 1.00 0.00 H

ATOM 2682 HA PHE B 197 8.654 15.079 40.403 1.00 0.00 H

ATOM 2683 HB3 PHE B 197 10.541 14.146 38.192 1.00 0.00 H

ATOM 2684 HB2 PHE B 197 9.090 15.081 37.977 1.00 0.00 H

ATOM 2685 HD1 PHE B 197 6.912 13.980 39.167 1.00 0.00 H

ATOM 2686 HD2 PHE B 197 10.346 11.853 37.811 1.00 0.00 H

ATOM 2687 HE1 PHE B 197 5.586 11.920 39.028 1.00 0.00 H

ATOM 2688 HE2 PHE B 197 9.013 9.800 37.665 1.00 0.00 H

ATOM 2689 HZ PHE B 197 6.635 9.830 38.290 1.00 0.00 H

ATOM 2690 N ARG B 198 9.283 13.220 41.825 1.00 14.50 N

ATOM 2691 CA ARG B 198 9.629 12.117 42.717 1.00 16.51 C

ATOM 2692 C ARG B 198 9.562 10.807 41.934 1.00 19.79 C

ATOM 2693 O ARG B 198 8.479 10.267 41.724 1.00 20.71 O

ATOM 2694 CB ARG B 198 8.652 12.077 43.894 1.00 18.10 C

ATOM 2695 CG ARG B 198 8.954 11.011 44.958 1.00 20.56 C

ATOM 2696 CD ARG B 198 10.104 11.410 45.857 1.00 25.39 C

ATOM 2697 NE ARG B 198 10.249 10.535 47.023 1.00 19.81 N

ATOM 2698 CZ ARG B 198 9.622 10.707 48.186 1.00 22.92 C

ATOM 2699 NH1 ARG B 198 8.782 11.722 48.363 1.00 21.81 N

ATOM 2700 NH2 ARG B 198 9.839 9.855 49.181 1.00 23.93 N1+

ATOM 2701 H ARG B 198 8.329 13.549 41.817 1.00 0.00 H

ATOM 2702 HA ARG B 198 10.633 12.258 43.115 1.00 0.00 H

ATOM 2703 HB3 ARG B 198 7.638 11.920 43.522 1.00 0.00 H

ATOM 2704 HB2 ARG B 198 8.622 13.058 44.371 1.00 0.00 H

ATOM 2705 HG3 ARG B 198 9.140 10.032 44.515 1.00 0.00 H

ATOM 2706 HG2 ARG B 198 8.063 10.886 45.574 1.00 0.00 H

ATOM 2707 HD3 ARG B 198 9.994 12.440 46.195 1.00 0.00 H

ATOM 2708 HD2 ARG B 198 11.036 11.375 45.304 1.00 0.00 H

ATOM 2709 HE ARG B 198 10.862 9.739 46.901 1.00 0.00 H

ATOM 2710 HH12 ARG B 198 8.305 11.847 49.244 1.00 0.00 H

ATOM 2711 HH11 ARG B 198 8.615 12.373 47.610 1.00 0.00 H

ATOM 2712 HH22 ARG B 198 9.364 9.958 50.066 1.00 0.00 H

ATOM 2713 HH21 ARG B 198 10.524 9.116 49.076 1.00 0.00 H

ATOM 2714 N CYS B 199 10.712 10.307 41.494 1.00 16.80 N

ATOM 2715 CA CYS B 199 10.742 9.133 40.622 1.00 14.86 C

ATOM 2716 C CYS B 199 11.068 7.847 41.384 1.00 16.67 C

ATOM 2717 O CYS B 199 11.120 6.765 40.794 1.00 18.39 O

ATOM 2718 CB CYS B 199 11.732 9.339 39.464 1.00 14.02 C

ATOM 2719 SG CYS B 199 13.465 9.405 39.949 1.00 18.17 S

ATOM 2720 H CYS B 199 11.580 10.786 41.693 1.00 0.00 H

ATOM 2721 HA CYS B 199 9.772 8.982 40.153 1.00 0.00 H

ATOM 2722 HB3 CYS B 199 11.503 10.265 38.940 1.00 0.00 H

ATOM 2723 HB2 CYS B 199 11.620 8.541 38.728 1.00 0.00 H

ATOM 2724 HG CYS B 199 13.528 8.168 40.452 1.00 0.00 H

ATOM 2725 N HIS B 200 11.257 7.967 42.694 1.00 16.97 N

ATOM 2726 CA HIS B 200 11.753 6.876 43.513 1.00 16.74 C

ATOM 2727 C HIS B 200 11.583 7.320 44.951 1.00 21.26 C

ATOM 2728 O HIS B 200 11.490 8.514 45.237 1.00 16.95 O

ATOM 2729 CB HIS B 200 13.235 6.659 43.197 1.00 17.39 C

ATOM 2730 CG HIS B 200 13.845 5.449 43.835 1.00 18.83 C

ATOM 2731 ND1 HIS B 200 14.303 5.446 45.132 1.00 21.51 N

ATOM 2732 CD2 HIS B 200 14.136 4.226 43.330 1.00 21.04 C

ATOM 2733 CE1 HIS B 200 14.820 4.263 45.413 1.00 23.17 C

ATOM 2734 NE2 HIS B 200 14.730 3.503 44.337 1.00 23.48 N

ATOM 2735 H HIS B 200 11.176 8.877 43.126 1.00 0.00 H

ATOM 2736 HA HIS B 200 11.171 5.971 43.330 1.00 0.00 H

ATOM 2737 HB3 HIS B 200 13.796 7.542 43.497 1.00 0.00 H

ATOM 2738 HB2 HIS B 200 13.390 6.555 42.129 1.00 0.00 H

ATOM 2739 HD2 HIS B 200 13.979 3.812 42.346 1.00 0.00 H

ATOM 2740 HE1 HIS B 200 15.257 3.975 46.358 1.00 0.00 H

ATOM 2741 HE2 HIS B 200 15.062 2.552 44.264 1.00 0.00 H

ATOM 2742 N ASN B 201 11.540 6.372 45.874 1.00 21.81 N

ATOM 2743 CA ASN B 201 11.421 6.752 47.272 1.00 22.09 C

ATOM 2744 C ASN B 201 12.591 7.627 47.740 1.00 21.74 C

ATOM 2745 O ASN B 201 12.416 8.494 48.594 1.00 20.37 O

ATOM 2746 CB ASN B 201 11.295 5.514 48.154 1.00 25.44 C

ATOM 2747 CG ASN B 201 11.197 5.861 49.626 1.00 35.38 C

ATOM 2748 OD1 ASN B 201 10.211 6.448 50.072 1.00 38.35 O

ATOM 2749 ND2 ASN B 201 12.227 5.509 50.385 1.00 31.06 N

ATOM 2750 H ASN B 201 11.636 5.398 45.626 1.00 0.00 H

ATOM 2751 HA ASN B 201 10.504 7.336 47.384 1.00 0.00 H

ATOM 2752 HB3 ASN B 201 12.140 4.843 47.984 1.00 0.00 H

ATOM 2753 HB2 ASN B 201 10.401 4.953 47.878 1.00 0.00 H

ATOM 2754 HD22 ASN B 201 12.214 5.725 51.371 1.00 0.00 H

ATOM 2755 HD21 ASN B 201 13.020 5.033 49.980 1.00 0.00 H

ATOM 2756 N SER B 202 13.768 7.418 47.151 1.00 17.91 N

ATOM 2757 CA SER B 202 14.976 8.125 47.566 1.00 18.62 C

ATOM 2758 C SER B 202 15.333 9.353 46.737 1.00 17.95 C

ATOM 2759 O SER B 202 16.270 10.064 47.086 1.00 16.26 O

ATOM 2760 CB SER B 202 16.180 7.183 47.527 1.00 20.81 C

ATOM 2761 OG SER B 202 16.016 6.105 48.425 1.00 22.80 O

ATOM 2762 H SER B 202 13.858 6.695 46.447 1.00 0.00 H

ATOM 2763 HA SER B 202 14.868 8.471 48.592 1.00 0.00 H

ATOM 2764 HB3 SER B 202 17.101 7.698 47.805 1.00 0.00 H

ATOM 2765 HB2 SER B 202 16.334 6.786 46.524 1.00 0.00 H

ATOM 2766 HG SER B 202 15.975 6.453 49.303 1.00 0.00 H

ATOM 2767 N PHE B 203 14.635 9.587 45.627 1.00 17.18 N

ATOM 2768 CA PHE B 203 15.095 10.602 44.680 1.00 14.02 C

ATOM 2769 C PHE B 203 14.007 11.532 44.157 1.00 15.61 C

ATOM 2770 O PHE B 203 12.922 11.086 43.782 1.00 15.86 O

ATOM 2771 CB PHE B 203 15.749 9.939 43.464 1.00 13.23 C

ATOM 2772 CG PHE B 203 16.887 9.018 43.803 1.00 14.87 C

ATOM 2773 CD1 PHE B 203 18.086 9.523 44.288 1.00 15.83 C

ATOM 2774 CD2 PHE B 203 16.774 7.648 43.595 1.00 17.21 C

ATOM 2775 CE1 PHE B 203 19.141 8.677 44.588 1.00 17.53 C

ATOM 2776 CE2 PHE B 203 17.828 6.799 43.893 1.00 16.17 C

ATOM 2777 CZ PHE B 203 19.008 7.311 44.388 1.00 16.86 C

ATOM 2778 H PHE B 203 13.853 8.998 45.376 1.00 0.00 H

ATOM 2779 HA PHE B 203 15.844 11.236 45.131 1.00 0.00 H

ATOM 2780 HB3 PHE B 203 16.140 10.703 42.789 1.00 0.00 H

ATOM 2781 HB2 PHE B 203 15.007 9.391 42.882 1.00 0.00 H

ATOM 2782 HD1 PHE B 203 18.199 10.585 44.445 1.00 0.00 H

ATOM 2783 HD2 PHE B 203 15.876 7.227 43.182 1.00 0.00 H

ATOM 2784 HE1 PHE B 203 20.072 9.079 44.956 1.00 0.00 H

ATOM 2785 HE2 PHE B 203 17.725 5.737 43.729 1.00 0.00 H

ATOM 2786 HZ PHE B 203 19.832 6.651 44.616 1.00 0.00 H

ATOM 2787 N VAL B 204 14.322 12.825 44.105 1.00 13.45 N

ATOM 2788 CA VAL B 204 13.509 13.806 43.400 1.00 13.11 C

ATOM 2789 C VAL B 204 14.411 14.388 42.329 1.00 13.30 C

ATOM 2790 O VAL B 204 15.438 14.983 42.625 1.00 12.41 O

ATOM 2791 CB VAL B 204 13.011 14.921 44.339 1.00 13.39 C

ATOM 2792 CG1 VAL B 204 12.442 16.092 43.549 1.00 12.76 C

ATOM 2793 CG2 VAL B 204 11.966 14.368 45.295 1.00 14.68 C

ATOM 2794 H VAL B 204 15.231 13.120 44.439 1.00 0.00 H

ATOM 2795 HA VAL B 204 12.648 13.343 42.932 1.00 0.00 H

ATOM 2796 HB VAL B 204 13.843 15.291 44.934 1.00 0.00 H

ATOM 2797 HG11 VAL B 204 11.979 16.813 44.223 1.00 0.00 H

ATOM 2798 HG12 VAL B 204 13.196 16.649 42.992 1.00 0.00 H

ATOM 2799 HG13 VAL B 204 11.673 15.767 42.848 1.00 0.00 H

ATOM 2800 HG21 VAL B 204 11.703 15.115 46.040 1.00 0.00 H

ATOM 2801 HG22 VAL B 204 11.058 14.087 44.763 1.00 0.00 H

ATOM 2802 HG23 VAL B 204 12.325 13.486 45.822 1.00 0.00 H

ATOM 2803 N VAL B 205 14.047 14.195 41.065 1.00 12.15 N

ATOM 2804 CA VAL B 205 14.926 14.628 39.985 1.00 11.92 C

ATOM 2805 C VAL B 205 14.509 15.968 39.394 1.00 13.13 C

ATOM 2806 O VAL B 205 13.325 16.334 39.418 1.00 15.39 O

ATOM 2807 CB VAL B 205 14.993 13.572 38.857 1.00 14.75 C

ATOM 2808 CG1 VAL B 205 15.547 12.271 39.398 1.00 17.09 C

ATOM 2809 CG2 VAL B 205 13.610 13.344 38.248 1.00 14.58 C

ATOM 2810 H VAL B 205 13.179 13.724 40.843 1.00 0.00 H

ATOM 2811 HA VAL B 205 15.952 14.752 40.327 1.00 0.00 H

ATOM 2812 HB VAL B 205 15.665 13.923 38.072 1.00 0.00 H

ATOM 2813 HG11 VAL B 205 15.731 11.547 38.604 1.00 0.00 H

ATOM 2814 HG12 VAL B 205 16.465 12.426 39.963 1.00 0.00 H

ATOM 2815 HG13 VAL B 205 14.850 11.818 40.100 1.00 0.00 H

ATOM 2816 HG21 VAL B 205 13.660 12.568 37.484 1.00 0.00 H

ATOM 2817 HG22 VAL B 205 12.884 12.999 38.980 1.00 0.00 H

ATOM 2818 HG23 VAL B 205 13.206 14.230 37.757 1.00 0.00 H

ATOM 2819 N ASN B 206 15.484 16.713 38.882 1.00 11.58 N

ATOM 2820 CA ASN B 206 15.181 17.884 38.076 1.00 10.40 C

ATOM 2821 C ASN B 206 15.126 17.469 36.605 1.00 11.17 C

ATOM 2822 O ASN B 206 16.156 17.183 35.990 1.00 12.27 O

ATOM 2823 CB ASN B 206 16.241 18.973 38.283 1.00 12.79 C

ATOM 2824 CG ASN B 206 15.911 20.266 37.551 1.00 13.87 C

ATOM 2825 OD1 ASN B 206 15.083 20.289 36.631 1.00 13.86 O

ATOM 2826 ND2 ASN B 206 16.564 21.353 37.952 1.00 13.63 N

ATOM 2827 H ASN B 206 16.445 16.397 38.920 1.00 0.00 H

ATOM 2828 HA ASN B 206 14.222 18.321 38.365 1.00 0.00 H

ATOM 2829 HB3 ASN B 206 17.218 18.626 37.946 1.00 0.00 H

ATOM 2830 HB2 ASN B 206 16.342 19.186 39.348 1.00 0.00 H

ATOM 2831 HD22 ASN B 206 16.399 22.237 37.481 1.00 0.00 H

ATOM 2832 HD21 ASN B 206 17.236 21.301 38.705 1.00 0.00 H

ATOM 2833 N ARG B 207 13.917 17.431 36.050 1.00 11.49 N

ATOM 2834 CA ARG B 207 13.744 17.061 34.642 1.00 11.46 C

ATOM 2835 C ARG B 207 14.621 17.856 33.681 1.00 14.00 C

ATOM 2836 O ARG B 207 15.069 17.316 32.660 1.00 14.66 O

ATOM 2837 CB ARG B 207 12.267 17.190 34.232 1.00 15.00 C

ATOM 2838 CG ARG B 207 11.377 16.067 34.755 1.00 15.45 C

ATOM 2839 CD ARG B 207 9.906 16.470 34.709 1.00 17.88 C

ATOM 2840 NE ARG B 207 9.017 15.317 34.830 1.00 17.64 N

ATOM 2841 CZ ARG B 207 7.864 15.325 35.497 1.00 20.03 C

ATOM 2842 NH1 ARG B 207 7.470 16.419 36.146 1.00 22.03 N

ATOM 2843 NH2 ARG B 207 7.118 14.228 35.537 1.00 24.00 N1+

ATOM 2844 H ARG B 207 13.098 17.680 36.594 1.00 0.00 H

ATOM 2845 HA ARG B 207 14.038 16.018 34.540 1.00 0.00 H

ATOM 2846 HB3 ARG B 207 12.173 17.207 33.145 1.00 0.00 H

ATOM 2847 HB2 ARG B 207 11.892 18.157 34.571 1.00 0.00 H

ATOM 2848 HG3 ARG B 207 11.638 15.834 35.788 1.00 0.00 H

ATOM 2849 HG2 ARG B 207 11.553 15.156 34.181 1.00 0.00 H

ATOM 2850 HD3 ARG B 207 9.677 16.951 33.758 1.00 0.00 H

ATOM 2851 HD2 ARG B 207 9.729 17.213 35.485 1.00 0.00 H

ATOM 2852 HE ARG B 207 9.240 14.508 34.256 1.00 0.00 H

ATOM 2853 HH12 ARG B 207 6.598 16.426 36.655 1.00 0.00 H

ATOM 2854 HH11 ARG B 207 8.025 17.267 36.138 1.00 0.00 H

ATOM 2855 HH22 ARG B 207 6.265 14.210 36.077 1.00 0.00 H

ATOM 2856 HH21 ARG B 207 7.384 13.402 35.016 1.00 0.00 H

ATOM 2857 N HIS B 208 14.871 19.132 33.984 1.00 11.88 N

ATOM 2858 CA HIS B 208 15.670 19.966 33.081 1.00 12.90 C

ATOM 2859 C HIS B 208 17.095 19.441 32.938 1.00 16.02 C

ATOM 2860 O HIS B 208 17.761 19.703 31.936 1.00 17.52 O

ATOM 2861 CB HIS B 208 15.726 21.415 33.562 1.00 14.97 C

ATOM 2862 CG HIS B 208 14.420 22.137 33.449 1.00 17.33 C

ATOM 2863 ND1 HIS B 208 14.276 23.469 33.774 1.00 16.49 N

ATOM 2864 CD2 HIS B 208 13.198 21.709 33.050 1.00 18.14 C

ATOM 2865 CE1 HIS B 208 13.020 23.831 33.583 1.00 18.48 C

ATOM 2866 NE2 HIS B 208 12.342 22.783 33.151 1.00 18.36 N

ATOM 2867 H HIS B 208 14.525 19.535 34.845 1.00 0.00 H

ATOM 2868 HA HIS B 208 15.219 19.943 32.086 1.00 0.00 H

ATOM 2869 HB3 HIS B 208 16.443 21.980 32.964 1.00 0.00 H

ATOM 2870 HB2 HIS B 208 16.083 21.476 34.590 1.00 0.00 H

ATOM 2871 HD1 HIS B 208 15.025 24.072 34.105 1.00 0.00 H

ATOM 2872 HD2 HIS B 208 12.864 20.738 32.713 1.00 0.00 H

ATOM 2873 HE1 HIS B 208 12.614 24.816 33.762 1.00 0.00 H

ATOM 2874 N ASN B 209 17.573 18.713 33.948 1.00 12.39 N

ATOM 2875 CA ASN B 209 18.961 18.268 33.960 1.00 11.76 C

ATOM 2876 C ASN B 209 19.148 16.782 33.679 1.00 12.83 C

ATOM 2877 O ASN B 209 20.247 16.256 33.776 1.00 14.36 O

ATOM 2878 CB ASN B 209 19.621 18.660 35.288 1.00 12.33 C

ATOM 2879 CG ASN B 209 19.962 20.128 35.321 1.00 14.28 C

ATOM 2880 OD1 ASN B 209 20.402 20.683 34.306 1.00 16.86 O

ATOM 2881 ND2 ASN B 209 19.755 20.778 36.472 1.00 15.16 N

ATOM 2882 H ASN B 209 16.983 18.492 34.740 1.00 0.00 H

ATOM 2883 HA ASN B 209 19.538 18.712 33.151 1.00 0.00 H

ATOM 2884 HB3 ASN B 209 20.557 18.125 35.439 1.00 0.00 H

ATOM 2885 HB2 ASN B 209 18.976 18.394 36.128 1.00 0.00 H

ATOM 2886 HD22 ASN B 209 20.008 21.756 36.539 1.00 0.00 H

ATOM 2887 HD21 ASN B 209 19.402 20.292 37.287 1.00 0.00 H

ATOM 2888 N ILE B 210 18.062 16.115 33.321 1.00 12.76 N

ATOM 2889 CA ILE B 210 18.142 14.730 32.875 1.00 10.17 C

ATOM 2890 C ILE B 210 18.660 14.687 31.444 1.00 14.01 C

ATOM 2891 O ILE B 210 18.157 15.398 30.574 1.00 15.38 O

ATOM 2892 CB ILE B 210 16.778 14.060 32.947 1.00 12.37 C

ATOM 2893 CG1 ILE B 210 16.360 13.913 34.401 1.00 12.04 C

ATOM 2894 CG2 ILE B 210 16.809 12.694 32.292 1.00 13.20 C

ATOM 2895 CD1 ILE B 210 14.929 13.396 34.552 1.00 14.62 C

ATOM 2896 H ILE B 210 17.176 16.597 33.250 1.00 0.00 H

ATOM 2897 HA ILE B 210 18.823 14.175 33.523 1.00 0.00 H

ATOM 2898 HB ILE B 210 16.044 14.677 32.425 1.00 0.00 H

ATOM 2899 HG13 ILE B 210 16.408 14.880 34.897 1.00 0.00 H

ATOM 2900 HG12 ILE B 210 17.051 13.268 34.944 1.00 0.00 H

ATOM 2901 HG21 ILE B 210 15.867 12.162 32.413 1.00 0.00 H

ATOM 2902 HG22 ILE B 210 16.955 12.733 31.213 1.00 0.00 H

ATOM 2903 HG23 ILE B 210 17.588 12.060 32.716 1.00 0.00 H

ATOM 2904 HD11 ILE B 210 14.552 13.609 35.551 1.00 0.00 H

ATOM 2905 HD12 ILE B 210 14.247 13.855 33.836 1.00 0.00 H

ATOM 2906 HD13 ILE B 210 14.888 12.317 34.418 1.00 0.00 H

ATOM 2907 N GLU B 211 19.686 13.874 31.216 1.00 13.23 N

ATOM 2908 CA GLU B 211 20.222 13.703 29.865 1.00 16.88 C

ATOM 2909 C GLU B 211 19.461 12.621 29.102 1.00 13.99 C

ATOM 2910 O GLU B 211 19.052 12.815 27.944 1.00 15.48 O

ATOM 2911 CB GLU B 211 21.705 13.330 29.939 1.00 18.39 C

ATOM 2912 CG GLU B 211 22.332 13.083 28.572 1.00 21.14 C

ATOM 2913 CD GLU B 211 22.997 14.309 28.019 1.00 35.55 C

ATOM 2914 OE1 GLU B 211 22.438 15.414 28.178 1.00 41.51 O

ATOM 2915 OE2 GLU B 211 24.090 14.170 27.432 1.00 57.07 O1-

ATOM 2916 H GLU B 211 20.045 13.288 31.962 1.00 0.00 H

ATOM 2917 HA GLU B 211 20.144 14.636 29.303 1.00 0.00 H

ATOM 2918 HB3 GLU B 211 21.820 12.426 30.539 1.00 0.00 H

ATOM 2919 HB2 GLU B 211 22.251 14.098 30.489 1.00 0.00 H

ATOM 2920 HG3 GLU B 211 21.677 12.664 27.811 1.00 0.00 H

ATOM 2921 HG2 GLU B 211 23.115 12.335 28.704 1.00 0.00 H

ATOM 2922 N SER B 212 19.286 11.476 29.746 1.00 15.12 N

ATOM 2923 CA SER B 212 18.574 10.367 29.133 1.00 16.77 C

ATOM 2924 C SER B 212 18.062 9.428 30.201 1.00 16.17 C

ATOM 2925 O SER B 212 18.386 9.573 31.387 1.00 17.36 O

ATOM 2926 CB SER B 212 19.480 9.610 28.156 1.00 19.51 C

ATOM 2927 OG SER B 212 20.519 8.925 28.834 1.00 20.02 O

ATOM 2928 H SER B 212 19.629 11.363 30.690 1.00 0.00 H

ATOM 2929 HA SER B 212 17.701 10.747 28.598 1.00 0.00 H

ATOM 2930 HB3 SER B 212 19.923 10.286 27.424 1.00 0.00 H

ATOM 2931 HB2 SER B 212 18.904 8.881 27.585 1.00 0.00 H

ATOM 2932 HG SER B 212 21.069 8.501 28.193 1.00 0.00 H

ATOM 2933 N ILE B 213 17.255 8.460 29.784 1.00 16.38 N

ATOM 2934 CA ILE B 213 16.636 7.521 30.705 1.00 15.23 C

ATOM 2935 C ILE B 213 16.726 6.128 30.102 1.00 21.47 C

ATOM 2936 O ILE B 213 16.523 5.952 28.905 1.00 20.04 O

ATOM 2937 CB ILE B 213 15.153 7.858 30.949 1.00 18.45 C

ATOM 2938 CG1 ILE B 213 15.010 9.254 31.557 1.00 17.20 C

ATOM 2939 CG2 ILE B 213 14.511 6.848 31.883 1.00 22.77 C

ATOM 2940 CD1 ILE B 213 13.573 9.717 31.636 1.00 24.72 C

ATOM 2941 H ILE B 213 17.042 8.366 28.801 1.00 0.00 H

ATOM 2942 HA ILE B 213 17.158 7.502 31.664 1.00 0.00 H

ATOM 2943 HB ILE B 213 14.622 7.840 29.995 1.00 0.00 H

ATOM 2944 HG13 ILE B 213 15.521 9.995 30.946 1.00 0.00 H

ATOM 2945 HG12 ILE B 213 15.475 9.296 32.543 1.00 0.00 H

ATOM 2946 HG21 ILE B 213 13.478 7.093 32.120 1.00 0.00 H

ATOM 2947 HG22 ILE B 213 14.455 5.850 31.452 1.00 0.00 H

ATOM 2948 HG23 ILE B 213 15.053 6.773 32.827 1.00 0.00 H

ATOM 2949 HD11 ILE B 213 13.536 10.774 31.896 1.00 0.00 H

ATOM 2950 HD12 ILE B 213 13.064 9.604 30.678 1.00 0.00 H

ATOM 2951 HD13 ILE B 213 12.999 9.184 32.387 1.00 0.00 H

ATOM 2952 N ASP B 214 17.063 5.149 30.932 1.00 21.02 N

ATOM 2953 CA ASP B 214 16.971 3.746 30.538 1.00 21.62 C

ATOM 2954 C ASP B 214 15.815 3.164 31.327 1.00 21.39 C

ATOM 2955 O ASP B 214 15.952 2.873 32.513 1.00 21.00 O

ATOM 2956 CB ASP B 214 18.272 3.021 30.871 1.00 23.28 C

ATOM 2957 CG ASP B 214 18.265 1.564 30.437 1.00 30.91 C

ATOM 2958 OD1 ASP B 214 17.171 0.982 30.251 1.00 30.16 O

ATOM 2959 OD2 ASP B 214 19.367 1.002 30.276 1.00 38.82 O1-

ATOM 2960 H ASP B 214 17.266 5.360 31.902 1.00 0.00 H

ATOM 2961 HA ASP B 214 16.774 3.620 29.471 1.00 0.00 H

ATOM 2962 HB3 ASP B 214 18.499 3.070 31.937 1.00 0.00 H

ATOM 2963 HB2 ASP B 214 19.098 3.516 30.360 1.00 0.00 H

ATOM 2964 N SER B 215 14.666 2.997 30.681 1.00 20.78 N

ATOM 2965 CA SER B 215 13.479 2.573 31.409 1.00 21.13 C

ATOM 2966 C SER B 215 13.536 1.100 31.814 1.00 26.63 C

ATOM 2967 O SER B 215 12.951 0.698 32.818 1.00 27.76 O

ATOM 2968 CB SER B 215 12.208 2.866 30.609 1.00 33.12 C

ATOM 2969 OG SER B 215 12.121 2.023 29.479 1.00 37.99 O

ATOM 2970 H SER B 215 14.588 3.230 29.701 1.00 0.00 H

ATOM 2971 HA SER B 215 13.418 3.164 32.320 1.00 0.00 H

ATOM 2972 HB3 SER B 215 12.184 3.908 30.288 1.00 0.00 H

ATOM 2973 HB2 SER B 215 11.326 2.707 31.232 1.00 0.00 H

ATOM 2974 HG SER B 215 12.181 1.124 29.764 1.00 0.00 H

ATOM 2975 N LYS B 216 14.257 0.305 31.035 1.00 23.67 N

ATOM 2976 CA LYS B 216 14.403 -1.117 31.326 1.00 24.90 C

ATOM 2977 C LYS B 216 15.239 -1.333 32.585 1.00 23.99 C

ATOM 2978 O LYS B 216 14.867 -2.113 33.462 1.00 24.31 O

ATOM 2979 CB LYS B 216 15.045 -1.817 30.126 1.00 27.81 C

ATOM 2980 CG LYS B 216 15.312 -3.305 30.290 1.00 36.28 C

ATOM 2981 CD LYS B 216 15.874 -3.864 28.989 1.00 46.81 C

ATOM 2982 CE LYS B 216 16.375 -5.293 29.127 1.00 51.81 C

ATOM 2983 NZ LYS B 216 17.113 -5.720 27.895 1.00 42.84 N1+

ATOM 2984 H LYS B 216 14.781 0.697 30.263 1.00 0.00 H

ATOM 2985 HA LYS B 216 13.415 -1.553 31.488 1.00 0.00 H

ATOM 2986 HB3 LYS B 216 15.984 -1.321 29.876 1.00 0.00 H

ATOM 2987 HB2 LYS B 216 14.397 -1.670 29.261 1.00 0.00 H

ATOM 2988 HG3 LYS B 216 14.396 -3.831 30.563 1.00 0.00 H

ATOM 2989 HG2 LYS B 216 16.031 -3.477 31.093 1.00 0.00 H

ATOM 2990 HD3 LYS B 216 16.707 -3.236 28.668 1.00 0.00 H

ATOM 2991 HD2 LYS B 216 15.123 -3.806 28.200 1.00 0.00 H

ATOM 2992 HE3 LYS B 216 15.544 -5.975 29.309 1.00 0.00 H

ATOM 2993 HE2 LYS B 216 17.055 -5.378 29.976 1.00 0.00 H

ATOM 2994 HZ1 LYS B 216 17.903 -5.108 27.748 1.00 0.00 H

ATOM 2995 HZ2 LYS B 216 17.442 -6.668 28.011 1.00 0.00 H

ATOM 2996 HZ3 LYS B 216 16.497 -5.672 27.096 1.00 0.00 H

ATOM 2997 N GLU B 217 16.362 -0.623 32.670 1.00 22.55 N

ATOM 2998 CA GLU B 217 17.264 -0.725 33.818 1.00 21.48 C

ATOM 2999 C GLU B 217 16.860 0.226 34.931 1.00 19.55 C

ATOM 3000 O GLU B 217 17.408 0.172 36.033 1.00 21.00 O

ATOM 3001 CB GLU B 217 18.697 -0.414 33.389 1.00 30.43 C

ATOM 3002 CG GLU B 217 19.312 -1.463 32.488 1.00 39.40 C

ATOM 3003 CD GLU B 217 19.642 -2.730 33.239 1.00 31.97 C

ATOM 3004 OE1 GLU B 217 20.677 -2.753 33.945 1.00 42.85 O

ATOM 3005 OE2 GLU B 217 18.868 -3.704 33.128 1.00 48.67 O1-

ATOM 3006 H GLU B 217 16.628 -0.016 31.902 1.00 0.00 H

ATOM 3007 HA GLU B 217 17.232 -1.730 34.241 1.00 0.00 H

ATOM 3008 HB3 GLU B 217 19.333 -0.320 34.270 1.00 0.00 H

ATOM 3009 HB2 GLU B 217 18.741 0.565 32.916 1.00 0.00 H

ATOM 3010 HG3 GLU B 217 20.239 -1.075 32.065 1.00 0.00 H

ATOM 3011 HG2 GLU B 217 18.663 -1.685 31.640 1.00 0.00 H

ATOM 3012 N ARG B 218 15.908 1.101 34.626 1.00 19.04 N

ATOM 3013 CA ARG B 218 15.415 2.092 35.578 1.00 23.13 C

ATOM 3014 C ARG B 218 16.545 2.972 36.111 1.00 19.96 C

ATOM 3015 O ARG B 218 16.703 3.148 37.327 1.00 20.34 O

ATOM 3016 CB ARG B 218 14.649 1.412 36.711 1.00 25.44 C

ATOM 3017 CG ARG B 218 13.503 0.533 36.222 1.00 26.35 C

ATOM 3018 CD ARG B 218 12.585 0.102 37.358 1.00 29.09 C

ATOM 3019 NE ARG B 218 13.316 -0.414 38.516 1.00 35.22 N

ATOM 3020 CZ ARG B 218 13.846 -1.630 38.591 1.00 36.64 C

ATOM 3021 NH1 ARG B 218 13.739 -2.465 37.565 1.00 42.98 N

ATOM 3022 NH2 ARG B 218 14.493 -2.009 39.690 1.00 32.65 N1+

ATOM 3023 H ARG B 218 15.534 1.111 33.687 1.00 0.00 H

ATOM 3024 HA ARG B 218 14.734 2.742 35.032 1.00 0.00 H

ATOM 3025 HB3 ARG B 218 14.226 2.227 37.276 1.00 0.00 H

ATOM 3026 HB2 ARG B 218 15.312 0.859 37.378 1.00 0.00 H

ATOM 3027 HG3 ARG B 218 13.886 -0.343 35.699 1.00 0.00 H

ATOM 3028 HG2 ARG B 218 12.911 1.088 35.494 1.00 0.00 H

ATOM 3029 HD3 ARG B 218 11.862 -0.625 36.997 1.00 0.00 H

ATOM 3030 HD2 ARG B 218 12.017 0.969 37.687 1.00 0.00 H

ATOM 3031 HE ARG B 218 13.472 0.243 39.268 1.00 0.00 H

ATOM 3032 HH12 ARG B 218 13.269 -2.171 36.721 1.00 0.00 H

ATOM 3033 HH11 ARG B 218 14.161 -3.382 37.601 1.00 0.00 H

ATOM 3034 HH22 ARG B 218 14.924 -2.921 39.741 1.00 0.00 H

ATOM 3035 HH21 ARG B 218 14.580 -1.379 40.475 1.00 0.00 H

ATOM 3036 N ILE B 219 17.331 3.512 35.188 1.00 19.23 N

ATOM 3037 CA ILE B 219 18.434 4.408 35.511 1.00 17.26 C

ATOM 3038 C ILE B 219 18.182 5.745 34.825 1.00 18.67 C

ATOM 3039 O ILE B 219 17.869 5.796 33.634 1.00 20.18 O

ATOM 3040 CB ILE B 219 19.791 3.849 35.026 1.00 20.72 C

ATOM 3041 CG1 ILE B 219 20.050 2.476 35.653 1.00 22.30 C

ATOM 3042 CG2 ILE B 219 20.930 4.803 35.371 1.00 19.64 C

ATOM 3043 CD1 ILE B 219 21.242 1.739 35.047 1.00 27.72 C

ATOM 3044 H ILE B 219 17.140 3.326 34.212 1.00 0.00 H

ATOM 3045 HA ILE B 219 18.506 4.578 36.586 1.00 0.00 H

ATOM 3046 HB ILE B 219 19.759 3.726 33.942 1.00 0.00 H

ATOM 3047 HG13 ILE B 219 19.194 1.830 35.503 1.00 0.00 H

ATOM 3048 HG12 ILE B 219 20.177 2.565 36.731 1.00 0.00 H

ATOM 3049 HG21 ILE B 219 21.903 4.400 35.097 1.00 0.00 H

ATOM 3050 HG22 ILE B 219 20.855 5.757 34.849 1.00 0.00 H

ATOM 3051 HG23 ILE B 219 20.956 5.011 36.441 1.00 0.00 H

ATOM 3052 HD11 ILE B 219 21.298 0.722 35.436 1.00 0.00 H

ATOM 3053 HD12 ILE B 219 21.155 1.670 33.962 1.00 0.00 H

ATOM 3054 HD13 ILE B 219 22.190 2.220 35.282 1.00 0.00 H

ATOM 3055 N VAL B 220 18.286 6.824 35.592 1.00 14.38 N

ATOM 3056 CA VAL B 220 18.143 8.171 35.072 1.00 14.21 C

ATOM 3057 C VAL B 220 19.536 8.775 35.009 1.00 14.19 C

ATOM 3058 O VAL B 220 20.247 8.779 36.016 1.00 14.25 O

ATOM 3059 CB VAL B 220 17.263 9.022 36.019 1.00 11.48 C

ATOM 3060 CG1 VAL B 220 17.040 10.407 35.439 1.00 13.93 C

ATOM 3061 CG2 VAL B 220 15.937 8.326 36.277 1.00 14.83 C

ATOM 3062 H VAL B 220 18.548 6.717 36.565 1.00 0.00 H

ATOM 3063 HA VAL B 220 17.693 8.183 34.077 1.00 0.00 H

ATOM 3064 HB VAL B 220 17.761 9.136 36.983 1.00 0.00 H

ATOM 3065 HG11 VAL B 220 16.380 10.992 36.080 1.00 0.00 H

ATOM 3066 HG12 VAL B 220 17.965 10.976 35.347 1.00 0.00 H

ATOM 3067 HG13 VAL B 220 16.580 10.359 34.452 1.00 0.00 H

ATOM 3068 HG21 VAL B 220 15.279 8.952 36.881 1.00 0.00 H

ATOM 3069 HG22 VAL B 220 15.416 8.102 35.345 1.00 0.00 H

ATOM 3070 HG23 VAL B 220 16.061 7.391 36.825 1.00 0.00 H

ATOM 3071 N TYR B 221 19.933 9.274 33.843 1.00 13.27 N

ATOM 3072 CA TYR B 221 21.256 9.871 33.647 1.00 13.60 C

ATOM 3073 C TYR B 221 21.160 11.389 33.603 1.00 15.09 C

ATOM 3074 O TYR B 221 20.261 11.941 32.959 1.00 15.78 O

ATOM 3075 CB TYR B 221 21.893 9.373 32.343 1.00 15.13 C

ATOM 3076 CG TYR B 221 22.145 7.882 32.324 1.00 17.38 C

ATOM 3077 CD1 TYR B 221 21.251 7.014 31.720 1.00 18.50 C

ATOM 3078 CD2 TYR B 221 23.276 7.344 32.933 1.00 21.40 C

ATOM 3079 CE1 TYR B 221 21.479 5.633 31.717 1.00 23.05 C

ATOM 3080 CE2 TYR B 221 23.508 5.984 32.934 1.00 22.28 C

ATOM 3081 CZ TYR B 221 22.611 5.135 32.324 1.00 25.97 C

ATOM 3082 OH TYR B 221 22.851 3.778 32.332 1.00 27.03 O

ATOM 3083 H TYR B 221 19.303 9.258 33.050 1.00 0.00 H

ATOM 3084 HA TYR B 221 21.934 9.595 34.458 1.00 0.00 H

ATOM 3085 HB3 TYR B 221 22.847 9.877 32.176 1.00 0.00 H

ATOM 3086 HB2 TYR B 221 21.266 9.638 31.490 1.00 0.00 H

ATOM 3087 HD1 TYR B 221 20.358 7.401 31.250 1.00 0.00 H

ATOM 3088 HD2 TYR B 221 23.983 7.999 33.421 1.00 0.00 H

ATOM 3089 HE1 TYR B 221 20.769 4.970 31.245 1.00 0.00 H

ATOM 3090 HE2 TYR B 221 24.390 5.588 33.416 1.00 0.00 H

ATOM 3091 HH TYR B 221 22.180 3.277 31.894 1.00 0.00 H

ATOM 3092 N PHE B 222 22.095 12.065 34.270 1.00 14.82 N

ATOM 3093 CA PHE B 222 22.090 13.526 34.315 1.00 12.44 C

ATOM 3094 C PHE B 222 23.171 14.173 33.467 1.00 14.11 C

ATOM 3095 O PHE B 222 24.130 13.512 33.032 1.00 16.09 O

ATOM 3096 CB PHE B 222 22.154 13.991 35.770 1.00 12.81 C

ATOM 3097 CG PHE B 222 20.963 13.562 36.547 1.00 12.65 C

ATOM 3098 CD1 PHE B 222 19.861 14.386 36.622 1.00 11.45 C

ATOM 3099 CD2 PHE B 222 20.906 12.295 37.129 1.00 12.50 C

ATOM 3100 CE1 PHE B 222 18.731 13.974 37.297 1.00 11.51 C

ATOM 3101 CE2 PHE B 222 19.791 11.885 37.810 1.00 12.97 C

ATOM 3102 CZ PHE B 222 18.694 12.720 37.890 1.00 13.41 C

ATOM 3103 H PHE B 222 22.813 11.569 34.786 1.00 0.00 H

ATOM 3104 HA PHE B 222 21.153 13.915 33.922 1.00 0.00 H

ATOM 3105 HB3 PHE B 222 22.234 15.078 35.830 1.00 0.00 H

ATOM 3106 HB2 PHE B 222 23.052 13.601 36.252 1.00 0.00 H

ATOM 3107 HD1 PHE B 222 19.876 15.364 36.168 1.00 0.00 H

ATOM 3108 HD2 PHE B 222 21.753 11.639 37.067 1.00 0.00 H

ATOM 3109 HE1 PHE B 222 17.869 14.623 37.348 1.00 0.00 H

ATOM 3110 HE2 PHE B 222 19.768 10.906 38.261 1.00 0.00 H

ATOM 3111 HZ PHE B 222 17.818 12.376 38.392 1.00 0.00 H

ATOM 3112 N LYS B 223 23.006 15.467 33.220 1.00 14.65 N

ATOM 3113 CA LYS B 223 23.905 16.187 32.320 1.00 14.75 C

ATOM 3114 C LYS B 223 25.344 16.263 32.824 1.00 19.20 C

ATOM 3115 O LYS B 223 26.265 16.458 32.031 1.00 19.67 O

ATOM 3116 CB LYS B 223 23.344 17.575 32.009 1.00 18.17 C

ATOM 3117 CG LYS B 223 22.027 17.525 31.254 1.00 22.03 C

ATOM 3118 CD LYS B 223 21.514 18.919 30.912 1.00 26.85 C

ATOM 3119 CE LYS B 223 20.255 18.828 30.069 1.00 26.94 C

ATOM 3120 NZ LYS B 223 19.665 20.163 29.818 1.00 34.86 N1+

ATOM 3121 H LYS B 223 22.191 15.948 33.579 1.00 0.00 H

ATOM 3122 HA LYS B 223 23.945 15.635 31.378 1.00 0.00 H

ATOM 3123 HB3 LYS B 223 24.067 18.137 31.415 1.00 0.00 H

ATOM 3124 HB2 LYS B 223 23.212 18.134 32.938 1.00 0.00 H

ATOM 3125 HG3 LYS B 223 21.270 16.963 31.781 1.00 0.00 H

ATOM 3126 HG2 LYS B 223 22.188 16.977 30.325 1.00 0.00 H

ATOM 3127 HD3 LYS B 223 22.279 19.477 30.371 1.00 0.00 H

ATOM 3128 HD2 LYS B 223 21.315 19.472 31.831 1.00 0.00 H

ATOM 3129 HE3 LYS B 223 19.509 18.206 30.566 1.00 0.00 H

ATOM 3130 HE2 LYS B 223 20.474 18.353 29.112 1.00 0.00 H

ATOM 3131 HZ1 LYS B 223 20.329 20.748 29.332 1.00 0.00 H

ATOM 3132 HZ2 LYS B 223 18.829 20.059 29.261 1.00 0.00 H

ATOM 3133 HZ3 LYS B 223 19.417 20.584 30.704 1.00 0.00 H

ATOM 3134 N ASN B 224 25.536 16.078 34.132 1.00 15.44 N

ATOM 3135 CA ASN B 224 26.875 16.045 34.727 1.00 14.85 C

ATOM 3136 C ASN B 224 27.429 14.622 34.873 1.00 15.36 C

ATOM 3137 O ASN B 224 28.412 14.407 35.591 1.00 17.28 O

ATOM 3138 CB ASN B 224 26.855 16.725 36.102 1.00 14.68 C

ATOM 3139 CG ASN B 224 26.143 15.902 37.142 1.00 17.03 C

ATOM 3140 OD1 ASN B 224 25.364 15.001 36.823 1.00 14.90 O

ATOM 3141 ND2 ASN B 224 26.393 16.214 38.407 1.00 16.00 N

ATOM 3142 H ASN B 224 24.742 15.916 34.736 1.00 0.00 H

ATOM 3143 HA ASN B 224 27.581 16.605 34.111 1.00 0.00 H

ATOM 3144 HB3 ASN B 224 26.359 17.693 36.033 1.00 0.00 H

ATOM 3145 HB2 ASN B 224 27.875 16.925 36.434 1.00 0.00 H

ATOM 3146 HD22 ASN B 224 25.956 15.690 39.160 1.00 0.00 H

ATOM 3147 HD21 ASN B 224 27.050 16.953 38.625 1.00 0.00 H

ATOM 3148 N LYS B 225 26.797 13.676 34.179 1.00 17.69 N

ATOM 3149 CA LYS B 225 27.175 12.252 34.177 1.00 18.21 C

ATOM 3150 C LYS B 225 26.818 11.462 35.454 1.00 17.13 C

ATOM 3151 O LYS B 225 27.065 10.252 35.527 1.00 17.46 O

ATOM 3152 CB LYS B 225 28.654 12.057 33.805 1.00 18.39 C

ATOM 3153 CG LYS B 225 29.024 12.594 32.427 1.00 25.08 C

ATOM 3154 CD LYS B 225 30.505 12.376 32.144 1.00 33.02 C

ATOM 3155 CE LYS B 225 30.884 12.851 30.752 1.00 40.85 C

ATOM 3156 NZ LYS B 225 30.209 12.056 29.688 1.00 45.22 N1+

ATOM 3157 H LYS B 225 25.988 13.939 33.632 1.00 0.00 H

ATOM 3158 HA LYS B 225 26.573 11.799 33.388 1.00 0.00 H

ATOM 3159 HB3 LYS B 225 28.867 10.987 33.794 1.00 0.00 H

ATOM 3160 HB2 LYS B 225 29.330 12.451 34.562 1.00 0.00 H

ATOM 3161 HG3 LYS B 225 28.800 13.659 32.361 1.00 0.00 H

ATOM 3162 HG2 LYS B 225 28.406 12.098 31.678 1.00 0.00 H

ATOM 3163 HD3 LYS B 225 30.761 11.321 32.256 1.00 0.00 H

ATOM 3164 HD2 LYS B 225 31.099 12.917 32.882 1.00 0.00 H

ATOM 3165 HE3 LYS B 225 31.962 12.766 30.613 1.00 0.00 H

ATOM 3166 HE2 LYS B 225 30.631 13.904 30.626 1.00 0.00 H

ATOM 3167 HZ1 LYS B 225 29.208 12.146 29.786 1.00 0.00 H

ATOM 3168 HZ2 LYS B 225 30.488 12.400 28.780 1.00 0.00 H

ATOM 3169 HZ3 LYS B 225 30.469 11.084 29.775 1.00 0.00 H

ATOM 3170 N GLU B 226 26.238 12.113 36.460 1.00 14.25 N

ATOM 3171 CA GLU B 226 25.662 11.347 37.556 1.00 12.59 C

ATOM 3172 C GLU B 226 24.429 10.566 37.112 1.00 13.63 C

ATOM 3173 O GLU B 226 23.865 10.813 36.050 1.00 14.29 O

ATOM 3174 CB GLU B 226 25.262 12.245 38.732 1.00 14.78 C

ATOM 3175 CG GLU B 226 26.426 12.831 39.500 1.00 15.72 C

ATOM 3176 CD GLU B 226 25.958 13.513 40.760 1.00 16.03 C

ATOM 3177 OE1 GLU B 226 25.460 14.653 40.662 1.00 16.92 O

ATOM 3178 OE2 GLU B 226 26.060 12.896 41.839 1.00 16.47 O1-

ATOM 3179 H GLU B 226 26.055 13.106 36.400 1.00 0.00 H

ATOM 3180 HA GLU B 226 26.397 10.628 37.925 1.00 0.00 H

ATOM 3181 HB3 GLU B 226 24.669 11.668 39.445 1.00 0.00 H

ATOM 3182 HB2 GLU B 226 24.602 13.042 38.386 1.00 0.00 H

ATOM 3183 HG3 GLU B 226 26.983 13.535 38.883 1.00 0.00 H

ATOM 3184 HG2 GLU B 226 27.128 12.042 39.773 1.00 0.00 H

ATOM 3185 N HIS B 227 24.001 9.628 37.945 1.00 15.26 N

ATOM 3186 CA HIS B 227 22.772 8.898 37.688 1.00 12.68 C

ATOM 3187 C HIS B 227 22.032 8.671 38.994 1.00 15.44 C

ATOM 3188 O HIS B 227 22.610 8.772 40.083 1.00 15.05 O

ATOM 3189 CB HIS B 227 23.077 7.546 37.035 1.00 12.76 C

ATOM 3190 CG HIS B 227 23.624 6.536 37.996 1.00 14.23 C

ATOM 3191 ND1 HIS B 227 24.944 6.523 38.390 1.00 18.74 N

ATOM 3192 CD2 HIS B 227 23.014 5.542 38.683 1.00 17.62 C

ATOM 3193 CE1 HIS B 227 25.123 5.560 39.277 1.00 17.38 C

ATOM 3194 NE2 HIS B 227 23.973 4.938 39.457 1.00 17.54 N

ATOM 3195 H HIS B 227 24.488 9.452 38.813 1.00 0.00 H

ATOM 3196 HA HIS B 227 22.140 9.505 37.056 1.00 0.00 H

ATOM 3197 HB3 HIS B 227 23.763 7.662 36.195 1.00 0.00 H

ATOM 3198 HB2 HIS B 227 22.162 7.132 36.615 1.00 0.00 H

ATOM 3199 HD2 HIS B 227 21.983 5.224 38.688 1.00 0.00 H

ATOM 3200 HE1 HIS B 227 26.057 5.312 39.759 1.00 0.00 H

ATOM 3201 HE2 HIS B 227 23.817 4.145 40.067 1.00 0.00 H

ATOM 3202 N CYS B 228 20.745 8.372 38.893 1.00 13.73 N

ATOM 3203 CA CYS B 228 20.027 7.824 40.035 1.00 15.64 C

ATOM 3204 C CYS B 228 19.071 6.777 39.497 1.00 16.06 C

ATOM 3205 O CYS B 228 19.129 6.433 38.311 1.00 15.75 O

ATOM 3206 CB CYS B 228 19.304 8.910 40.845 1.00 14.41 C

ATOM 3207 SG CYS B 228 17.869 9.661 40.031 1.00 15.03 S

ATOM 3208 H CYS B 228 20.313 8.319 37.979 1.00 0.00 H

ATOM 3209 HA CYS B 228 20.710 7.289 40.699 1.00 0.00 H

ATOM 3210 HB3 CYS B 228 20.002 9.705 41.107 1.00 0.00 H

ATOM 3211 HB2 CYS B 228 18.963 8.491 41.787 1.00 0.00 H

ATOM 3212 HG CYS B 228 17.155 8.535 39.932 1.00 0.00 H

ATOM 3213 N TYR B 229 18.210 6.255 40.357 1.00 15.12 N

ATOM 3214 CA TYR B 229 17.309 5.189 39.943 1.00 17.95 C

ATOM 3215 C TYR B 229 15.866 5.630 39.997 1.00 16.48 C

ATOM 3216 O TYR B 229 15.527 6.617 40.653 1.00 17.78 O

ATOM 3217 CB TYR B 229 17.533 3.940 40.800 1.00 19.14 C

ATOM 3218 CG TYR B 229 18.989 3.551 40.835 1.00 21.44 C

ATOM 3219 CD1 TYR B 229 19.653 3.199 39.672 1.00 20.75 C

ATOM 3220 CD2 TYR B 229 19.707 3.575 42.023 1.00 22.53 C

ATOM 3221 CE1 TYR B 229 20.996 2.859 39.685 1.00 18.27 C

ATOM 3222 CE2 TYR B 229 21.058 3.234 42.049 1.00 21.80 C

ATOM 3223 CZ TYR B 229 21.688 2.869 40.878 1.00 20.89 C

ATOM 3224 OH TYR B 229 23.027 2.538 40.880 1.00 19.72 O

ATOM 3225 H TYR B 229 18.184 6.572 41.316 1.00 0.00 H

ATOM 3226 HA TYR B 229 17.483 4.894 38.910 1.00 0.00 H

ATOM 3227 HB3 TYR B 229 16.959 3.099 40.409 1.00 0.00 H

ATOM 3228 HB2 TYR B 229 17.177 4.110 41.818 1.00 0.00 H

ATOM 3229 HD1 TYR B 229 19.118 3.186 38.734 1.00 0.00 H

ATOM 3230 HD2 TYR B 229 19.220 3.862 42.944 1.00 0.00 H

ATOM 3231 HE1 TYR B 229 21.495 2.586 38.767 1.00 0.00 H

ATOM 3232 HE2 TYR B 229 21.602 3.258 42.982 1.00 0.00 H

ATOM 3233 HH TYR B 229 23.383 2.451 41.751 1.00 0.00 H

ATOM 3234 N ALA B 230 15.015 4.903 39.280 1.00 18.65 N

ATOM 3235 CA ALA B 230 13.588 5.166 39.302 1.00 19.79 C

ATOM 3236 C ALA B 230 12.841 3.886 39.623 1.00 22.60 C

ATOM 3237 O ALA B 230 13.265 2.795 39.253 1.00 25.26 O

ATOM 3238 CB ALA B 230 13.125 5.731 37.963 1.00 20.88 C

ATOM 3239 H ALA B 230 15.352 4.115 38.743 1.00 0.00 H

ATOM 3240 HA ALA B 230 13.329 5.896 40.063 1.00 0.00 H

ATOM 3241 HB1 ALA B 230 12.048 5.902 37.955 1.00 0.00 H

ATOM 3242 HB2 ALA B 230 13.607 6.687 37.759 1.00 0.00 H

ATOM 3243 HB3 ALA B 230 13.369 5.057 37.141 1.00 0.00 H

ATOM 3244 N SER B 231 11.731 4.021 40.329 1.00 21.43 N

ATOM 3245 CA SER B 231 10.912 2.866 40.657 1.00 23.39 C

ATOM 3246 C SER B 231 10.220 2.368 39.404 1.00 26.38 C

ATOM 3247 O SER B 231 10.055 3.114 38.444 1.00 25.17 O

ATOM 3248 CB SER B 231 9.866 3.255 41.700 1.00 24.41 C

ATOM 3249 OG SER B 231 8.853 4.055 41.117 1.00 27.04 O

ATOM 3250 H SER B 231 11.422 4.945 40.605 1.00 0.00 H

ATOM 3251 HA SER B 231 11.545 2.079 41.073 1.00 0.00 H

ATOM 3252 HB3 SER B 231 10.322 3.789 42.534 1.00 0.00 H

ATOM 3253 HB2 SER B 231 9.397 2.363 42.116 1.00 0.00 H

ATOM 3254 HG SER B 231 9.251 4.855 40.806 1.00 0.00 H

ATOM 3255 N VAL B 232 9.806 1.106 39.420 1.00 25.57 N

ATOM 3256 CA VAL B 232 9.047 0.542 38.311 1.00 29.25 C

ATOM 3257 C VAL B 232 7.818 1.391 37.973 1.00 26.07 C

ATOM 3258 O VAL B 232 7.519 1.650 36.804 1.00 27.75 O

ATOM 3259 CB VAL B 232 8.607 -0.910 38.617 1.00 27.46 C

ATOM 3260 CG1 VAL B 232 7.538 -1.362 37.631 1.00 31.71 C

ATOM 3261 CG2 VAL B 232 9.811 -1.846 38.588 1.00 28.88 C

ATOM 3262 H VAL B 232 9.976 0.528 40.230 1.00 0.00 H

ATOM 3263 HA VAL B 232 9.681 0.539 37.422 1.00 0.00 H

ATOM 3264 HB VAL B 232 8.175 -0.949 39.618 1.00 0.00 H

ATOM 3265 HG11 VAL B 232 7.372 -2.436 37.725 1.00 0.00 H

ATOM 3266 HG12 VAL B 232 6.564 -0.904 37.805 1.00 0.00 H

ATOM 3267 HG13 VAL B 232 7.833 -1.179 36.597 1.00 0.00 H

ATOM 3268 HG21 VAL B 232 9.532 -2.841 38.935 1.00 0.00 H

ATOM 3269 HG22 VAL B 232 10.200 -1.960 37.577 1.00 0.00 H

ATOM 3270 HG23 VAL B 232 10.619 -1.497 39.231 1.00 0.00 H

ATOM 3271 N ARG B 233 7.123 1.853 39.002 1.00 33.85 N

ATOM 3272 CA ARG B 233 5.878 2.574 38.780 1.00 32.76 C

ATOM 3273 C ARG B 233 6.063 4.021 38.311 1.00 33.46 C

ATOM 3274 O ARG B 233 5.150 4.599 37.727 1.00 37.10 O

ATOM 3275 CB ARG B 233 4.993 2.520 40.028 1.00 36.86 C

ATOM 3276 CG ARG B 233 5.585 3.184 41.251 1.00 33.87 C

ATOM 3277 CD ARG B 233 4.552 3.244 42.365 1.00 44.67 C

ATOM 3278 NE ARG B 233 4.919 4.188 43.415 1.00 44.05 N

ATOM 3279 CZ ARG B 233 5.254 3.837 44.652 1.00 55.30 C

ATOM 3280 NH1 ARG B 233 5.263 2.556 45.001 1.00 53.43 N

ATOM 3281 NH2 ARG B 233 5.572 4.769 45.542 1.00 54.23 N1+

ATOM 3282 H ARG B 233 7.415 1.649 39.947 1.00 0.00 H

ATOM 3283 HA ARG B 233 5.317 2.063 37.994 1.00 0.00 H

ATOM 3284 HB3 ARG B 233 4.761 1.479 40.259 1.00 0.00 H

ATOM 3285 HB2 ARG B 233 4.035 2.985 39.788 1.00 0.00 H

ATOM 3286 HG3 ARG B 233 5.920 4.197 41.029 1.00 0.00 H

ATOM 3287 HG2 ARG B 233 6.461 2.632 41.592 1.00 0.00 H

ATOM 3288 HD3 ARG B 233 4.327 2.244 42.732 1.00 0.00 H

ATOM 3289 HD2 ARG B 233 3.611 3.619 41.962 1.00 0.00 H

ATOM 3290 HE ARG B 233 4.904 5.166 43.165 1.00 0.00 H

ATOM 3291 HH12 ARG B 233 5.511 2.280 45.940 1.00 0.00 H

ATOM 3292 HH11 ARG B 233 5.010 1.847 44.329 1.00 0.00 H

ATOM 3293 HH22 ARG B 233 5.554 5.746 45.289 1.00 0.00 H

ATOM 3294 HH21 ARG B 233 5.820 4.510 46.486 1.00 0.00 H

ATOM 3295 N ASN B 234 7.235 4.604 38.553 1.00 30.60 N

ATOM 3296 CA ASN B 234 7.452 6.010 38.195 1.00 26.82 C

ATOM 3297 C ASN B 234 8.389 6.285 37.017 1.00 21.83 C

ATOM 3298 O ASN B 234 8.425 7.401 36.481 1.00 21.26 O

ATOM 3299 CB ASN B 234 7.895 6.805 39.423 1.00 23.83 C

ATOM 3300 CG ASN B 234 6.746 7.107 40.353 1.00 28.42 C

ATOM 3301 OD1 ASN B 234 5.617 7.299 39.909 1.00 34.86 O

ATOM 3302 ND2 ASN B 234 7.021 7.140 41.649 1.00 35.93 N

ATOM 3303 H ASN B 234 7.967 4.093 39.027 1.00 0.00 H

ATOM 3304 HA ASN B 234 6.543 6.474 37.816 1.00 0.00 H

ATOM 3305 HB3 ASN B 234 8.279 7.776 39.114 1.00 0.00 H

ATOM 3306 HB2 ASN B 234 8.710 6.307 39.941 1.00 0.00 H

ATOM 3307 HD22 ASN B 234 6.289 7.372 42.303 1.00 0.00 H

ATOM 3308 HD21 ASN B 234 7.965 6.988 41.973 1.00 0.00 H

ATOM 3309 N VAL B 235 9.146 5.281 36.598 1.00 22.95 N

ATOM 3310 CA VAL B 235 10.118 5.508 35.536 1.00 20.75 C

ATOM 3311 C VAL B 235 9.474 6.047 34.239 1.00 25.36 C

ATOM 3312 O VAL B 235 10.012 6.950 33.599 1.00 22.71 O

ATOM 3313 CB VAL B 235 11.000 4.260 35.286 1.00 24.21 C

ATOM 3314 CG1 VAL B 235 10.172 3.108 34.722 1.00 26.05 C

ATOM 3315 CG2 VAL B 235 12.175 4.606 34.367 1.00 20.10 C

ATOM 3316 H VAL B 235 9.114 4.380 37.057 1.00 0.00 H

ATOM 3317 HA VAL B 235 10.785 6.295 35.897 1.00 0.00 H

ATOM 3318 HB VAL B 235 11.412 3.942 36.244 1.00 0.00 H

ATOM 3319 HG11 VAL B 235 10.750 2.184 34.741 1.00 0.00 H

ATOM 3320 HG12 VAL B 235 9.265 2.930 35.298 1.00 0.00 H

ATOM 3321 HG13 VAL B 235 9.893 3.279 33.684 1.00 0.00 H

ATOM 3322 HG21 VAL B 235 12.875 3.775 34.343 1.00 0.00 H

ATOM 3323 HG22 VAL B 235 11.861 4.807 33.342 1.00 0.00 H

ATOM 3324 HG23 VAL B 235 12.724 5.477 34.726 1.00 0.00 H

ATOM 3325 N LYS B 236 8.296 5.543 33.882 1.00 23.85 N

ATOM 3326 CA LYS B 236 7.648 5.986 32.650 1.00 28.60 C

ATOM 3327 C LYS B 236 6.896 7.310 32.813 1.00 24.12 C

ATOM 3328 O LYS B 236 6.352 7.848 31.847 1.00 22.57 O

ATOM 3329 CB LYS B 236 6.728 4.894 32.100 1.00 28.99 C

ATOM 3330 CG LYS B 236 7.475 3.635 31.684 1.00 35.80 C

ATOM 3331 CD LYS B 236 6.553 2.620 31.028 1.00 41.55 C

ATOM 3332 CE LYS B 236 7.322 1.374 30.618 1.00 46.05 C

ATOM 3333 NZ LYS B 236 8.518 1.712 29.794 1.00 51.08 N1+

ATOM 3334 H LYS B 236 7.859 4.824 34.441 1.00 0.00 H

ATOM 3335 HA LYS B 236 8.403 6.166 31.881 1.00 0.00 H

ATOM 3336 HB3 LYS B 236 6.189 5.275 31.231 1.00 0.00 H

ATOM 3337 HB2 LYS B 236 5.967 4.642 32.841 1.00 0.00 H

ATOM 3338 HG3 LYS B 236 7.941 3.178 32.556 1.00 0.00 H

ATOM 3339 HG2 LYS B 236 8.280 3.930 31.011 1.00 0.00 H

ATOM 3340 HD3 LYS B 236 6.083 3.064 30.149 1.00 0.00 H

ATOM 3341 HD2 LYS B 236 5.745 2.346 31.708 1.00 0.00 H

ATOM 3342 HE3 LYS B 236 6.676 0.700 30.055 1.00 0.00 H

ATOM 3343 HE2 LYS B 236 7.655 0.830 31.503 1.00 0.00 H

ATOM 3344 HZ1 LYS B 236 9.141 2.305 30.324 1.00 0.00 H

ATOM 3345 HZ2 LYS B 236 9.003 0.864 29.536 1.00 0.00 H

ATOM 3346 HZ3 LYS B 236 8.226 2.196 28.957 1.00 0.00 H

ATOM 3347 N LYS B 237 6.896 7.844 34.030 1.00 20.85 N

ATOM 3348 CA LYS B 237 6.194 9.087 34.320 1.00 19.09 C

ATOM 3349 C LYS B 237 7.118 10.311 34.339 1.00 19.45 C

ATOM 3350 O LYS B 237 6.656 11.447 34.468 1.00 19.05 O

ATOM 3351 CB LYS B 237 5.451 8.970 35.654 1.00 22.13 C

ATOM 3352 CG LYS B 237 4.394 7.881 35.687 1.00 26.28 C

ATOM 3353 CD LYS B 237 3.779 7.791 37.076 1.00 34.83 C

ATOM 3354 CE LYS B 237 2.604 6.833 37.106 1.00 43.88 C

ATOM 3355 NZ LYS B 237 1.479 7.315 36.258 1.00 52.61 N1+

ATOM 3356 H LYS B 237 7.389 7.382 34.783 1.00 0.00 H

ATOM 3357 HA LYS B 237 5.435 9.297 33.565 1.00 0.00 H

ATOM 3358 HB3 LYS B 237 4.983 9.923 35.907 1.00 0.00 H

ATOM 3359 HB2 LYS B 237 6.177 8.770 36.440 1.00 0.00 H

ATOM 3360 HG3 LYS B 237 4.824 6.914 35.423 1.00 0.00 H

ATOM 3361 HG2 LYS B 237 3.640 8.106 34.933 1.00 0.00 H

ATOM 3362 HD3 LYS B 237 3.460 8.775 37.422 1.00 0.00 H

ATOM 3363 HD2 LYS B 237 4.533 7.447 37.772 1.00 0.00 H

ATOM 3364 HE3 LYS B 237 2.245 6.719 38.129 1.00 0.00 H

ATOM 3365 HE2 LYS B 237 2.907 5.843 36.764 1.00 0.00 H

ATOM 3366 HZ1 LYS B 237 1.789 7.396 35.300 1.00 0.00 H

ATOM 3367 HZ2 LYS B 237 0.713 6.659 36.308 1.00 0.00 H

ATOM 3368 HZ3 LYS B 237 1.169 8.218 36.588 1.00 0.00 H

ATOM 3369 N ILE B 238 8.422 10.080 34.202 1.00 18.96 N

ATOM 3370 CA ILE B 238 9.378 11.185 34.168 1.00 17.11 C

ATOM 3371 C ILE B 238 9.106 12.084 32.965 1.00 16.22 C

ATOM 3372 O ILE B 238 9.184 13.315 33.054 1.00 18.22 O

ATOM 3373 CB ILE B 238 10.823 10.666 34.122 1.00 17.08 C

ATOM 3374 CG1 ILE B 238 11.121 9.802 35.348 1.00 18.20 C

ATOM 3375 CG2 ILE B 238 11.809 11.834 34.018 1.00 16.49 C

ATOM 3376 CD1 ILE B 238 12.461 9.089 35.278 1.00 19.20 C

ATOM 3377 H ILE B 238 8.761 9.133 34.101 1.00 0.00 H

ATOM 3378 HA ILE B 238 9.251 11.782 35.073 1.00 0.00 H

ATOM 3379 HB ILE B 238 10.936 10.041 33.235 1.00 0.00 H

ATOM 3380 HG13 ILE B 238 10.358 9.037 35.484 1.00 0.00 H

ATOM 3381 HG12 ILE B 238 11.096 10.415 36.247 1.00 0.00 H

ATOM 3382 HG21 ILE B 238 12.837 11.488 34.041 1.00 0.00 H

ATOM 3383 HG22 ILE B 238 11.725 12.388 33.084 1.00 0.00 H

ATOM 3384 HG23 ILE B 238 11.684 12.538 34.842 1.00 0.00 H

ATOM 3385 HD11 ILE B 238 12.598 8.472 36.166 1.00 0.00 H

ATOM 3386 HD12 ILE B 238 12.510 8.418 34.422 1.00 0.00 H

ATOM 3387 HD13 ILE B 238 13.311 9.769 35.239 1.00 0.00 H

ATOM 3388 N NMA B 238A 8.738 11.531 31.755 1.00 0.00 N

ATOM 3389 CA NMA B 238A 8.450 12.394 30.618 1.00 0.00 C

ATOM 3390 H NMA B 238A 8.649 10.530 31.654 1.00 0.00 H

ATOM 3391 1HA NMA B 238A 9.320 12.998 30.352 1.00 0.00 H

ATOM 3392 2HA NMA B 238A 8.185 11.786 29.753 1.00 0.00 H

ATOM 3393 3HA NMA B 238A 7.612 13.059 30.833 1.00 0.00 H

TER 3394 NMA B 238A

HETATM 3395 O1 UNK 900 25.781 23.135 34.419 1.00 0.00 O

HETATM 3396 O2 UNK 900 25.789 25.123 35.596 1.00 0.00 O

HETATM 3397 O3 UNK 900 21.671 23.255 33.897 1.00 0.00 O

HETATM 3398 O4 UNK 900 23.135 20.839 33.367 1.00 0.00 O

HETATM 3399 O5 UNK 900 22.949 25.008 35.783 1.00 0.00 O

HETATM 3400 O6 UNK 900 26.840 28.860 38.428 1.00 0.00 O

HETATM 3401 O7 UNK 900 25.939 19.679 33.431 1.00 0.00 O

HETATM 3402 O8 UNK 900 28.032 29.642 40.232 1.00 0.00 O

HETATM 3403 O9 UNK 900 26.740 27.568 41.732 1.00 0.00 O

HETATM 3404 O10 UNK 900 25.916 24.714 40.459 1.00 0.00 O

HETATM 3405 O11 UNK 900 24.429 31.640 45.385 1.00 0.00 O

HETATM 3406 C1 UNK 900 22.910 22.960 34.501 1.00 0.00 C

HETATM 3407 C2 UNK 900 23.764 22.082 33.578 1.00 0.00 C

HETATM 3408 C3 UNK 900 23.668 24.251 34.835 1.00 0.00 C

HETATM 3409 C4 UNK 900 25.164 21.872 34.177 1.00 0.00 C

HETATM 3410 C5 UNK 900 25.073 23.929 35.370 1.00 0.00 C

HETATM 3411 C6 UNK 900 26.033 21.072 33.210 1.00 0.00 C

HETATM 3412 C7 UNK 900 26.832 29.134 39.796 1.00 0.00 C

HETATM 3413 C8 UNK 900 25.963 25.623 36.877 1.00 0.00 C

HETATM 3414 C9 UNK 900 26.362 26.826 39.388 1.00 0.00 C

HETATM 3415 C10 UNK 900 26.491 27.555 38.221 1.00 0.00 C

HETATM 3416 C11 UNK 900 26.652 27.730 40.513 1.00 0.00 C

HETATM 3417 C12 UNK 900 25.680 30.141 40.039 1.00 0.00 C

HETATM 3418 C13 UNK 900 26.300 26.979 36.967 1.00 0.00 C

HETATM 3419 C14 UNK 900 25.837 24.872 38.066 1.00 0.00 C

HETATM 3420 C15 UNK 900 26.038 25.467 39.318 1.00 0.00 C

HETATM 3421 C16 UNK 900 25.358 30.532 41.485 1.00 0.00 C

HETATM 3422 C17 UNK 900 24.388 29.825 42.205 1.00 0.00 C

HETATM 3423 C18 UNK 900 26.003 31.623 42.079 1.00 0.00 C

HETATM 3424 C19 UNK 900 24.079 30.194 43.512 1.00 0.00 C

HETATM 3425 C20 UNK 900 25.693 31.993 43.386 1.00 0.00 C

HETATM 3426 C21 UNK 900 24.733 31.276 44.099 1.00 0.00 C

HETATM 3427 H1 UNK 900 22.688 22.417 35.421 1.00 0.00 H

HETATM 3428 H2 UNK 900 23.854 22.554 32.599 1.00 0.00 H

HETATM 3429 H3 UNK 900 23.751 24.872 33.943 1.00 0.00 H

HETATM 3430 H4 UNK 900 25.109 21.297 35.102 1.00 0.00 H

HETATM 3431 H5 UNK 900 25.015 23.409 36.326 1.00 0.00 H

HETATM 3432 H6 UNK 900 25.780 21.288 32.172 1.00 0.00 H

HETATM 3433 H7 UNK 900 27.083 21.331 33.348 1.00 0.00 H

HETATM 3434 H8 UNK 900 21.117 22.472 33.939 1.00 0.00 H

HETATM 3435 H9 UNK 900 22.227 20.901 33.672 1.00 0.00 H

HETATM 3436 H10 UNK 900 22.374 24.415 36.272 1.00 0.00 H

HETATM 3437 H11 UNK 900 25.914 31.053 39.485 1.00 0.00 H

HETATM 3438 H12 UNK 900 24.776 29.751 39.566 1.00 0.00 H

HETATM 3439 H13 UNK 900 26.410 27.568 36.067 1.00 0.00 H

HETATM 3440 H14 UNK 900 25.589 23.822 38.040 1.00 0.00 H

HETATM 3441 H15 UNK 900 26.335 19.478 34.282 1.00 0.00 H

HETATM 3442 H16 UNK 900 28.330 30.290 39.588 1.00 0.00 H

HETATM 3443 H17 UNK 900 23.872 28.988 41.756 1.00 0.00 H

HETATM 3444 H18 UNK 900 26.742 32.189 41.531 1.00 0.00 H

HETATM 3445 H19 UNK 900 26.223 25.171 41.245 1.00 0.00 H

HETATM 3446 H20 UNK 900 23.332 29.641 44.063 1.00 0.00 H

HETATM 3447 H21 UNK 900 26.197 32.835 43.839 1.00 0.00 H

HETATM 3448 H22 UNK 900 23.740 31.097 45.775 1.00 0.00 H

CONECT 1 4 5 6

CONECT 4 1

CONECT 5 1

CONECT 6 1

CONECT 1682 1685 1686 1687

CONECT 1685 1682

CONECT 1686 1682

CONECT 1687 1682

CONECT 3395 3409 3410

CONECT 3396 3410 3413

CONECT 3397 3406 3434

CONECT 3398 3407 3435

CONECT 3399 3408 3436

CONECT 3400 3412 3415

CONECT 3401 3411 3441

CONECT 3402 3412 3442

CONECT 3403 3416

CONECT 3403 3416

CONECT 3404 3420 3445

CONECT 3405 3426 3448

CONECT 3406 3397 3407 3408 3427

CONECT 3407 3398 3406 3409 3428

CONECT 3408 3399 3406 3410 3429

CONECT 3409 3395 3407 3411 3430

CONECT 3410 3395 3396 3408 3431

CONECT 3411 3401 3409 3432 3433

CONECT 3412 3400 3402 3416 3417

CONECT 3413 3396 3418 3419

CONECT 3413 3419

CONECT 3414 3415 3416 3420

CONECT 3414 3420

CONECT 3415 3400 3414 3418

CONECT 3415 3418

CONECT 3416 3403 3412 3414

CONECT 3416 3403

CONECT 3417 3412 3421 3437 3438

CONECT 3418 3413 3415 3439

CONECT 3418 3415

CONECT 3419 3413 3420 3440

CONECT 3419 3413

CONECT 3420 3404 3414 3419

CONECT 3420 3414

CONECT 3421 3417 3422 3423

CONECT 3421 3422

CONECT 3422 3421 3424 3443

CONECT 3422 3421

CONECT 3423 3421 3425 3444

CONECT 3423 3425

CONECT 3424 3422 3426 3446

CONECT 3424 3426

CONECT 3425 3423 3426 3447

CONECT 3425 3423

CONECT 3426 3405 3424 3425

CONECT 3426 3424

CONECT 3427 3406

CONECT 3428 3407

CONECT 3429 3408

CONECT 3430 3409

CONECT 3431 3410

CONECT 3432 3411

CONECT 3433 3411

CONECT 3434 3397

CONECT 3435 3398

CONECT 3436 3399

CONECT 3437 3417

CONECT 3438 3417

CONECT 3439 3418

CONECT 3440 3419

CONECT 3441 3401

CONECT 3442 3402

CONECT 3443 3422

CONECT 3444 3423

CONECT 3445 3404

CONECT 3446 3424

CONECT 3447 3425

CONECT 3448 3405

ENDMDL

END
