# Supplementary material for: Phytochemical analysis, biological activities of methanolic extracts and an isolated flavonoid from Tunisian Limoniastrum monopetalum (L.) Boiss: an in vitro and in silico investigations
Source: Sci Rep. 2023 Nov 6;13:19144. doi: 10.1038/s41598-023-46457-6 (PMC10628221; doi:10.1038/s41598-023-46457-6)
Supplement: Supplementary file 1 — Supplementary Information. [file 41598_2023_46457_MOESM1_ESM.zip › Supplementary files/Supplementary data.docx]

**Supporting Information**

**A Phytochemical Analysis, Biological Activities and Molecular Interaction of Methanolic Extract of Tunisian *Limoniastrum monopetalum* (L.) Boiss: An *In Vitro* and *In Silico* Investigations**

**Amel Bouzidi^1^, Ahmed Azizi^2^, Omar Messaoudi^3,4^, Kirouani Abderrezzak^1^, Giovanni Vidari^5^, Ahmed Noureddine Hellal^6^ and *Chirag N. Patel^7,8^**

^1^University of Medea, Faculty of Sciences, Department of Biology, BTP laboratory, Algeria.

^2^University Amar Telidji, Faculty of Technology, Highway Ghardaia post box G37 (M'kam) 03000, Laghouat, Algeria.

^3^Laboratory of Applied Microbiology in Food, Biomedical and Environment, Abou Bekr Belkaïd University, 13000 Tlemcen, Algeria.

^4^Department of Biology, Faculty of Science, University of Amar Telidji, 03000 Laghouat, Algeria.

^5^Department of Medical Analysis, Faculty of Applied Science, Ishk International University,

Erbil 44001, Iraq.

^6^University of Monastir, Higher Institute of Biotechnology of Monastir, Laboratory of Bioressources, Biology Integrative and Valorization, Tunisia.

^7^Department of Botany, Bioinformatics and Climate Change Impacts Management, School of Science, Gujarat University, Ahmedabad-380 009, India.

^8^Biotechnology Research Center, Technology Innovation Institute, Abu Dhabi 9639, United Arab Emirates.

**Details of correspondence**

Dr. Chirag N. Patel

Department of Botany, Bioinformatics, and Climate Change Impacts Management,

School of Sciences, Gujarat University, Ahmedabad 380009, Gujarat, India.

Biotechnology Research Center, Technology Innovation Institute, Abu Dhabi 9639, United Arab Emirates

Email: [chiragpatel269@gmail.com](mailto:chiragpatel269@gmail.com)


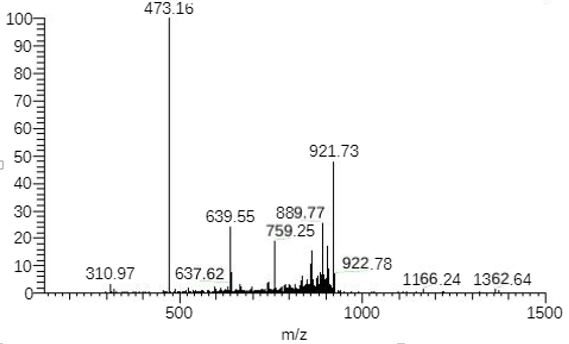


**Figure S1:** LC/UV/MS Spectrum of maesopsin-6-*O*-β-D-glucopyranoside (**1**).


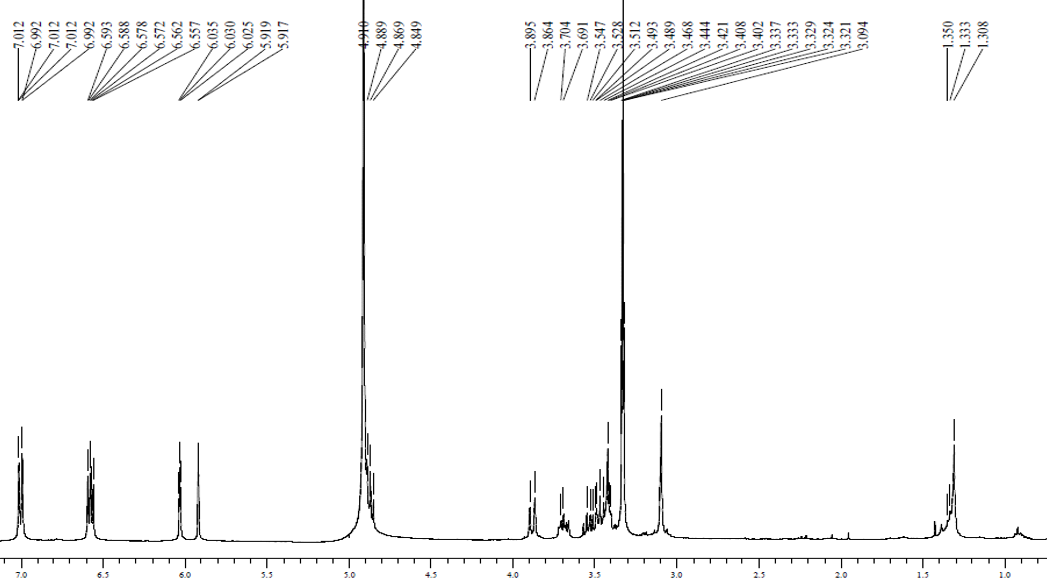


**Figure S2.** ^1^H NNMR spectrum (400 MHz) of maesopsin-6-*O*-D-glucopyranoside (**1**), in CD_3_OD.

**Table S1.** ^1^H NMR data (400 MHz, CD_3_OD) of maesopsin-6-*O*-β-D-glucopyranoside (**1**).

| H | *δ*_H_ |
| --- | --- |
| 5 | 5.90 (1H, *d, J* = 1.5 Hz) |
| 7 | 6.05 (1H, *d, J* = 1.5 Hz) |
| 2̕ , 6΄ | 7.00 (2H, *d, J* = 8.4 Hz) |
| 3̕ , 5̕ | 6.57, 6.58 (2H, *d* each*, J* = 8.4 Hz) |
| α | 3.09 (2H, *s*) |
| 1" | 4.85, 4.88 (1H, *d* each, *J* = 8 Hz) |
| 2"-6‘‘ | 3.35-3.90 (6H, *multiplets*) |
